# Supplementary material for: TRIPOD statement: a preliminary pre-post analysis of reporting and methods of prediction models
Source: BMJ Open. 2020 Sep 18;10(9):e041537. doi: 10.1136/bmjopen-2020-041537 (PMC7511612; doi:10.1136/bmjopen-2020-041537)
Supplement: Supplementary data [file bmjopen-2020-041537supp001.pdf]

Supplementary documents

The impact of the TRIPOD statement: a pre-post analysis of reporting and methods.

Amir H Zamanipoor Najafabadi BSc[1,2,3], Chava L Ramspek MD[3], Friedo W. Dekker[3], Pauline Heus MSc[4,5], Lotty Hooft PhD[4,5], Karel G M Moons MD PhD[4,5], Wilco C Peul MD PhD[1], Gary S Collins PhD[6,7], Ewout W Steyerberg PhD[8], Merel van Diepen PhD[3].

[1]Department of Neurosurgery, University Neurosugrical Center Holland, Leiden University Medical Center & Haaglanden Medical Center & Haga Teaching Hospitals, Leiden & the Hageue, the Netherlands
[2]Department of Neurology, Leiden University Medical Center, Leiden, the Netherlands
[3]Department of Clinical Epidemiology, Leiden University Medical Center, Leiden, the Netherlands
[4]Cochrane Netherlands, University Medical Center Utrecht, Utrecht University
[5]Julius Center for Health Sciences and Primary Care, University Medical Center Utrecht, Utrecht University
[6]Centre for Statistics in Medicine, Nuffield Department of Orthopaedics, Rheumatology and Musculoskeletal Sciences, University of Oxford, Oxford, UK.
[7]National Institute for Health Research Oxford Biomedical Research Centre, John Radcliffe Hospital, Oxford, UK
[8]Department of Biomedical Data Sciences, Leiden University Medical Center, Leiden, The Netherlands.

Corresponding author: Amir H. Zamanipoor Najafabadi, Department of Neurosurgery, Neurology and Clinical Epidemiology, Leiden University Medical Center, Leiden, the Netherlands. *Tel:* +31-(0)71-5262109. Fax: +31-(0)71-5266987. *Email:* a.h.zamanipoor_najafabadi@lumc.nl

Supplementary Table 1: TRIPOD reporting scores for all included articles

	Before % n=32	After % n=38	Absolute difference of percentages (95% CI)
**Title and abstract**	25	25	0 (-15 to 15)
**Introduction**	69	83	14 (-3 to 31)
**Methods**	80	83	3 (-2 to 7)
**Results**	66	67	1 (-10 to 11)
**Discussion**	89	85	-4 (-13 to 5)
**Other information**	100	97	-3 (-8 to 3)
**Total**	74	76	2 (-4 to 7)

Supplementary Table 2: TRIPOD reporting scores for individual TRIPOD items

TRIPOD item	pre-TRIPOD %	post-TRIPOD %
**1**	34	42
**2**	16	8
**3a**	78	84
**3b**	59	82
**4a**	97	100
**4b**	91	95
**5a**	97	95
**5b**	97	97
**5c**	100	60
**6a**	97	97
**6b**	97	95
**7a**	88	87
**7b**	94	100
**8**	100	100
**9**	28	24
**10a**	63	84
**10b**	19	39
**10c**	67	100
**10d**	69	71
**10e**	56	50
**11**	96	95
**12**	75	71
**13a**	94	95
**13b**	50	37
**13c**	53	65
**14a**	100	97
**14b**	86	64
**15a**	27	42
**15b**	65	61
**16**	66	68
**17**	40	73
**18**	94	97
**19a**	82	75
**19b**	100	95
**20**	75	68
**22**	100	97

Supplementary Table 3: TRIPOD reporting scores for articles published after TRIPOD statement referring vs not referring to the statement

	TRIPOD not referred % n=18	TRIPOD referred % n=20	Absolute difference of percentages (95% CI)
**Title and abstract**	33	18	-16 (-36 to 4)
**Introduction**	86	80	-6 (-28 to 16)
**Methods**	82	84	2 (-3 to 8)
**Results**	67	67	0 (-17 to 17)
**Discussion**	87	83	-3 (-15 to 9)
**Other information**	94	100	6 (-6 to 17)
**Total**	76	76	-1 (-8 to 7)

Supplementary Table 4: TRIPOD reporting scores for articles published after TRIPOD statement in journals that published and did not publish the TRIPOD statement

	TRIPOD not endorsed % n=17	TRIPOD endorsed % n=21	Absolute difference of percentages (95% CI)
**Title and abstract**	24	26	3 (-17 to 22)
**Introduction**	85	81	-4 (-26 to 18)
**Methods**	84	82	-3 (-8 to 3)
**Results**	71	63	-7 (-24 to 9)
**Discussion**	86	84	-2 (-15 to 11)
**Other information**	100	95	-5 (-15 to 5)
**Total**	78	75	-3 (-11 to 4)

Supplementary Table 5: TRIPOD reporting scores for articles published after TRIPOD statement in journals that require adherence to the TRIPOD statement and journals that do not require to the adherence statement

	TRIPOD not required % n=16	TRIPOD required % n=22	Absolute difference of percentages (95% CI)
**Title and abstract**	25	25	0 (-21 to 21)
**Introduction**	81	84	3 (-20 to 26)
**Methods**	83	83	0 (-6 to 6)
**Results**	66	67	1 (-16 to 19)
**Discussion**	90	81	-8 (-20 to 3)
**Other information**	100	95	-5 (-15 to 5)
**Total**	76	76	0 (-8 to 9)

Supplementary Table 6: Percentage articles reporting TRIPOD items in supplementary material

TRIPOD item	Supplement %
**1**	0
**2**	0
**3a**	0
**3b**	0
**4a**	7
**4b**	7
**5a**	10
**5b**	6
**5c**	33
**6a**	13
**6b**	0
**7a**	36
**7b**	0
**8**	3
**9**	9
**10a**	12
**10b**	13
**10c**	14
**10d**	1
**10e**	0
**11**	4
**12**	24
**13a**	21
**13b**	30
**3c**	17
**14a**	2
**14b**	40
**15a**	40
**15b**	35
**16**	0
**17**	17
**18**	0
**19a**	0
**19b**	0
**20**	0
**22**	1

Supplementary figure 1: Average overall TRIPOD reporting levels in percentage per year

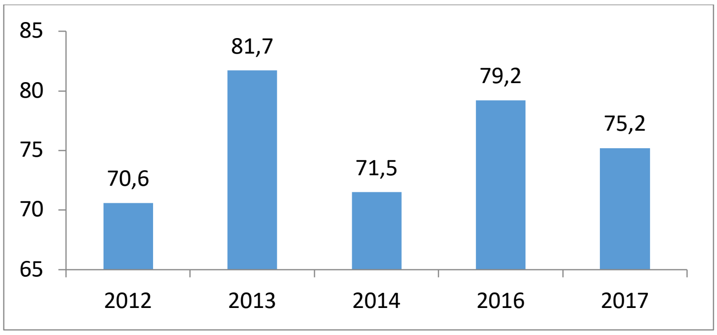

Supplementary Table 7: Performance measures and missing data in all studies

	**Before 2015 (n=32) Number (%)**	**After 2016 (n=38) Number (%)**
**Calibration**		
Plot	16 (50%)	31 (82%)
Intercept and Slope	0 (0%)	0 (0%)
Calibration in-the-large	1 (3%)	0 (0%)
Slope	0 (0%)	1 (3%)
Test	4 (13%)	1 (3%)
Not	11 (34%)	5 (13%)
**Discrimination**		
C-statistic / AUC	29 (91%)	38 (100%)
D-statistic	5 (16%)	5 (13%)
Not	3 (9%)	0 (0%)
**Classification**		
IDI	5 (16%)	4 (11%)
NRI	8 (25%)	7 (18%)
Sens. Spec. PPV. NPV. LR. ROC	20 (63%)	19 (50%)
Not reported	10 (31%)	16 (42%)
**Clinical usefulness**		
Decision curve analysis	2 (6%)	8 (21%)
Not reported	30 (94%)	30 (79%)
**Overall performance**		
Brier	2 (6%)	3 (8%)
R2	4 (13%)	5 (13%)
Adequacy statistic	1 (3%)	2 (5%)
Not reported	26 (81%)	28 (74%)
**Missing data reporting**		
Per variable	17 (53%)	13 (34%)
Overall	2 (6%)	14 (37%)
Not reported	13 (41%)	11 (29%)
**Type and reason of missing data**		
Type reported	3 (9%)	2 (5%)
Reason reported	5 (16%)	2 (5%)
**Missing data handling**		
Complete-case analysis	5 (16%)	6 (16%)
Multiple Imputation	12 (38%)	19 (50%)
Other methods	6 (19%)	4 (11%)
Not reported	9 (28%)	9 (24%)

BMJ Publishing Group Limited (BMJ) disclaims all liability and responsibility arising from any reliance placed on this supplemental material which has been supplied by the author(s)

Supplementary Table 8: Model development and presentation

	Before 2015 (n=24) Number (%)	After 2016 (n=27) Number (%)
**Sample per candidate predictor**		
<10	8 (33%)	8 (30%)
10-100	8 (33%)	9 (33%)
100-1000	8 (17%)	5 (19%)
>1000	3 (13%)	3 (11%)
Unknown number of predictors	0 (0%)	1 (4%)
Unknown number of outcomes	1 (4%)	1 (4%)
**Model Type**		
Linear	1 (4%)	0 (0%)
Logistic	16 (67%)	15 (56%)
Cox	6 (25%)	10 (37%)
Points	0 (0%)	2 (7%)
Other	1 (4%)	0 (0%)
**Predictor Selection**		
A priori knowledge / based on literature	11 (46%)	17 (63%)
Statistically	4 (17%)	2 (7%)
Not reported	9 (38%)	8 (30%)
**Model building**		
Entering all	6 (25%)	12 (44%)
Stepwise	15 (63%)	13 (48%)
Best subset	2 (8%)	1 (4%)
Other	1 (4%)	1 (4%)
**Model building thresholds**		
p-value	16 (67%)	12 (44%)
Effect measure	1 (4%)	3 (11%)
R	0 (0%)	1 (4%)
Manually	0 (0%)	1 (4%)
AIC	0 (0%)	4 (15%)
C-statistic	2 (8%)	0 (0%)
Other	0 (0%)	0 (0%)
Not used	5 (21%)	6 (22%)
**Internal validation**		
Random split	4 (17%)	7 (26%)
Cross validation	4 (17%)	2 (7%)
Bootstrapping	7 (29%)	11 (41%)
Not reported	9 (38%)	7 (26%)
**Model presentation**		
Coefficients	21 (88%)	23 (85%)
Intercept with coefficients	7 (29%)	11 (41%)
Application	9 (38%)	10 (37%)
Simplified score / nomogram	7 (29%)	7 (26%)
Not reported	2 (8%)	1 (4%)

Supplementary Table 9: External validation and updating

	Before 2015 (n=18) Number (%)	After 2016 (n=26) Number (%)
**External validation**		
Fully independent	14 (78%)	23 (88%)
Geographical	2 (11%)	0 (0%)
Temporal	2 (11%)	3 (12%)
**Model updating**		
Added marker	4 (22%)	1 (4%)
All coefficients independently	3 (17%)	5 (19%)
Only intercept	1 (6%)	4 (15%)
All coefficients with same factor	1 (6%)	0 (0%)
Not updated	9 (50%)	16 (62%)

Supplementary text 1: Protocol

Article selection

Search strategy:

("The New England journal of medicine"[Journal] OR "Lancet (London, England)"[Journal] OR "BMJ (Clinical research ed.)"[Journal] OR "*JAMA*"[Journal] OR "PLOS Medicine"[Journal] OR "*Annals of Internal Medicine*" [Journal] OR "*BMC Medicine*"[Journal]) AND (predict*[ti] OR prognost*[ti] OR diagnostic*[ti] OR "risk score"[ti]) NOT ("Animals"[mesh] NOT "Humans"[mesh]) NOT (("case reports"[ptyp] OR "case report"[ti]) NOT ("Review"[ptyp] OR "clinical study"[ptyp] OR "case series"[tw]))

Articles included

1 Abbasi A, Peelen LM, Corpeleijn E, *et al.* Prediction models for risk of developing type 2 diabetes: Systematic literature search and independent external validation study. *BMJ* 2012;**345**:1–16. doi:10.1136/bmj.e5900

2 Bejnordi BE, Veta M, Van Diest PJ, *et al.* Diagnostic assessment of deep learning algorithms for detection of lymph node metastases in women with breast cancer. *JAMA - J Am Med Assoc* 2017;**318**:2199–210. doi:10.1001/jama.2017.14585

3 Collins GS, Altman DG. Predicting the 10 year risk of cardiovascular disease in the United Kingdom: Independent and external validation of an updated version of QRISK2. *BMJ* 2012;**345**:1–12. doi:10.1136/bmj.e4181

4 Costa F, van Klaveren D, James S, *et al.* Derivation and validation of the predicting bleeding complications in patients undergoing stent implantation and subsequent dual antiplatelet therapy (PRECISE-DAPT) score: a pooled analysis of individual-patient datasets from clinical trials. *Lancet* 2017;**389**:1025–34. doi:10.1016/S0140-6736(17)30397-5

5 Cubiella J, Vega P, Salve M, *et al.* Development and external validation of a faecal immunochemical test-based prediction model for colorectal cancer detection in symptomatic patients. *BMC Med* 2016;**14**:1–13. doi:10.1186/s12916-016-0668-5

6 D.S. L, A. S, P.C. A, *et al.* Prediction of heart failure mortality in emergent care: A cohort study. *Ann Intern Med* 2012;**156**:767–75. doi:10.7326/0003-4819-156-11-201206050-00003

7 Dagan N, Cohen-Stavi C, Leventer-Roberts M, *et al.* External validation and comparison of three prediction tools for risk of osteoporotic fractures using data from population based electronic health records: Retrospective cohort study. *BMJ* 2017;**356**. doi:10.1136/bmj.i6755

8 Dalton JE, Perzynski AT, Zidar DA, *et al.* Accuracy of cardiovascular risk prediction varies by neighborhood socioeconomic position a retrospective cohort study. *Ann Intern Med* 2017;**167**:456–64. doi:10.7326/M16-2543

9 Dalziel SR, Thompson JMD, Macias CG, *et al.* Predictors of severe H1N1 infection in children presenting within Pediatric Emergency Research Networks (PERN): Retrospective case-control study. *BMJ* 2013;**347**:1–13. doi:10.1136/bmj.f4836

10 Terfc. Glycated hemoglobin measurement and prediction of cardiovascular disease. *JAMA - J Am Med Assoc* 2014;**311**:1225–33. doi:10.1001/jama.2014.1873

11 Elias SG, Kok L, de Wit NJ, *et al.* Is there an added value of faecal calprotectin and haemoglobin in the diagnostic work-up for primary care patients suspected of significant colorectal disease? A cross-sectional diagnostic study. *BMC Med* 2016;**14**:1–11. doi:10.1186/s12916-016-0684-5

12 Kaptoge. C-Reactive Protein, Fibrinogen, and Cardiovascular Disease Prediction. *N Engl J Med* 2012;**367**:1310–20. doi:10.1056/NEJMoa1107477

13 Esplin MS, Elovitz MA, Iams JD, *et al.* Predictive accuracy of serial transvaginal cervical lengths and quantitative vaginal fetal fibronectin levels for spontaneous preterm birth among nulliparous women. *JAMA - J Am Med Assoc* 2017;**317**:1047–56. doi:10.1001/jama.2017.1373

14 Fischer K, Kettunen J, Würtz P, *et al.* Biomarker Profiling by Nuclear Magnetic Resonance Spectroscopy for the Prediction of All-Cause Mortality: An Observational Study of 17,345 Persons. *PLoS Med* 2014;**11**. doi:10.1371/journal.pmed.1001606

15 Fraccaro P, van der Veer S, Brown B, *et al.* An external validation of models to predict the onset of chronic kidney disease using population-based electronic health records from Salford, UK. *BMC Med* 2016;**14**:1–15. doi:10.1186/s12916-016-0650-2

16 Freund Y, Lemachatti N, Krastinova E, *et al.* Prognostic accuracy of sepsis-3 criteria for in-hospital mortality among patients with suspected infection presenting to the emergency department. *JAMA - J Am Med Assoc* 2017;**317**:301–8. doi:10.1001/jama.2016.20329

17 Ganz P, Heidecker B, Hveem K, *et al.* Development and validation of a protein-based risk score for cardiovascular outcomes among patients with stable coronary heart disease. *JAMA - J Am Med Assoc* 2016;**315**:2532–41. doi:10.1001/jama.2016.5951

18 Genders TSS, Steyerberg EW, Hunink MGM, *et al.* Prediction model to estimate presence of coronary artery disease: Retrospective pooled analysis of existing cohorts. *BMJ* 2012;**344**:1–13. doi:10.1136/bmj.e3485

19 Gnanapragasam VJ, Lophatananon A, Wright KA, *et al.* Improving Clinical Risk Stratification at Diagnosis in Primary Prostate Cancer: A Prognostic Modelling Study. *PLoS Med* 2016;**13**:1–18. doi:10.1371/journal.pmed.1002063

20 Goldstick JE, Carter PM, Walton MA, *et al.* Development of the SaFETy score: A clinical screening tool for predicting future firearm violence risk. *Ann Intern Med* 2017;**166**:707–14. doi:10.7326/M16-1927

21 Goodacre S, Wilson R, Shephard N, *et al.* Derivation and validation of a risk adjustment model for predicting seven day mortality in emergency medical admissions: Mixed prospective and retrospective cohort study. *BMJ* 2012;**344**:1–11. doi:10.1136/bmj.e2904

22 den ruitjer H, Rembold CM. Common Carotid Intima-Media Thickness Measurements in Cardiovascular Risk Prediction. *J Am Med Assoc* 2015;**308**:796–803.http://jama.jamanetwork.com/

23 Hijazi Z, Oldgren J, Lindbäck J, *et al.* The novel biomarker-based ABC (age, biomarkers, clinical history)-bleeding risk score for patients with atrial fibrillation: a derivation and validation study. *Lancet* 2016;**387**:2302–11. doi:10.1016/S0140-6736(16)00741-8

24 Hippisley-Cox J, Coupland C. Development and validation of risk prediction equations to estimate survival in patients with colorectal cancer: Cohort study. *BMJ* 2017;**357**. doi:10.1136/bmj.j2497

25 Hippisley-Cox J, Coupland C, Brindle P. Development and validation of QRISK3 risk prediction algorithms to estimate future risk of cardiovascular disease: Prospective cohort study. *BMJ* 2017;**357**. doi:10.1136/bmj.j2099

26 Hippisley-Cox J. Predicting risk of upper gastrointestinal bleed and intracranial bleed with anticoagulants: Cohort study to derive and validate the QBleed scores. *BMJ* 2014;**349**:1–21. doi:10.1136/bmj.g4606

27 Hippisley-Cox J, Coupland C, Brindle P. Derivation and validation of QStroke score for predicting risk of ischaemic stroke in primary care and comparison with other risk scores: A prospective open cohort study. *BMJ* 2013;**346**:1–15. doi:10.1136/bmj.f2573

28 Hippisley-Cox J, Coupland C. Derivation and validation of updated QFracture algorithm to predict risk of osteoporotic fracture in primary care in the United Kingdom: Prospective open cohort study. *BMJ* 2012;**345**:1–16. doi:10.1136/bmj.e3427

29 Hippisley-Cox J, Coupland C. Development and validation of QMortality risk prediction algorithm to estimate short term risk of death and assess frailty: cohort study. *BMJ* 2017;**358**:j4208. doi:10.1136/bmj.j4208

30 J.M. Q, C. EE, A. U, *et al.* Predictive score for mortality in patients with COPD exacerbations attending hospital emergency departments. *BMC Med* 2014;**12**:66. doi:10.1186/1741-7015-12-66

31 James MT, Pannu N, Hemmelgarn BR, *et al.* Derivation and external validation of prediction models for advanced chronic kidney disease following acute kidney injury. *JAMA - J Am Med Assoc* 2017;**318**:1787–97. doi:10.1001/jama.2017.16326

32 Kavousi M, Desai CS, Ayers C, *et al.* Prevalence and prognostic implications of coronary artery calcification in low-risk women: A meta-analysis. *JAMA - J Am Med Assoc* 2016;**316**:2126–34.

BMJ Publishing Group Limited (BMJ) disclaims all liability and responsibility arising from any reliance placed on this supplemental material which has been supplied by the author(s)

doi:10.1001/jama.2016.17020

33 Kent M, Penson DF, Albertsen PC, *et al.* Successful external validation of a model to predict other cause mortality in localized prostate cancer. *BMC Med* 2016;**14**:1–7. doi:10.1186/s12916-016-0572-z

34 Perel P. Predicting early death in patients with traumatic bleeding: development and validation of prognostic model. *J Geophys Res* 1989;**94**:3579. doi:10.1136/bmj.e5166

35 Kugathasan S, Denson LA, Walters TD, *et al.* Prediction of complicated disease course for children newly diagnosed with Crohn’s disease: a multicentre inception cohort study. *Lancet* 2017;**389**:1710–8. doi:10.1016/S0140-6736(17)30317-3

36 Lamain-de Ruiter M, Kwee A, Naaktgeboren CA, *et al.* External validation of prognostic models to predict risk of gestational diabetes mellitus in one Dutch cohort: prospective multicentre cohort study. *BMJ* 2016;**354**:i4338. doi:10.1136/bmj.i4338

37 Little P, Stuart B, Richard Hobbs FD, *et al.* Predictors of suppurative complications for acute sore throat in primary care: Prospective clinical cohort study. *BMJ* 2013;**347**:1–14. doi:10.1136/bmj.f6867

38 Wang s w. Anti-allergic activity of some selected plants in the genus Boesenbergia and Kaempferia. *Songklanakarin J Sci Technol* 2011;**33**:301–4. doi:10.1136/bmj.i2607

39 Dewland TA. Atrial Ectopy as a Predictor of Incident Atrial Fibrillation. 2011;**4**:721–8. doi:10.1126/scisignal.2001449.Engineering

40 Marcantonio ER, Ngo LH, O’Connor M, *et al.* 3D-CAM: Derivation and validation of a 3-minute diagnostic interview for CAM-defined delirium: A cross-sectional diagnostic test study. *Ann Intern Med* 2014;**161**:554–61. doi:10.7326/M14-0865

41 Matsushita K, Woodward M, Jafar TH, *et al.* Comparison of Risk Prediction Using the CKD-EPI Equation and the MDRD Study Equation for Estimated Glomerular Filtration Rate. 2012;**307**:1941–51.

42 McLernon DJ, Steyerberg EW, Te Velde ER, *et al.* Predicting the chances of a live birth after one or more complete cycles of in vitro fertilisation: Population based study of linked cycle data from 113 873 women. *BMJ* 2016;**355**. doi:10.1136/bmj.i5735

43 Miro O, Rossello X, Gil V, *et al.* Predicting 30-day mortality for patients with acute heart failure in the emergency department. *Ann Intern Med* 2017;**167**:698–705. doi:10.7326/M16-2726

44 Moore CL, Bomann S, Daniels B, *et al.* Derivation and validation of a clinical prediction rule for uncomplicated ureteral stone-the STONE score: Retrospective and prospective observational cohort studies. *BMJ* 2014;**348**:1–12. doi:10.1136/bmj.g2191

45 Mwangi MN, Maskey S, Andang’o PEA, *et al.* Diagnostic utility of zinc protoporphyrin to detect iron deficiency in Kenyan pregnant women. *BMC Med* 2014;**12**:1–13. doi:10.1186/s12916-014-0229-8

46 Nijman RG, Vergouwe Y, Thompson M, *et al.* Clinical prediction model to aid emergency doctors managing febrile children at risk of serious bacterial infections: Diagnostic study. *BMJ* 2013;**346**:1–16. doi:10.1136/bmj.f1706

47 Koller MT. Original Research Older U . S . and European Persons in the Cardiovascular Health Study. *Ann Intern Med* 2012.

48 Patel VB, Theron G, Lenders L, *et al.* Diagnostic Accuracy of Quantitative PCR (Xpert MTB/RIF) for Tuberculous Meningitis in a High Burden Setting: A Prospective Study. *PLoS Med* 2013;**10**. doi:10.1371/journal.pmed.1001536

49 Pavese C, Schneider MP, Schubert M, *et al.* Prediction of Bladder Outcomes after Traumatic Spinal Cord Injury: A Longitudinal Cohort Study. *PLoS Med* 2016;**13**:1–16. doi:10.1371/journal.pmed.1002041

50 Payne BA, Hutcheon JA, Ansermino JM, *et al.* A Risk Prediction Model for the Assessment and Triage of Women with Hypertensive Disorders of Pregnancy in Low-Resourced Settings: The miniPIERS (Pre-eclampsia Integrated Estimate of RiSk) Multi-country Prospective Cohort Study. *PLoS Med* 2014;**11**. doi:10.1371/journal.pmed.1001589

51 Pfeiffer RM, Park Y, Kreimer AR, *et al.* Risk Prediction for Breast, Endometrial, and Ovarian Cancer in White Women Aged 50 y or Older: Derivation and Validation from Population-Based Cohort Studies. *PLoS Med* 2013;**10**. doi:10.1371/journal.pmed.1001492

52 Q. L, M. IDK, T.K. T, *et al.* A clinical prediction rule for diagnosing human infections with avian influenza A(H7N9) in a hospital emergency department setting. *BMC Med* 2014;**12**:1–9. doi:10.1186/s12916-014-0127-0 LK - http://sfx.hul.harvard.edu/sfx_local?sid=EMBASE&issn=17417015&id=doi:10.1186%2Fs12916-014-0127-0&atitle=A+clinical+prediction+rule+for+diagnosing+human+infections+with+avian+influenza+A%28H7N9%29+in+a+hospital+emergency+department+setting&stitle=BMC+Med.&title=BMC+Medicine&volume=12&issue=1&spage=&epage=&aulast=Liao&aufirst=Qiaohong&auinit=Q.&aufull=Liao+Q.&coden=&isbn=&pages=-&date=2014&auinit1=Q&auinitm=

53 Raith EP, Udy AA, Bailey M, *et al.* Prognostic accuracy of the SOFA score, SIRS criteria, and qSOFA score for in-hospital mortality among adults with suspected infection admitted to the intensive care unit. *JAMA - J Am Med Assoc* 2017;**317**:290–300. doi:10.1001/jama.2016.20328

54 Pandie S. Diagnostic accuracy of quantitative PCR (Xpert MTB/RIF) for tuberculous pericarditis compared to adenosine deaminase and unstimulated interferon-γ in a high burden setting: A prospective study. *BMC Med* 2014;**12**:2–11. doi:10.1186/1741-7015-12-101

55 Raji O y. Annals of Internal Medicine Predictive Accuracy of the Liverpool Lung Project Risk Model for Stratifying Patients for Computed Tomography Screening for Lung Cancer. 2012.

56 Sultan AA, West J, Grainge MJ, *et al.* Development and validation of risk prediction model for venous thromboembolism in postpartum women: Multinational cohort study. *BMJ* 2016;**355**. doi:10.1136/bmj.i6253

57 Tangri N, Grams ME, Levey AS, *et al.* Multinational assessment of accuracy of equations for predicting risk of kidney failure ameta-analysis. *JAMA - J Am Med Assoc* 2016;**315**:164–74. doi:10.1001/jama.2015.18202

58 ten Haaf K, Jeon J, Tammemägi MC, *et al.* Risk prediction models for selection of lung cancer screening candidates: A retrospective validation study. *PLoS Med* 2017;**14**:1–24. doi:10.1371/journal.pmed.1002277

59 Thangaratinam S, Allotey J, Marlin N, *et al.* Prediction of complications in early-onset pre-eclampsia (PREP): Development and external multinational validation of prognostic models. *BMC Med* 2017;**15**:1–11. doi:10.1097/QAD.0b013e3282efacb1

60 Traeger AC, Henschke N, Hübscher M, *et al.* Estimating the Risk of Chronic Pain: Development and Validation of a Prognostic Model (PICKUP) for Patients with Acute Low Back Pain. *PLoS Med* 2016;**13**:1–21. doi:10.1371/journal.pmed.1002019

61 Van Calster B, Van Hoorde K, Valentin L, *et al.* Evaluating the risk of ovarian cancer before surgery using the ADNEX model to differentiate between benign, borderline, early and advanced stage invasive, and secondary metastatic tumours: Prospective multicentre diagnostic study. *BMJ* 2014;**349**:1–14. doi:10.1136/bmj.g5920

62 Van Den Boogaard M, Pickkers P, Slooter AJC, *et al.* Development and validation of PRE-DELIRIC (PREdiction of DELIRium in ICu patients) delirium prediction model for intensive care patients: Observational multicentre study. *BMJ* 2012;**344**:17. doi:10.1136/bmj.e420

63 Van den Bruel A, Thompson M, Stevens R, *et al.* How well do clinical prediction rules perform in identifying serious infections in acutely ill children across an international network of ambulatory care datasets?. *BMC Med* 2013;**11**:10.http://ovidsp.ovid.com/ovidweb.cgi?T=JS&PAGE=reference&D=medc&NEWS=N&AN=23320738

64 Van Vugt SF, Broekhuizen BDL, Lammens C, *et al.* Use of serum C reactive protein and procalcitonin concentrations in addition to symptoms and signs to predict pneumonia in patients presenting to primary care with acute cough: Diagnostic study. *BMJ* 2013;**346**:1–12. doi:10.1136/bmj.f2450

65 Viera AJ, Garrett JM. Understanding interobserver agreement: The kappa statistic. *Fam Med* 2005;**37**:360–3.

66 W.A. L, R. D, L.A. K, *et al.* Serum protein profiles predict coronary artery disease in symptomatic patients referred for coronary angiography. *BMC Med* 2012;**10**:157. doi:10.1186/1741-7015-10-157

67 Walters K, Hardoon S, Petersen I, *et al.* Predicting dementia risk in primary care: Development and

validation of the Dementia Risk Score using routinely collected data. *BMC Med* 2016;**14**:1–12. doi:10.1186/s12916-016-0549-y

68 Yeh RW, Secemsky EA, Kereiakes DJ, *et al.* Development and validation of a prediction rule for benefit and harm of Dual antiplatelet therapy beyond 1 year after percutaneous coronary intervention. *JAMA - J Am Med Assoc* 2016;**315**:1735–49. doi:10.1001/jama.2016.3775

69 Zemek R, Barrowman N, Freedman SB, *et al.* Clinical risk score for persistent postconcussion symptomsamong children with acute concussion in the ED. *JAMA - J Am Med Assoc* 2016;**315**:1014–25. doi:10.1001/jama.2016.1203

70 Farinati F, Vitale A, Spolverato G, *et al.* Development and Validation of a New Prognostic System for Patients with Hepatocellular Carcinoma. *PLoS Med* 2016;**13**:1–18. doi:10.1371/journal.pmed.1002006

Assessment of study characteristics, used methods and specific reporting items

3 Background and Objectives	
3a: Type of predictive study	1. Prognostic<br>2. Diagnostic
3a: Topic Study	1. (Cardio)vascular<br>2. Oncological<br>3. Surgical<br>4. Other
3b: Model development and validation	1. Development<br>2. Validation<br>3. Development and Validation<br>4. Updating

4 Source of Data	
4a: Study design	1. RCT<br>2. Prospective cohort<br>3. Retrospective cohort<br>4. Nested case-control<br>5. Non-nested case-control

5 Participants	
5a: Study Setting	1. General population<br>2. Primary care<br>3. Secondary care

6 Outcome	
6a: Definition outcome	1. What is the outcome?<br>2. What is the time frame?
6a: Type of outcome	1. Dichotomous,<br>2. Continuous<br>3. Ordinal<br>4. Nominal<br>5. Time to event

9 Missing Data	
9 Type	1. MAR<br>2. MCAR<br>3. MNAR
9 Reason	Reason missing data
9 Handling	1. Complete-case analysis<br>2. Single imputation<br>3. Multiple imputation

	4. Missing indicator method<br>5. Last observation carried forward<br>6. Mean imputation<br>7. Other

10 Statistical Analysis and Methods	
10a: Analysis of predictors	1. Categorical<br>2. Linear: kept linear<br>3. Linear: dichotomized<br>4. Linear: categorized<br>5. Linear: polynomial transformation<br>6. Linear: spline transformation,<br>7. Linear: interaction<br>8. Linear: log-transformation<br>9. Other<br>(multiple options possible)
10b: Type of Model	1. Linear<br>2. Logistic<br>3. Ordinal<br>4. Nominal<br>5. Survival<br>6. Poisson<br>7. Points<br>8. Other
10b: Selection Predictors	1. A priori knowledge<br>2. Based on the literature<br>3. Statistically<br>(multiple options possible)
10b: Model building procedure	1. Entering all predictors<br>2. Forward selection<br>3. Backward selection<br>4. Best subset
10b: Statistical thresholds for model building	1. p-value: threshold<br>2. AIC/BIC<br>3. R^2<br>4. C-statistic/AUC<br>5. HR<br>6. Manually
10b: Internal Validation methods	1. Apparent<br>2. Random split<br>3. Cross-validation<br>4. Bootstrapping<br>(multiple options possible)

10d: External Validation methods	1. Temporal validation<br>2. Geographical validation<br>3. Fully independent<br>4. Other<br>(Multiple options possible)
10d: Performance Calibration	1. Test<br>2. Slope<br>3. Intercept and slope<br>4. Calibration in-the-large<br>5. Plot<br>(multiple options possible)
10d: Performance Discrimination	1. C-statistic / AUC after Development<br>2. C-statistic / AUC after Internal validation<br>3. C-statistic / AUC after external validation<br>4. D-statistic
10d: Performance Classification	1. Sensitivity / Specificity<br>2. Positive Predictive Value / Negative Predictive Value<br>3. Likelihood ratio<br>(multiple options possible)
10d: Performance Other	1. IDI<br>2. NRI<br>3. Decision curve analysis<br>(multiple options possible)
10d: Performance Overall	1. Brier<br>2. 2. R^2
10e: Model Updating	1. Intercept<br>2. all coefficients changed with same factor<br>3. Re-estimation all coefficients independently<br>4. 2+ selection of additional predictors<br>5. 3+ Reestimation all coefficients

13 Participants	
13a: Number of Participants	1. Number of participants with and without outcome
13b: Missing Values	Reported for<br>1. Any value<br>2. Per predictor<br>3. For predictors in general<br>4. For outcome<br>(multiple options possible)

Model Specification	
15a Intercept or baseline hazard	

15a: Coefficients	Coefficients reported:<br>1. All univariate<br>2. Univariate of included predictors in final model<br>3. All multivariate<br>4. Multivariate of predictors included in final model<br>(multiple option possible)
15a: Number of predictors	Number of predictors in final model
15a: Use of model	Use of model reported as:<br>1. Only regression coefficients<br>2. Intercept with regression coefficients<br>3. Simplified score (incl. nomogram or app)<br>(multiple options possible)
