# Supplementary material for: TRIPOD statement: a preliminary pre-post analysis of reporting and methods of prediction models
Source: BMJ Open. 2020 Sep 18;10(9):e041537. doi: 10.1136/bmjopen-2020-041537 (PMC7511612; doi:10.1136/bmjopen-2020-041537)
Supplement: Author's manuscript [file bmjopen-2020-041537.draft_revisions.pdf]

BMJ Open is committed to open peer review. As part of this commitment we make the peer review history of every article we publish publicly available.

When an article is published we post the peer reviewers' comments and the authors' responses online. We also post the versions of the paper that were used during peer review. These are the versions that the peer review comments apply to.

The versions of the paper that follow are the versions that were submitted during the peer review process. They are not the versions of record or the final published versions. They should not be cited or distributed as the published version of this manuscript.

BMJ Open is an open access journal and the full, final, typeset and author-corrected version of record of the manuscript is available on our site with no access controls, subscription charges or pay-per-view fees (http://bmjopen.bmj.com).

If you have any questions on BMJ Open's open peer review process please email info.bmjopen@bmj.com

BMJ Open

TRIPOD statement: a preliminary pre-post analysis of reporting and methods of prediction models

Journal:	*BMJ Open*
Manuscript ID	bmjopen-2020-041537
Article Type:	Original research
Date Submitted by the Author:	28-Jun-2020
Complete List of Authors:	Zamanipoor Najafabadi, Amir H.; Leiden University Medical Center<br>Ramspek, Chava; Leids Universitair Medisch Centrum, Clinical Epidemiology<br>Drekker, Friedo; Leiden University Medical Centre, Department of Clinical Epidemiology<br>Heus, Pauline; University Medical Center Utrecht, Utrecht University, Cochrane Netherlands, Julius Center for Health Sciences and Primary Care<br>Hooft, Lotty; University Medical Centre (UMC) Utrecht, University Utrecht (UU), Dutch Cochrane Centre (DCC), Julius Center for Health Sciences and Primary Care<br>Moons, Karel; Julius Center for Health Sciences and Primary Care, Epidemiology<br>Peul, Wilco; Leiden University Medical Center, Neurosurgery; Medical Center The Hague, Neurosurgery<br>Collins, Gary; Oxford University, NDORMS<br>Steyerberg, Ewout; Leiden University Medical Center, Department of Medical Statistics<br>van Diepen, Merel; Leiden University Medical Center, Clinical Epidemiology
Keywords:	STATISTICS & RESEARCH METHODS, EPIDEMIOLOGY, GENERAL MEDICINE (see Internal Medicine)

SCHOLARONE™
Manuscripts

I, the Submitting Author has the right to grant and does grant on behalf of all authors of the Work (as defined in the below author licence), an exclusive licence and/or a non-exclusive licence for contributions from authors who are: i) UK Crown employees; ii) where BMJ has agreed a CC-BY licence shall apply, and/or iii) in accordance with the terms applicable for US Federal Government officers or employees acting as part of their official duties; on a worldwide, perpetual, irrevocable, royalty-free basis to BMJ Publishing Group Ltd ("BMJ") its licensees and where the relevant Journal is co-owned by BMJ to the co-owners of the Journal, to publish the Work in this journal and any other BMJ products and to exploit all rights, as set out in our licence.

The Submitting Author accepts and understands that any supply made under these terms is made by BMJ to the Submitting Author unless you are acting as an employee on behalf of your employer or a postgraduate student of an affiliated institution which is paying any applicable article publishing charge ("APC") for Open Access articles. Where the Submitting Author wishes to make the Work available on an Open Access basis (and intends to pay the relevant APC), the terms of reuse of such Open Access shall be governed by a Creative Commons licence – details of these licences and which Creative Commons licence will apply to this Work are set out in our licence referred to above.

Other than as permitted in any relevant BMJ Author's Self Archiving Policies, I confirm this Work has not been accepted for publication elsewhere, is not being considered for publication elsewhere and does not duplicate material already published. I confirm all authors consent to publication of this Work and authorise the granting of this licence.

For peer review only

TRIPOD statement: a preliminary pre-post analysis of reporting and methods of prediction models

Amir H Zamanipoor Najafabadi BSc[1,2,3], Chava L Ramspek MD[3], Friedo Dekker[3], Pauline Heus MSc[4,5], Lotty Hooft PhD[4,5], Karel G M Moons MD PhD[4,5], Wilco C Peul MD PhD[1], Gary S Collins PhD[6,7], Ewout W Steyerberg PhD[8], Merel van Diepen PhD[3].

[1]*Department of Neurosurgery, University Neurosurgical Center Holland, Leiden University Medical Center & Haaglanden Medical Center & Haga Teaching Hospitals, Leiden & the Hageue, the Netherlands*
[2]*Department of Neurology, Leiden University Medical Center, Leiden, the Netherlands*
[3]*Department of Clinical Epidemiology, Leiden University Medical Center, Leiden, the Netherlands*
[4]*Cochrane Netherlands, University Medical Center Utrecht, Utrecht University*
[5]*Julius Center for Health Sciences and Primary Care, University Medical Center Utrecht, Utrecht University*
[6]*Centre for Statistics in Medicine, Nuffield Department of Orthopaedics, Rheumatology and Musculoskeletal Sciences, University of Oxford, Oxford, UK.*
[7]*National Institute for Health Research Oxford Biomedical Research Centre, John Radcliffe Hospital, Oxford, UK*
[8]*Department of Biomedical Data Sciences, Leiden University Medical Center, Leiden, The Netherlands.*

Corresponding author: Amir H. Zamanipoor Najafabadi, Department of Neurosurgery, Neurology and Clinical Epidemiology, Leiden University Medical Center, Leiden, the Netherlands. *Tel:* +31-(0)71-5262109. Fax: +31-(0)71-5266987. *Email:* *a.h.zamanipoor_najafabadi@lumc.nl*

**Type of article:**	Research
**Figures / Tables:**	3 figures and 2 Tables
**Word count main text:**	3550
**Word count abstract:**	320

For peer review only - http://bmjopen.bmj.com/site/about/guidelines.xhtml

ABSTRACT

Objectives

To assess the difference in completeness of reporting and methodological conduct of published prediction models before and after publication of the Transparent Reporting of a multivariable prediction model for Individual Prognosis Or Diagnosis (TRIPOD) Statement.

Methods

In the seven general medicine journals with the highest impact factor, we compared the completeness of the reporting and the quality of the methodology of prediction model studies published between 2012 and 2014 (pre-TRIPOD) with studies published between 2016 and 2017 (post-TRIPOD). For articles published in the post-TRIPOD period, we examined whether there was improved reporting for articles (1) citing the TRIPOD Statement, and (2) published in journals that published the TRIPOD Statement.

Results

A total of 70 articles were included (pre-TRIPOD: 32, post-TRIPOD: 38). No improvement was seen for the overall percentage of reported items after the publication of the TRIPOD Statement (pre-TRIPOD 74%, post-TRIPOD 76%, 95% CI of absolute difference: -4% to 7%). For the individual TRIPOD items, an improvement was seen for 16 (44%) items, while 3 (8%) items showed no improvement and 17 (47%) items showed a deterioration. Post-TRIPOD, there was no improved reporting for articles citing the TRIPOD statement, nor for articles published in journals that published the TRIPOD statement. The methodological quality improved in the post-TRIPOD period. More models were externally validated in the same article (absolute difference 8%, post-TRIPOD: 39%), used measures of calibration (21%, post-TRIPOD: 87%) and discrimination (9%, post-TRIPOD: 100%), and used multiple imputation for handling missing data (13%, post-TRIPOD: 50%).

Conclusions

Since the publication of the TRIPOD Statement, some reporting and methodological aspects have improved. Prediction models are still often poorly developed and validated and many aspects remain poorly reported, hindering optimal clinical application of these models. Long-term effects of the TRIPOD statement publication should be evaluated in future studies.

KEY WORDS: Diagnostic, Prediction, Prognostic, Reporting, TRIPOD, Methodology.

For peer review only - http://bmjopen.bmj.com/site/about/guidelines.xhtml

Strengths and limitations of this study

- This is the first study to assess the completeness of reporting and methodological conduct of prediction models published before and after publication of the TRIPOD statement.
- A limitation of this study is the short time period evaluated and therefore future studies are needed to assess the long-term effects on completeness of reporting and methodological conduct.
- Causality between publication of the TRIPOD statement and the found results cannot be established due to confounding.

For peer review only

For peer review only - http://bmjopen.bmj.com/site/about/guidelines.xhtml

INTRODUCTION

Prediction models cover both prognostic models, which aim to predict the risk of future outcomes, and diagnostic models, which aim to assess the presence or absence of a condition.[1] They provide information for differential diagnosis, additional testing and for patient selection on treatment. Interest in prediction models has sharply increased over the last two decades, translating to new methodological developments, especially regarding performance assessment of these models[2–4]. In addition, clinical guidelines are increasingly recommending the use of prediction models,[5,6] and consequently implementation of these models in clinical practice for individualised diagnostic and therapeutic decisions has surged.[7–10]

Previous systematic reviews on the quality of published prediction models have identified poor reporting and many methodological shortcomings in the development and validation of these models.[11–13] In response to these reviews, the Transparent Reporting of a multivariable prediction model for Individual Prognosis Or Diagnosis (TRIPOD) Statement was developed.[14] The TRIPOD Statement provides reporting recommendations for articles that describe the development and external validation of prediction models, aiming to enhance reporting transparency and hence interpretability, reproducibility, and clinical usability of these models.[14] Although the TRIPOD Statement primarily focuses on reporting and not on methods, current accepted methods for the development and validation of prediction models are discussed in the accompanied Explanation and Elaboration document.[15]

The primary aim of this study was to assess the difference in completeness of reporting and methodological conduct of published prediction models before and after publication in high impact general medicine journals.

For peer review only

METHODS

Systematic literature search

We selected the seven general medicine journals with the highest Web of Knowledge impact factor in 2017: *New England Journal of Medicine (NEMJ), Journal of the American Medical Association (JAMA), The Lancet, the British Medical Journal (The BMJ), Annals of Internal Medicine, PLOS Medicine, and BMC Medicine*. Articles on prediction models published in these journals before publication of the TRIPOD Statement (pre-TRIPOD: 01 January 2012 – 31 December 2014) and after publication of TRIPOD statement (post-TRIPOD: 01 January 2016 – 31 December 2017) were identified by a PubMed search string (Supplementary text 1). Articles published in 2015 were excluded from the search, as the TRIPOD Statement was published in 2015 and we regard this as a transition period. Titles and abstracts were screened by one reviewer (AHZN). Full-text articles were screened by two independent reviewers (AHZN and CLR) and disagreement was resolved by discussion and consensus with a senior author (MvD).

Article and model selection

Original articles with the primary aim of developing and/or validating multivariable models, both prognostic and diagnostic, were included. We excluded etiological studies, genetic marker studies, and model impact studies, as these are not covered by the TRIPOD Statement. Included articles were classified as 1) development, 2) development and external validation, 3) external validation, and 4) extension/updating of models. For articles addressing multiple models but not explicitly recommending a single model, the model with the most predictors was evaluated. For instance, Hippisley-Cox (2013) described model A, B and C for the prediction of future risk of cardiovascular disease, with model B being the same as model A with the addition of several predictors and interactions and model C being the same as model B with the addition of one variable. In this case model C was evaluated.

Assessment of adherence to TRIPOD criteria

In 2018, authors of the TRIPOD Statement published a TRIPOD adherence assessment form and adherence scoring rules, which were also used in our study. [16–18] The TRIPOD Adherence form is a measurement tool developed for authors who want to evaluate the adherence of prediction model studies to TRIPOD, e.g. over time or in a certain medical domain. In general, when multiple aspects were described within a TRIPOD item, all aspects needed to be reported to score a point for that item. For instance, the item title contains four sub-items (e.g. i. identifying the study as development and/or validation of a ii. prediction model with iii. description of target population and iv. outcome) and all four aspects need to be reported to score a point for this specific item. For all items and aspects of the checklist it was assessed whether it was reported in the main article or supplementary materials. The main analyses were based on items reported in either the main text or supplements. Each article was only assessed for items applicable to the study (i.e. development and/or external validation, or incremental value study). Scores for reporting level were calculated by assigning a single point for each reported item applicable to the study and total reporting level scores were converted to percentages based on the maximum possible score, and followed published scoring rules for the TRIPOD Adherence form.[16,17]

For peer review only - http://bmjopen.bmj.com/site/about/guidelines.xhtml

Assessment of study characteristics and used methods

In addition to the completeness of reporting following the TRIPOD statement, we assessed specific study characteristics and methods used in the included articles. To this end, we developed a comprehensive data extraction form based on previous studies, current methodological consensus, and the TRIPOD Exploration and Elaboration document (Supplementary text 1).[11,13,15,19–22] In summary the following topics were assessed: general study characteristics (i.e. diagnostic-prognostic and study topic), handling of missing data, model development methods, type of external validation and updating, and performance measures. To facilitate interpretation of the results section, main recommendations of the TRIPOD Exploration and Elaboration document are presented in Table 1. Assessment of these items was performed by two independent reviewers (AHZN and CLR) and a senior author (MvD) where necessary. In addition, for all articles published in the post-TRIPOD period, we extracted whether authors cited or referred to the TRIPOD Statement, provided the completed checklist, if the article was published in a journal that published the Statement (*The BMJ*, *Annals of Internal Medicine*, *BMC Medicine*), or was published in a journal that clearly stated in the author guidelines that they required TRIPOD adherence for submitted work at the time of writing this manuscript (*The BMJ, JAMA, and PLOS Medicine*). While all included journals (except for the *NEJM*) encouraged authors to follow the Equator Network guidelines, which includes the TRIPOD checklist, in their author instructions, only *The BMJ, JAMA and PLOS Medicine* required adherence to the Equator network guidelines and also required to include a filled-out checklist at the time of submission.

Analysis and reporting of results

Reporting levels are presented as percentages, stratified by journal, and for comparison the absolute difference in percentages with 95% confidence intervals are reported. Analyses were performed with IBM SPSS statistics (version 23.0, Armonk, NY, USA). Main results of the completeness of both reporting and methods are reported in text and detailed results are reported in the (Supplementary) Tables. Comparisons were made between articles I) Pre- and Post-TRIPOD, II) post-TRIPOD between articles published in journals that published and did not publish the TRIPOD, III) between articles published in journals that require TRIPOD adherence or not, IV) citing vs. not citing the TRIPOD, and V) providing vs. not providing a completed TRIPOD checklist. Furthermore, to estimate changes over time regardless of the TRIPOD statement, a comparison was made between pre-TRIPOD articles and post-TRIPOD articles not citing the TRIPOD.

For peer review only

For peer review only - http://bmjopen.bmj.com/site/about/guidelines.xhtml

RESULTS

Characteristics of included studies

The PubMed search string retrieved 481 articles, of which the full-text of 119 were read and 70 met our inclusion criteria (pre-TRIPOD: 32 articles, post-TRIPOD: 38 articles, Figure 1, Supplementary Text 1). Most of the included articles were published in *The BMJ* (n=38), and least in *The Lancet* (n=3) and NEJM (n=1). In both the pre- and post-TRIPOD period the majority of articles described prognostic models (as opposed to diagnostic models) and this increased in the post-TRIPOD period (pre-TRIPOD: 59%, post-TRIPOD: 89%) (Table 2). In the post-TRIPOD period the percentage of articles describing both the development and validation of a model (pre-TRIPOD: 31%, post-TRIPOD: 39%) or solely the external validation (pre-TRIPOD: 13%, post-TRIPOD: 26%) increased too. Thirty-two percent of articles only described the development of a prediction model without external validation in the post-TRIPOD period, compared to 44% in the pre-TRIPOD period. The majority of models were developed and/or validated using data from observational cohorts (pre-TRIPOD: 81%, post-TRIPOD: 82%) compared to other study designs such as randomised trials. More than half of the articles published in the post-TRIPOD period referred to the TRIPOD Statement (n=20, 53%) and were published in journals that published the TRIPOD Statement (n=21, 55%). The TRIPOD Statement was cited in 48% of articles published in journals that published the TRIPOD, and in 59% of articles in journals that did not publish TRIPOD.

Assessment of adherence to TRIPOD statement

Using the 2018 TRIPOD Adherence assessment form, a minimal non-significant increase in the overall percentage of reported items was found comparing the pre-TRIPOD period (74%) with the post-TRIPOD period (76%, absolute difference 2%, 95% CI: -4% to 7%, Figure 2, Supplementary Table 1), with no clear trend over the years (Supplementary Figure 1). Results were similar for the comparison between pre-TRIPOD articles and post-TRIPOD articles not citing the statement (76%, absolute difference 2%, 95%CI: -5% to 9%). An improvement for 16 of the individual TRIPOD items (44% of items, Supplementary Table 2) was seen, while 3 (8%) of items showed no improvement and 17 (47%) items showed a decrease in the percentage of articles appropriately reporting the item. Post-TRIPOD, for articles referring vs. not referring to the statement, published in journals that published vs. did not publish the statement, and published in journals that required adherence to the statement vs. did not require adherence to the statement, no difference in the completeness of reporting was observed (Supplementary Tables 3-5). Five articles presented the completed TRIPOD checklist in the supplementary material and the overall percentage of reporting for these articles was 80%. The percentage of articles reporting TRIPOD items in their supplement is presented in Supplementary Table 6.

Assessment of specific TRIPOD items

Abstract

In both the pre-TRIPOD (16%) and post-TRIPOD period (8%), most abstracts did not report all the proposed sub-items (TRIPOD item 2).

Reporting of missing data

In general, the reporting of missing data (TRIPOD item 13b) improved from 59% in the pre-TRIPOD period to

For peer review only - http://bmjopen.bmj.com/site/about/guidelines.xhtml

71% in the post-TRIPOD period, though fewer studies reported missingness per predictor in the post-TRIPOD period (pre-TRIPOD: 53%, post-TRIPOD: 37%, Figure 3 and Supplementary Table 7). Most studies did not report the reason for missing data (pre-TRIPOD: 84%, post-TRIPOD: 95%).

Model development and presentation

In the post-TRIPOD period, proper description of the characteristics of study participants (TRIPOD item 13b) was less often reported (37%) than in the pre-TRIPOD period (50%). In the post-TRIPOD period, method of predictor selection (TRIPOD-item 10b) was more often reported (70%) than in the pre-TRIPOD period (62%), as was internal validation (TRIPOD-item 10b) of the developed model (pre-TRIPOD 62%, post-TRIPOD 74%). If performed, unadjusted analyses were less often reported (TRIPOD item 14b) in the post-TRIPOD period (64%) than in the pre-TRIPOD period (86%). In the post-TRIPOD period, the full model (i.e. intercept or baseline hazard and all regression coefficients: TRIPOD-item 15a) was presented more frequently (41%), compared with the pre-TRIPOD period (29%). However, in both eras some studies still reported no information at all on the final model (pre-TRIPOD 8%; post-TRIPOD 4%, Figure 3 and Supplementary Table 8). To improve clinical usability (TRIPOD-item 15b), more than one third of studies reported to have developed a web application (pre-TRIPOD: 38%, post-TRIPOD: 37%) and some studies provided a simplified clinical risk score or nomogram (pre-TRIPOD: 29%; post-TRIPOD: 26%).

Performance measures

The percentage of studies reporting calibration (TRIPOD-item 16) of the model increased from 66% in the pre-TRIPOD period to 87% in the post-TRIPOD period. Discrimination (TRIPOD-item 16), was reported by all studies in the post-TRIPOD period and by 91% of studies in the pre-TRIPOD period. Measures of classification were reported less frequently in the post-TRIPOD period (pre-TRIPOD: 69%, post-TRIPOD: 58%). Measures of clinical usefulness like decision curve analysis were only reported by 2 (6%) studies in the pre-TRIPOD period and 7 (21%) studies in the post-TRIPOD period. Measures of overall performance like the Brier score or R^2 were infrequently reported in both periods (pre-TRIPOD: 19%, post-TRIPOD: 21%). Detailed results are depicted in Supplementary Table 7.

Assessment of methods

Handling of missing data

Multiple imputation was the most frequently performed approach for handling missing data (pre-TRIPOD: 38%, post-TRIPOD: 50%). The number of studies that used a complete case analysis remained constant and was 16% in both the pre- and post-TRIPOD period.

Model development

Post-TRIPOD, the number of studies that included predictors based on significance levels in univariable analysis decreased (pre-TRIPOD: 67%, post-TRIPOD: 44%, Figure 2 and Supplementary Table 8) as well as the number of studies using stepwise methods to retain predictors (pre-TRIPOD: 63%, post-TRIPOD: 48%). In general, a larger number of candidate predictors was used in the post-TRIPOD period (median: 25), compared with pre-TRIPOD period (median: 20). Internal validation was more frequently performed in the post-TRIPOD period (74%) compared with the pre-TRIPOD period (62%). When internal validation was performed,

For peer review only - http://bmjopen.bmj.com/site/about/guidelines.xhtml

bootstrapping was the most frequently used method in both time periods with an increase from 29% in the pre-TRIPOD period to 41% in the post-TRIPOD period.

Performance measures

The majority of studies presented measures of calibration (pre-TRIPOD: 66%, post-TRIPOD: 87%) and discrimination (pre-TRIPOD: 91%, post-TRIPOD: 100%, Figure 3 and Supplementary Table 7).A calibration plot and this increased in the post-TRIPOD period (pre-TRIPOD: 50%, post-TRIPOD: 82%)). Discrimination was primarily assessed with the C-statistic and Area Under the Curve (AUC) methods (pre-TRIPOD: 91%, post-TRIPOD: 100%). Measures of classification were reported in more than half of the studies (pre-TRIPOD: 69%, post-TRIPOD: 58%),mostly assessed with diagnostic test summary statistics (i.e. sensitivity, specificity and positive and negative predictive values) (pre-TRIPOD: 63%, post-TRIPOD: 50%) and to a lesser extent the integrated discrimination improvement (IDI; pre-TRIPOD: 16%, post-TIRPOD: 11%) or the net reclassification improvement (NRI; pre-TRIPOD 25%, post-TRIPOD: 18%).

External validation and model updating

Most external validation studies performed the validation in individuals fully unrelated to the development cohort (pre-TRIPOD 78%, post-TRIPOD: 88%, Figure 3 and Supplementary Table 9). Models were updated with an additional predictor in 4 (13%) studies before the TRIPOD statement and in 1 (3%) study after the TRIPOD statement.

For peer review only

For peer review only - http://bmjopen.bmj.com/site/about/guidelines.xhtml

DISCUSSION

No significant improvement in the overall reporting quality of prediction models published in the seven general medicine journals with the highest impact factor was found in the post-TRIPOD period, according to the TRIPOD Adherence form. However, an improvement in general methodological conduct was found. Notably, more studies described external validation of a model, reported information on missing data, used multiple imputation methods instead of complete case analysis for handling of missing data, selected and maintained variables in multivariable models based on clinical relevance instead of statistical cut-offs, and assessed both discrimination and calibration measures. While improvement was found for almost half of the TRIPOD items, no improvement or a deterioration was found for the other half of the items.

Recommendations on reporting and methods

Though improvements over time in specific aspects of reporting and methods were apparent, there is room for further progress. While an increase in studies reporting the percentage of missing data in the post-TRIPOD period was observed, the amount of missingness was often not reported per predictor, yet this is important for the assessment of clinical usability of the model.[15] Multiple imputation was the most frequently performed method for handling missing data, which generally is the preferred approach.[23] Reporting of all coefficients of the final multivariable model and intercept, which is necessary for external validation and clinical use of models, increased over time.[22] Although widely discouraged, a number of studies in both the pre-TRIPOD and post-TRIPOD period included predictors in multivariable prediction models based on data-driven selection methods such as univariable significance and/or stepwise methods. Such methods increase the risk of overfitted and poorly calibrated models.[11,22–26] Instead, it is advised to select predictors based on clinical knowledge and previous literature.[27] While the percentage of studies that both developed and externally validated a model increased over time, still more than 30% of articles only described the development of a model. External validation in a fully independent cohort is strongly recommended, as model performance might significantly decrease in cohorts other than the development cohort.[28] Assessment of both calibration and discrimination also increased, which is necessary in order to judge a model's predictive accuracy. Calibration refers to the agreement between absolute predicted and observed outcomes and the majority of studies used the preferred calibration plot.[29] Discrimination, a relative measure on the ability to distinguish between patients with and without the outcome, was reported by almost all studies.[29]

Comparison with other reporting guidelines

A large number of reporting guidelines have been published for various study types.[19,30–33] Mixed results on the effect of these guidelines on the completeness of reporting have been found.[34–38] While an overall modest improvement in reporting was described for randomised controlled trials after publication of the CONSORT statement and by the STARD statement for diagnostic studies, no clear improvement was described for observational studies by the STROBE statement and prognostic marker studies by the REMARK guideline as described by the authors of these studies.[34–38] These findings pose the question how the introduction and publication of these guidelines can optimally impact the research field. For both the CONSORT and STARD statement, journals endorsing the statement showed a higher level of reporting compared with journals not endorsing these statements. Nevertheless, this was not found for the REMARK guideline, nor in our study for

For peer review only - http://bmjopen.bmj.com/site/about/guidelines.xhtml

the TRIPOD statement.[34,37,38] Evidence of a relation between citing the statement and reporting level is also limited, as no association between this was found for the STARD nor in our study.[38] Requiring authors to provide and publish the completed checklist might help to improve reporting levels, as we found that the small numbers of studies providing the checklist reported more items on average. Therefore, we do not only recommend journals to ask authors to submit the completed checklist upon submission, but also require authors to publish it as a supplement, and reviewers and editors to control the provided checklist. However, as endorsing, citing and providing the checklist seems to have only a small effect on the reporting quality, we believe it is even more important to train methodologists and clinicians to interpret and use the checklist. This is supported by the results that even studies that provided the completed checklist, still did not report all items of the TRIPOD statement in analysis of reporting. Documents such as the TRIPOD Exploration and Elaboration document facilitate proper interpretation, but we believe that the threshold to use this detailed document might be too high for the unexperienced researcher. Other possibilities to familiarize authors with the checklist should be explored, such as collaborative efforts of educational institutions and the TRIPOD committee to train researchers and clinicians. Online training courses might be of added value to reach a large target group.

Comparison with other reviews on the completeness of reporting and methodological conduct of prediction models

Previous studies, published between 2012 and 2014, concluded poor reporting and use of methods for prediction models.[11,13,20,21] Comparing our results with a study assessing reporting and methods of prediction studies published in 6 high impact general medicine journals in 2008, improvement since then is clear for both methods and reporting. Considering methods more studies are externally validated, compose calibration plots to assess calibration and use multiple imputation for handling missing data. Improvement in reporting is also apparent as more studies report calibration and discrimination measures. Furthermore, a recently published article assessed the reporting quality of prediction models published in 37 clinical domains in 2014 using the 2018 TRIPOD Adherence assessment form, which found similar results to our Pre-TRIPOD results.[17] As we only included articles published in high impact general medicine journals it is difficult to generalize these results to the entire medical academic research field. We could argue that the improvement we observed might be an overestimation if general medical journals adopted the TRIPOD guidelines and new methodological insights with more speed and rigour. However, the opposite might also be true as these high impact general medicine journals already had high methodological standards before the TRIPOD statement publication.[11,13,16,21,35,36,39,40]

Strengths and Limitations

A limitation of the current study is that the evaluation of studies was limited to the first two years after the TRIPOD statement publication. It may take some years before a reporting guideline is widely disseminated and accepted and the full impact is measurable. However, to somewhat overcome this problem we did not include any articles published in 2015, as the TRIPOD statement was published in January 2015 and we therefore saw this as a transition period. In addition, a previously published study on the effect of STARD found significant improvement within two years after publication.[38] Furthermore it is not possible to causally attribute the reported changes to the TRIPOD statement, as the results might be confounded by other developments in the last decade, such as publication of multiple series on the conduct of prediction models, publication of other

For peer review only - http://bmjopen.bmj.com/site/about/guidelines.xhtml

guidelines such as the REMARK guideline for tumor marker prognostic studies, and a general increase in the numbers of published prediction models.[41–43] One may also expect that authors who work in the field of prediction models are aware of the publication of the TRIPOD statement, especially those who publish in high impact general medicine journals. A strength of the study is that the actual used methods for the development, description, validation and updating of prediction models were also assessed. While reporting and used methods are inherently related, the focus is different. A poorly developed model may be described fully and transparently in a manuscript and score high on reporting quality and vice versa a well-developed model may have poor reporting.[16] Furthermore, we have facilitated comparison to future TRIPOD reviews by using the 2018 TRIPOD Adherence assessment form. Although both reporting and methods were comprehensively assessed, we might have missed interesting items for evaluation, especially as the field of prediction models is continuously developing. We also did not assess the risk of bias of the included studies with the PROBAST risk of bias assessment tool, as it would be not feasible to score the included articles according to the PROBAST, since to do so subject-specific knowledge is required and the included studies span a wide range of clinical subjects. Furthermore, as the PROBAST only gives suggestions for signalling questions and no scoring rules, it does not completely fit with the aim to assess the actual used methods of the included studies. Furthermore, it would have been of interest to compare articles published in journals that between January 2016 and December 2017 obligated authors to complete the TRIPOD checklist, however this information was not available.

Unanswered questions and future research

Future studies should focus on the long-term effects of the TRIPOD statement publication on reporting quality and methods, using the 2018 TRIPOD Adherence form to allow for comparisons over time using the same adherence assessment tool. In addition, effects of the statement should be assessed in different medical fields for which a pre-TRIPOD baseline measurement is already performed.[16] Earlier studies on the effect of other reporting guidelines showed that the effect of these guidelines may be smaller or larger in specific medical fields.[34,40]

A new emerging field is the development of prediction models using artificial intelligence, machine learning and deep learning methods. In addition, more often omics data is used as predictors for these models.[44] While these models have many similarities with traditional regression methods, they differ in some aspects and may require specific guidelines on reporting.[44,45] Accordingly, the TRIPOD-AI tool has recently been announced and is underway.[46] Similarly, reporting guidelines for prediction model impact studies are missing.

With the increasing number of reporting guidelines and lack of clear evidence that all guidelines improve reporting quality, research should be conducted to find methods to optimise the form, use and impact of these guidelines. With this in mind, there should also be focus on the overlap between different reporting guidelines. Prediction models can be reported following the TRIPOD statement, the STARD statement for diagnostic test accuracy studies, and REMARK for prognostic tumor marker studies. As an increasing amount of studies contain multiple goals, analyses and data sources, it may be difficult to adhere to all applicable and relevant guidelines within the maximum word count. This holds especially for the abstract section of articles.

Conclusion

For peer review only - http://bmjopen.bmj.com/site/about/guidelines.xhtml

No improvement was found comparing the post-TRIPOD period with the pre-TRIPOD period in the overall reporting quality of prediction models published in the seven general medicine journals with the highest impact factor. Comparison of articles published before the TRIPOD statement with non-TRIPOD citing articles published after the TRIPOD statement, yielded similar results as the main pre-post comparison, further suggesting a lack of direct impact of the TRIPOD statement on overall reporting levels. However improvement was found in various specific aspects methodological conduct. More studies described external model validations, reported information on missing data, used multiple imputation methods for handling of missing data, reported the full prediction model and reported information on performance measures. However, there is still room for improvement in both the reporting and used methods of these models, as prediction models are still erroneously developed and validated and many aspects remain poorly reported, hindering optimal use of these models in clinical decision making. Long-term effects of the TRIPOD statement publication should be evaluated in future studies, ideally using the same 2018 TRIPOD Adherence assessment form to allow for comparisons over time.

For peer review only

Contributorship: AHZN conceived the study and AHZN, MvD, CLR, and EWS developed the study design. AHZN and CLR screened the literature and performed the data-extraction. AHZN performed the statistical analysis and wrote the first and successive drafts of the manuscript. All authors interpreted the data and critically revised the manuscript.

Funding: AHZN and CLR were supported by personal Leiden University Medical Center MD/PhD Scholarships. MvD was supported by a grant from the Dutch Kidney Foundation (16OKG12). GSC is supported by the NIHR Biomedical Research Centre, Oxford and Cancer Research UK programme grant (C49297/A29084).

Competing interests: GSC, KGMM, and EWS are members of the TRIPOD group. All authors have completed the ICMJE uniform disclosure form at http://www.icmje.org/coi_disclosure.pdf (available on request from the corresponding author) and declare no other support from any organisation for the submitted work than the grants reported in the funding section; no financial relationships with any organisations that might have an interest in the submitted work in the previous three years, no other relationships or activities that could appear to have influenced the submitted work.

Ethical approval: Not required

Data sharing: All datasets that were used are retrievable following the instruction of the original papers.

Transparency: The lead author (AHZ) affirms that this manuscript is an honest, accurate, and transparent account of the study being reported; that no important aspects of the study have been omitted; and that any discrepancies from the study as planned have been explained.

Patient and Public Involvement: It was not appropriate or possible to involve patients or the public in the design, or conduct, or reporting, or dissemination plans of our research.

Dissemination declaration: not applicable.

For peer review only

For peer review only - http://bmjopen.bmj.com/site/about/guidelines.xhtml

REFERENCES

1 Moons KGM, Royston P, Vergouwe Y, *et al.* Prognosis and prognostic research: what, why, and how? *Bmj* 2009;**338**:b375–b375. doi:10.1136/bmj.b375

2 Steyerberg EW, Vickers AJ, Cook NR, *et al.* Assessing the performance of prediction models : A framework for some traditional and novel measures. *Epidemiology* 2010;**21**:128–38. doi:10.1097/EDE.0b013e3181c30fb2.Assessing

3 Pencina MJ, Demler O V. Novel metrics for evaluating improvement in discrimination: net reclassification and integrated discrimination improvement for normal variables and nested models. *Stat Methods Med Res* 2012;**31**:101–13. doi:10.1002/sim.4348.Novel

4 Pencina MJ, Fine JP, D'Agostino RB. Discrimination slope and integrated discrimination improvement - properties, relationships and impact of calibration. *Stat Med* Published Online First: 2016. doi:10.1002/sim.7139

5 Camm AJ, Kirchhof P, Lip GYH, *et al.* Guidelines for the management of atrial fibrillation. *Eur Heart J* 2010;**31**:2369–429. doi:10.1093/eurheartj/ehq278

6 Ravdin PM, Siminoff L a, Davis GJ, *et al.* Computer program to assist in making decisions about adjuvant therapy for women with early breast cancer. *J Clin Oncol* 2001;**19**:980–91. doi:10.1200/jco.2001.19.4.980

7 Stiell IG, Greenberg GH, McKnight RD, *et al.* A study to develop clinical decision rules for the use of radiography in acute ankle injuries. *Ann Emerg Med* 1992;**21**:384–90. doi:10.1016/S0196-0644(05)82656-3

8 Tesdale G, Jennet B. Assessment of coma and impaired consciousness. *Lancet* 1974;**13**:81–4.

9 Apgar V. Proposal for new method of evaluation of newborn infant. *Anesth Analg* 1953;**32**:260–7.

10 Wilson PWF, D'Agostino RB, Levy D, *et al.* Prediction of Coronary Heart Disease Using Risk Factor Categories. *Circulation* 1998;**97**:1837–47. doi:10.1161/01.CIR.97.18.1837

11 Bouwmeester W, Zuithoff NPA, Mallett S, *et al.* Reporting and methods in clinical prediction research: A systematic review. *PLoS Med* 2012;**9**:e1001221. doi:10.1371/journal.pmed.1001221

12 Mallett S, Royston P, Waters R, *et al.* Reporting performance of prognostic models in cancer: a review. *BMC Med* 2010;**8**:21. doi:1741-7015-8-21 [pii] 10.1186/1741-7015-8-21

13 Collins GS, Groot JA De, Dutton S, *et al.* External validation of multivariable prediction models : a systematic review of methodological conduct and reporting. *BMC Med Res Methodol* 2014;**14**:1–11.

14 Collins GS, Reitsma JB, Altman DG, *et al.* Transparent reporting of a multivariable prediction model for individual prognosis or diagnosis (TRIPOD): The TRIPOD Statement. *BMJ* 2014;**67**:1142–51. doi:10.1016/j.eururo.2014.11.025

15 Moons KGM, Altman DG, Reitsma JB, *et al.* Transparent Reporting of a multivariable prediction model for Individual Prognosis Or Diagnosis (TRIPOD): Explanation and Elaboration. *Ann Intern Med* 2015;**162**:W1–73. doi:10.7326/M14-0698

16 Heus P, Damen JAAG, Pajouheshnia R, *et al.* Poor reporting of multivariable prediction model studies: towards a targeted implementation strategy of the TRIPOD statement. *BMC Med* 2018;**16**:120. doi:10.1186/s12916-018-1099-2

For peer review only - http://bmjopen.bmj.com/site/about/guidelines.xhtml

17 Heus P, Damen JAAG, Pajouheshnia R, *et al.* Uniformity in measuring adherence to reporting guidelines: The example of TRIPOD for assessing completeness of reporting of prediction model studies. *BMJ Open* 2019;**9**:1–6. doi:10.1136/bmjopen-2018-025611

18 TRIPOD Downloads. https://www.tripod-statement.org/Downloads#Checklist (accessed 11 Feb2020).

19 Collins GS, Reitsma JB, Altman DG, *et al.* Transparent reporting of a multivariable prediction model for individual prognosis or diagnosis (TRIPOD): The TRIPOD Statement. *BMC Med* 2015;**13**:1–10. doi:10.1016/j.eururo.2014.11.025

20 Collins GS, Omar O, Shanyinde M, *et al.* A systematic review finds prediction models for chronic kidney disease were poorly reported and often developed using inappropriate methods. *J Clin Epidemiol* 2013;**66**:268–77. doi:10.1016/j.jclinepi.2012.06.020

21 Collins GS, Mallett S, Omar O, *et al.* Developing risk prediction models for type 2 diabetes : a systematic review of methodology and reporting. *BMC Med* 2011;**9**:1–14.

22 Steyerberg EW. *Clinical prediction models: a practical approach to development, validation, and updating*. New York, NY, US: : Springer 2009.

23 Jr FEH, Lee KL, Mark DB. Tutorial in Biostatistics Multivariable Prognostic Models : Issues in Developing Models , Evaluating Assumptions and Adequacy , and Measuring and Reducing Errors. *Stat Med* 1996;**15**:361–87. doi:10.1002/(SICI)1097-0258(19960229)15:4<361::AID-SIM168>3.0.CO;2-4

24 Sun GW, Shook TL, Kay GL. Inappropriate use of bivariable analysis to screen risk factors for use in multivariable analysis. *J Clin Epidemiol* 1996;**49**:907–16. doi:10.1016/0895-4356(96)00025-X

25 Steyerberg EW, Harrell FE, Borsboom GJJ., *et al.* Internal validation of predictive models. *J Clin Epidemiol* 2001;**54**:774–81. doi:10.1016/S0895-4356(01)00341-9

26 Moons KGM, Kengne AP, Woodward M, *et al.* Risk prediction models: I. Development, internal validation, and assessing the incremental value of a new (bio)marker. *Heart* 2012;**98**:683–90. doi:10.1136/heartjnl-2011-301246

27 Steyerberg EW, Vergouwe Y. Towards better clinical prediction models: Seven steps for development and an ABCD for validation. *Eur Heart J* 2014;**35**:1925–31. doi:10.1093/eurheartj/ehu207

28 Moons KGM, Kengne AP, Grobbee DE, *et al.* Risk prediction models: II. External validation, model updating, and impact assessment. *Heart* 2012;**98**:691–8. doi:10.1136/heartjnl-2011-301247

29 Altman DG, Vergouwe Y, Royston P, *et al.* Prognosis and prognostic research: validating a prognostic model. *Bmj* 2009;**338**:b605–b605. doi:10.1136/bmj.b605

30 Von Elm E, Altman DG, Egger M, *et al.* The Strengthening the Reporting of Observational Studies in Epidemiology (STROBE) statement: Guidelines for reporting observational studies. *PLoS Med* 2007;**4**:1623–7. doi:10.1371/journal.pmed.0040296

31 Schulz KF, Altman DG, Moher D, *et al.* CONSORT 2010 Statement: updated guidelines for reporting parallel group randomised trials. *BMC Med* 2010;**8**:18. doi:10.1186/1741-7015-8-18

32 Bossuyt PM, Reitsma JB, Bruns DE, *et al.* The STARD statement for reporting studies of diagnostic accuracy: Explanation and elaboration. *Clin Chem* 2003;**49**:7–18. doi:10.1373/49.1.7

33 McShane LM, Altman DG, Sauerbrei W, *et al.* REporting recommendations for tumor MARKer prognostic studies (REMARK). *Breast Cancer Res Treat* 2006;**100**:229–35. doi:10.1007/s10549-006-9242-8

For peer review only

For peer review only - http://bmjopen.bmj.com/site/about/guidelines.xhtml

34 Moher D, Jones a, Lepage L. Use of the CONSORT statement and quality of reports of randomized trials: a comparative before-and-after evaluation. *JAMA* 2001;**285**:1992–5. doi:10.1001/jama.285.15.1992

35 Bastuji-Garin S, Sbidian E, Gaudy-Marqueste C, *et al.* Impact of STROBE Statement Publication on Quality of Observational Study Reporting: Interrupted Time Series versus Before-After Analysis. *PLoS One* 2013;**8**:2–9. doi:10.1371/journal.pone.0064733

36 Poorolajal J, Cheraghi Z, Irani AD, *et al.* Quality of Cohort Studies Reporting Post the Strengthening the Reporting of Observational Studies in Epidemiology (STROBE) Statement. *Epidemiol Health* 2011;**33**:e2011005. doi:10.4178/epih/e2011005

37 Sekula P, Mallett S, Altman DG, *et al.* Did the reporting of prognostic studies of tumour markers improve since the introduction of REMARK guideline ? A comparison of reporting in published articles. *PLoS One* 2017;:1–15.

38 Smidt N, Rutjes AWSWS, van der Windt DAWMWM, *et al.* The quality of diagnostic accuracy studies since the STARD statement. *Neurology* 2006;**67**. doi:10.1212/01.wnl.0000238386.41398.30

39 Moher D, Jones A, Lepage L, *et al.* Use of the CONSORT Statement and Quality of Reports of Randomized Trials. *Jama* 2001;**285**:1992. doi:10.1001/jama.285.15.1992

40 Kiehna EN, Starke RM, Pouratian N, *et al.* Standards for reporting randomized controlled trials in neurosurgery. *J Neurosurg* 2011;**114**:280–5. doi:10.3171/2010.8.JNS091770

41 Hemingway H, Croft P, Perel P, *et al.* Prognosis research strategy (PROGRESS) 1: A framework for researching clinical outcomes. *BMJ* 2013;**346**:1–11. doi:10.1136/bmj.e5595

42 Moons KGM, Altman DG, Vergouwe Y, *et al.* Prognosis and prognostic research: application and impact of prognostic models in clinical practice. *BMJ* 2009;**338**:b606.

43 Altman DG, McShane LM, Sauerbrei W, *et al.* Reporting recommendations for tumor marker prognostic studies (REMARK): Explanation and elaboration. *PLoS Med* 2012;**9**. doi:10.1371/journal.pmed.1001216

44 Shameer K, Johnson KW, Glicksberg BS, *et al.* Machine learning in cardiovascular medicine: are we there yet? *Heart* 2018;:heartjnl-2017-311198. doi:10.1136/heartjnl-2017-311198

45 Shameer K, Johnson KW, Glicksberg BS, *et al.* The whole is greater than the sum of its parts: combining classical statistical and machine intelligence methods in medicine. *Heart* 2018;**104**:1228–1228. doi:10.1136/heartjnl-2018-313377

46 Collins GS, Moons KGM. Reporting of artificial intelligence prediction models. *Lancet* 2019;**393**:1577–9. doi:10.1016/S0140-6736(19)30037-6

47 Janssen KJM, Donders ART, Harrell FE, *et al.* Missing covariate data in medical research: To impute is better than to ignore. *J Clin Epidemiol* 2010;**63**:721–7. doi:10.1016/j.jclinepi.2009.12.008

48 Sterne JAC, White IR, Carlin JB, *et al.* Multiple imputation for missing data in epidemiological and clinical research: potential and pitfalls. *BMJ* 2009;**338**. doi:10.1136/bmj.b2393

49 Donders ART, van der Heijden GJMG, Stijnen T, *et al.* Review: A gentle introduction to imputation of missing values. *J Clin Epidemiol* 2006;**59**:1087–91. doi:10.1016/j.jclinepi.2006.01.014

50 Steyerberg EW, Vickers AJ, Cook NR, *et al.* Assessing the Performance of Prediction Models. *Epidemiology* 2010;**21**:128–38. doi:10.1097/EDE.0b013e3181c30fb2

For peer review only

For peer review only - http://bmjopen.bmj.com/site/about/guidelines.xhtml

51 Debray TPA, Vergouwe Y, Koffijberg H, *et al.* A new framework to enhance the interpretation of external validation studies of clinical prediction models. *J Clin Epidemiol* 2015;**68**:279–89. doi:10.1016/j.jclinepi.2014.06.018

For peer review only

For peer review only - http://bmjopen.bmj.com/site/about/guidelines.xhtml

FIGURE CAPTIONS

Figure 1: Flow chart of search results and selection procedure

Figure 2: TRIPOD reporting scores

Figure 3: Comparison of used methods in the pre-TRIPOD and post-TRIPOD period

For peer review only

For peer review only - http://bmjopen.bmj.com/site/about/guidelines.xhtml

Table 1: Recommended methods and analyses for the development and validation of prediction models including supportive references

Methodology		
**Handling of missing data**	It is generally advised to use multiple imputation for handling of missing data. Complete case analysis, single or mean imputation are inefficient methods to estimate coefficients	[47–49]
**Selection and retaining of predictors in multivariable models**	Predictor selection and retaining is preferably based on clinical knowledge and previous literature, instead of significance levels in univariable or stepwise analysis.	[22,26,27]
**Internal validation**	It is advised to internally validate the model to assess optimism in performance and reduce over-fitting. An efficient method is bootstrapping; split-sample validation should be avoided.	[25,26]
**Calibration**	It is advised to assess the calibration of a model at external validation. The preferred method is a calibration plot, with intercept and slope, and not statistical tests (e.g. Hosmer-Lemeshow), as a plot retains the most information on possible miscalibration.	[22,26,27,50]
**External validation**	External validation of models is needed for rigorous assessment of performance. The preferred external validation population is fully independent.	[28,51]

For peer review only

For peer review only - http://bmjopen.bmj.com/site/about/guidelines.xhtml

Table 2: Characteristics of included studies

	Before 2015 (n=32) number, (%)	After 2015 (n=38) number, (%)
**Diagnostic/Prognostic**		
Diagnostic	13 (41%)	4 (11%)
Prognostic	19 (59%)	34 (89%)
**Type**		
Development	14 (44%)	12 (32%)
Validation	4 (13%)	10 (26%)
Development and Validation	10 (31%)	15 (39%)
Update	4 (13%)	1 (3%)
**Setting**		
General population and Primary care	18 (56%)	18 (47%)
Secondary care	14 (44%)	20 (53%)
**Design**		
Cohort	26 (81%)	31 (82%)
RCT	1 (30%)	4 (11%)
Cohort and RCT	2 (6%)	3 (8%)
Case-Control	3 (9%)	0 (0%)
**Topic**		
(Cardio)vascular	12 (38%)	16 (42%)
Oncological	3 (9%)	8 (21%)
Other	17 (53%)	14 (37%)

For peer review only

For peer review only - http://bmjopen.bmj.com/site/about/guidelines.xhtml

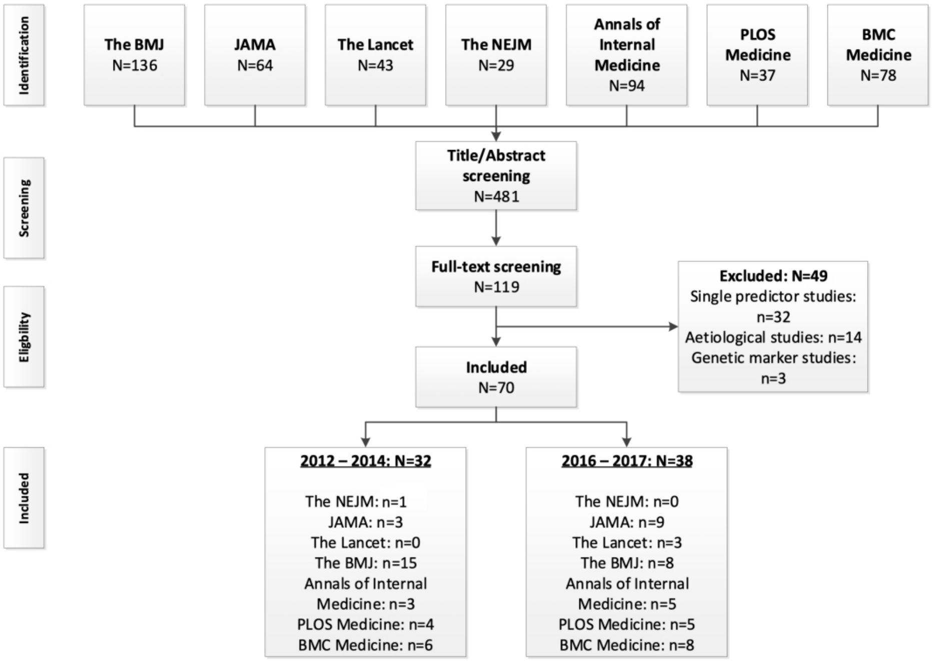

Figure 1: Flow chart of search results and selection procedure

253x190mm (300 x 300 DPI)

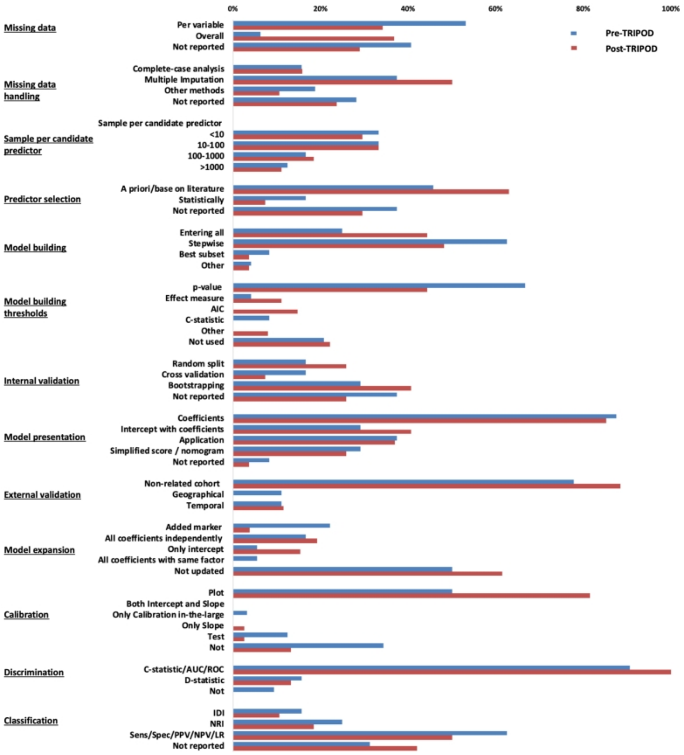

Figure 2: TRIPOD reporting scores

253x190mm (300 x 300 DPI)

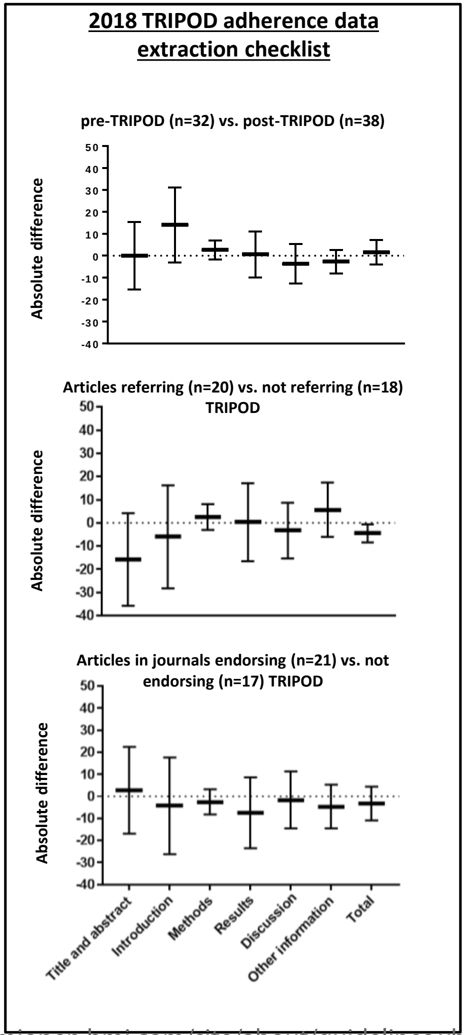
2018 TRIPOD adherence data extraction checklist
pre-TRIPOD (n=32) vs. post-TRIPOD (n=38)
Absolute difference
Articles referring (n=20) vs. not referring (n=18) TRIPOD
Absolute difference
Articles in journals endorsing (n=21) vs. not endorsing (n=17) TRIPOD
Absolute difference
Title and abstract
Introduction
Methods
Results
Discussion
Other information
Total

Supplementary documents

The impact of the TRIPOD statement: a pre-post analysis of reporting and methods.

Amir H Zamanipoor Najafabadi BSc[1,2,3], Chava L Ramspek MD[3], Friedo Dekker[3], Pauline Heus MSc[4,5], Lotty Hooft PhD[4,5], Karel G M Moons MD PhD[4,5], Wilco C Peul MD PhD[1], Gary S Collins PhD[6,7], Ewout W Steyerberg PhD[8], Merel van Diepen PhD[3].

[1]*Department of Neurosurgery, University Neurosugrical Center Holland, Leiden University Medical Center & Haaglanden Medical Center & Haga Teaching Hospitals, Leiden & the Hageue, the Netherlands*
[2]*Department of Neurology, Leiden University Medical Center, Leiden, the Netherlands*
[3]*Department of Clinical Epidemiology, Leiden University Medical Center, Leiden, the Netherlands*
[4]*Cochrane Netherlands, University Medical Center Utrecht, Utrecht University*
[5]*Julius Center for Health Sciences and Primary Care, University Medical Center Utrecht, Utrecht University*
[6]*Centre for Statistics in Medicine, Nuffield Department of Orthopaedics, Rheumatology and Musculoskeletal Sciences, University of Oxford, Oxford, UK.*
[7]*National Institute for Health Research Oxford Biomedical Research Centre, John Radcliffe Hospital, Oxford, UK*
[8]*Department of Biomedical Data Sciences, Leiden University Medical Center, Leiden, The Netherlands.*

Corresponding author: Amir H. Zamanipoor Najafabadi, Department of Neurosurgery, Neurology and Clinical Epidemiology, Leiden University Medical Center, Leiden, the Netherlands. *Tel:* +31-(0)71-5262109. Fax: +31-(0)71-5266987. *Email:* a.h.zamanipoor_najafabadi@lumc.nl

For peer review only

Supplementary Table 1: TRIPOD reporting scores for all included articles

	Before % n=32	After % n=38	Absolute difference of percentages (95% CI)
**Title and abstract**	25	25	0 (-15 to 15)
**Introduction**	69	83	14 (-3 to 31)
**Methods**	80	83	3 (-2 to 7)
**Results**	66	67	1 (-10 to 11)
**Discussion**	89	85	-4 (-13 to 5)
**Other information**	100	97	-3 (-8 to 3)
**Total**	74	76	2 (-4 to 7)

Supplementary Table 2: TRIPOD reporting scores for individual TRIPOD items

TRIPOD item	pre-TRIPOD %	post-TRIPOD %
**1**	34	42
**2**	16	8
**3a**	78	84
**3b**	59	82
**4a**	97	100
**4b**	91	95
**5a**	97	95
**5b**	97	97
**5c**	100	60
**6a**	97	97
**6b**	97	95
**7a**	88	87
**7b**	94	100
**8**	100	100
**9**	28	24
**10a**	63	84
**10b**	19	39
**10c**	67	100
**10d**	69	71
**10e**	56	50
**11**	96	95
**12**	75	71
**13a**	94	95
**13b**	50	37
**13c**	53	65
**14a**	100	97
**14b**	86	64
**15a**	27	42
**15b**	65	61
**16**	66	68
**17**	40	73
**18**	94	97
**19a**	82	75
**19b**	100	95
**20**	75	68
**22**	100	97

For peer review only

Supplementary Table 3: TRIPOD reporting scores for articles published after TRIPOD statement referring vs not referring to the statement

	TRIPOD not referred % n=18	TRIPOD referred % n=20	Absolute difference of percentages (95% CI)
**Title and abstract**	33	18	-16 (-36 to 4)
**Introduction**	86	80	-6 (-28 to 16)
**Methods**	82	84	2 (-3 to 8)
**Results**	67	67	0 (-17 to 17)
**Discussion**	87	83	-3 (-15 to 9)
**Other information**	94	100	6 (-6 to 17)
**Total**	76	76	-1 (-8 to 7)

Supplementary Table 4: TRIPOD reporting scores for articles published after TRIPOD statement in journals that published and did not publish the TRIPOD statement

	TRIPOD not endorsed % n=17	TRIPOD endorsed % n=21	Absolute difference of percentages (95% CI)
**Title and abstract**	24	26	3 (-17 to 22)
**Introduction**	85	81	-4 (-26 to 18)
**Methods**	84	82	-3 (-8 to 3)
**Results**	71	63	-7 (-24 to 9)
**Discussion**	86	84	-2 (-15 to 11)
**Other information**	100	95	-5 (-15 to 5)
**Total**	78	75	-3 (-11 to 4)

Supplementary Table 5: TRIPOD reporting scores for articles published after TRIPOD statement in journals that require adherence to the TRIPOD statement and journals that do not require to the adherence statement

	TRIPOD not required % n=16	TRIPOD required % n=22	Absolute difference of percentages (95% CI)
**Title and abstract**	25	25	0 (-21 to 21)
**Introduction**	81	84	3 (-20 to 26)
**Methods**	83	83	0 (-6 to 6)
**Results**	66	67	1 (-16 to 19)
**Discussion**	90	81	-8 (-20 to 3)
**Other information**	100	95	-5 (-15 to 5)
**Total**	76	76	0 (-8 to 9)

For peer review only

Supplementary Table 6: Percentage articles reporting TRIPOD items in supplementary material

TRIPOD item	Supplement %
**1**	0
**2**	0
**3a**	0
**3b**	0
**4a**	7
**4b**	7
**5a**	10
**5b**	6
**5c**	33
**6a**	13
**6b**	0
**7a**	36
**7b**	0
**8**	3
**9**	9
**10a**	12
**10b**	13
**10c**	14
**10d**	1
**10e**	0
**11**	4
**12**	24
**13a**	21
**13b**	30
**3c**	17
**14a**	2
**14b**	40
**15a**	40
**15b**	35
**16**	0
**17**	17
**18**	0
**19a**	0
**19b**	0
**20**	0
**22**	1

Supplementary figure 1: Average overall TRIPOD reporting levels in percentage per year

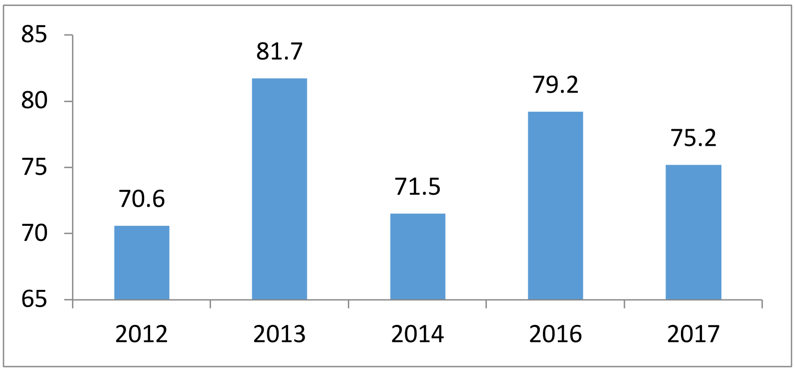

For peer review only

Supplementary Table 7: Performance measures and missing data in all studies

	Before 2015 (n=32) Number (%)	After 2016 (n=38) Number (%)
**Calibration**		
Plot	16 (50%)	31 (82%)
Intercept and Slope	0 (0%)	0 (0%)
Calibration in-the-large	1 (3%)	0 (0%)
Slope	0 (0%)	1 (3%)
Test	4 (13%)	1 (3%)
Not	11 (34%)	5 (13%)
**Discrimination**		
C-statistic / AUC	29 (91%)	38 (100%)
D-statistic	5 (16%)	5 (13%)
Not	3 (9%)	0 (0%)
**Classification**		
IDI	5 (16%)	4 (11%)
NRI	8 (25%)	7 (18%)
Sens. Spec. PPV. NPV. LR. ROC	20 (63%)	19 (50%)
Not reported	10 (31%)	16 (42%)
**Clinical usefulness**		
Decision curve analysis	2 (6%)	8 (21%)
Not reported	30 (94%)	30 (79%)
**Overall performance**		
Brier	2 (6%)	3 (8%)
R2	4 (13%)	5 (13%)
Adequacy statistic	1 (3%)	2 (5%)
Not reported	26 (81%)	28 (74%)
**Missing data reporting**		
Per variable	17 (53%)	13 (34%)
Overall	2 (6%)	14 (37%)
Not reported	13 (41%)	11 (29%)
**Type and reason of missing data**		
Type reported	3 (9%)	2 (5%)
Reason reported	5 (16%)	2 (5%)
**Missing data handling**		
Complete-case analysis	5 (16%)	6 (16%)
Multiple Imputation	12 (38%)	19 (50%)
Other methods	6 (19%)	4 (11%)
Not reported	9 (28%)	9 (24%)

For peer review only

Supplementary Table 8: Model development and presentation

	Before 2015 (n=24) Number (%)	After 2016 (n=27) Number (%)
**Sample per candidate predictor**		
<10	8 (33%)	8 (30%)
10-100	8 (33%)	9 (33%)
100-1000	8 (17%)	5 (19%)
>1000	3 (13%)	3 (11%)
Unknown number of predictors	0 (0%)	1 (4%)
Unknown number of outcomes	1 (4%)	1 (4%)
**Model Type**		
Linear	1 (4%)	0 (0%)
Logistic	16 (67%)	15 (56%)
Cox	6 (25%)	10 (37%)
Points	0 (0%)	2 (7%)
Other	1 (4%)	0 (0%)
**Predictor Selection**		
A priori knowledge / based on literature	11 (46%)	17 (63%)
Statistically	4 (17%)	2 (7%)
Not reported	9 (38%)	8 (30%)
**Model building**		
Entering all	6 (25%)	12 (44%)
Stepwise	15 (63%)	13 (48%)
Best subset	2 (8%)	1 (4%)
Other	1 (4%)	1 (4%)
**Model building thresholds**		
p-value	16 (67%)	12 (44%)
Effect measure	1 (4%)	3 (11%)
R	0 (0%)	1 (4%)
Manually	0 (0%)	1 (4%)
AIC	0 (0%)	4 (15%)
C-statistic	2 (8%)	0 (0%)
Other	0 (0%)	0 (0%)
Not used	5 (21%)	6 (22%)
**Internal validation**		
Random split	4 (17%)	7 (26%)
Cross validation	4 (17%)	2 (7%)
Bootstrapping	7 (29%)	11 (41%)
Not reported	9 (38%)	7 (26%)
**Model presentation**		
Coefficients	21 (88%)	23 (85%)
Intercept with coefficients	7 (29%)	11 (41%)
Application	9 (38%)	10 (37%)
Simplified score / nomogram	7 (29%)	7 (26%)
Not reported	2 (8%)	1 (4%)

For peer review only

1 **Supplementary Table 9: External validation and updating**

	Before 2015 (n=18) Number (%)	After 2016 (n=26) Number (%)
**External validation**		
Fully independent	14 (78%)	23 (88%)
Geographical	2 (11%)	0 (0%)
Temporal	2 (11%)	3 (12%)
**Model updating**		
Added marker	4 (22%)	1 (4%)
All coefficients independently	3 (17%)	5 (19%)
Only intercept	1 (6%)	4 (15%)
All coefficients with same factor	1 (6%)	0 (0%)
Not updated	9 (50%)	16 (62%)

2
3

For peer review only

Supplementary text 1: Protocol

Article selection

Search strategy:

("The New England journal of medicine"[Journal] OR "Lancet (London, England)"[Journal] OR "BMJ (Clinical research ed.)"[Journal] OR "*JAMA*"[Journal] OR "PLOS Medicine"[Journal] OR "*Annals of Internal Medicine*"[Journal] OR "*BMC Medicine*"[Journal]) AND (predict*[ti] OR prognost*[ti] OR diagnostic*[ti] OR “risk score”[ti]) NOT ("Animals"[mesh] NOT "Humans"[mesh]) NOT (("case reports"[ptyp] OR "case report"[ti]) NOT ("Review"[ptyp] OR "clinical study"[ptyp] OR "case series"[tw]))

Articles included

1 Abbasi A, Peelen LM, Corpeleijn E, *et al.* Prediction models for risk of developing type 2 diabetes: Systematic literature search and independent external validation study. *BMJ* 2012;**345**:1–16. doi:10.1136/bmj.e5900

2 Bejnordi BE, Veta M, Van Diest PJ, *et al.* Diagnostic assessment of deep learning algorithms for detection of lymph node metastases in women with breast cancer. *JAMA - J Am Med Assoc* 2017;**318**:2199–210. doi:10.1001/jama.2017.14585

3 Collins GS, Altman DG. Predicting the 10 year risk of cardiovascular disease in the United Kingdom: Independent and external validation of an updated version of QRISK2. *BMJ* 2012;**345**:1–12. doi:10.1136/bmj.e4181

4 Costa F, van Klaveren D, James S, *et al.* Derivation and validation of the predicting bleeding complications in patients undergoing stent implantation and subsequent dual antiplatelet therapy (PRECISE-DAPT) score: a pooled analysis of individual-patient datasets from clinical trials. *Lancet* 2017;**389**:1025–34. doi:10.1016/S0140-6736(17)30397-5

5 Cubiella J, Vega P, Salve M, *et al.* Development and external validation of a faecal immunochemical test-based prediction model for colorectal cancer detection in symptomatic patients. *BMC Med* 2016;**14**:1–13. doi:10.1186/s12916-016-0668-5

6 D.S. L, A. S, P.C. A, *et al.* Prediction of heart failure mortality in emergent care: A cohort study. *Ann Intern Med* 2012;**156**:767–75. doi:10.7326/0003-4819-156-11-201206050-00003

7 Dagan N, Cohen-Stavi C, Leventer-Roberts M, *et al.* External validation and comparison of three prediction tools for risk of osteoporotic fractures using data from population based electronic health records: Retrospective cohort study. *BMJ* 2017;**356**. doi:10.1136/bmj.i6755

8 Dalton JE, Perzynski AT, Zidar DA, *et al.* Accuracy of cardiovascular risk prediction varies by neighborhood socioeconomic position a retrospective cohort study. *Ann Intern Med* 2017;**167**:456–64. doi:10.7326/M16-2543

9 Dalziel SR, Thompson JMD, Macias CG, *et al.* Predictors of severe H1N1 infection in children presenting within Pediatric Emergency Research Networks (PERN): Retrospective case-control study. *BMJ* 2013;**347**:1–13. doi:10.1136/bmj.f4836

10 Terfc. Glycated hemoglobin measurement and prediction of cardiovascular disease. *JAMA - J Am Med Assoc* 2014;**311**:1225–33. doi:10.1001/jama.2014.1873

11 Elias SG, Kok L, de Wit NJ, *et al.* Is there an added value of faecal calprotectin and haemoglobin in the diagnostic work-up for primary care patients suspected of significant colorectal disease? A cross-sectional diagnostic study. *BMC Med* 2016;**14**:1–11. doi:10.1186/s12916-016-0684-5

12 Kaptoge. C-Reactive Protein, Fibrinogen, and Cardiovascular Disease Prediction. *N Engl J Med* 2012;**367**:1310–20. doi:10.1056/NEJMoa1107477

13 Esplin MS, Elovitz MA, Iams JD, *et al.* Predictive accuracy of serial transvaginal cervical lengths and quantitative vaginal fetal fibronectin levels for spontaneous preterm birth among nulliparous women. *JAMA - J Am Med Assoc* 2017;**317**:1047–56. doi:10.1001/jama.2017.1373

For peer review only

14 Fischer K, Kettunen J, Würtz P, *et al.* Biomarker Profiling by Nuclear Magnetic Resonance Spectroscopy for the Prediction of All-Cause Mortality: An Observational Study of 17,345 Persons. *PLoS Med* 2014;**11**. doi:10.1371/journal.pmed.1001606
15 Fraccaro P, van der Veer S, Brown B, *et al.* An external validation of models to predict the onset of chronic kidney disease using population-based electronic health records from Salford, UK. *BMC Med* 2016;**14**:1–15. doi:10.1186/s12916-016-0650-2
16 Freund Y, Lemachatti N, Krastinova E, *et al.* Prognostic accuracy of sepsis-3 criteria for in-hospital mortality among patients with suspected infection presenting to the emergency department. *JAMA - J Am Med Assoc* 2017;**317**:301–8. doi:10.1001/jama.2016.20329
17 Ganz P, Heidecker B, Hveem K, *et al.* Development and validation of a protein-based risk score for cardiovascular outcomes among patients with stable coronary heart disease. *JAMA - J Am Med Assoc* 2016;**315**:2532–41. doi:10.1001/jama.2016.5951
18 Genders TSS, Steyerberg EW, Hunink MGM, *et al.* Prediction model to estimate presence of coronary artery disease: Retrospective pooled analysis of existing cohorts. *BMJ* 2012;**344**:1–13. doi:10.1136/bmj.e3485
19 Gnanapragasam VJ, Lophatananon A, Wright KA, *et al.* Improving Clinical Risk Stratification at Diagnosis in Primary Prostate Cancer: A Prognostic Modelling Study. *PLoS Med* 2016;**13**:1–18. doi:10.1371/journal.pmed.1002063
20 Goldstick JE, Carter PM, Walton MA, *et al.* Development of the SaFETy score: A clinical screening tool for predicting future firearm violence risk. *Ann Intern Med* 2017;**166**:707–14. doi:10.7326/M16-1927
21 Goodacre S, Wilson R, Shephard N, *et al.* Derivation and validation of a risk adjustment model for predicting seven day mortality in emergency medical admissions: Mixed prospective and retrospective cohort study. *BMJ* 2012;**344**:1–11. doi:10.1136/bmj.e2904
22 den ruitjer H, Rembold CM. Common Carotid Intima-Media Thickness Measurements in Cardiovascular Risk Prediction. *J Am Med Assoc* 2015;**308**:796–803.http://jama.jamanetwork.com/
23 Hijazi Z, Oldgren J, Lindbäck J, *et al.* The novel biomarker-based ABC (age, biomarkers, clinical history)-bleeding risk score for patients with atrial fibrillation: a derivation and validation study. *Lancet* 2016;**387**:2302–11. doi:10.1016/S0140-6736(16)00741-8
24 Hippisley-Cox J, Coupland C. Development and validation of risk prediction equations to estimate survival in patients with colorectal cancer: Cohort study. *BMJ* 2017;**357**. doi:10.1136/bmj.j2497
25 Hippisley-Cox J, Coupland C, Brindle P. Development and validation of QRISK3 risk prediction algorithms to estimate future risk of cardiovascular disease: Prospective cohort study. *BMJ* 2017;**357**. doi:10.1136/bmj.j2099
26 Hippisley-Cox J. Predicting risk of upper gastrointestinal bleed and intracranial bleed with anticoagulants: Cohort study to derive and validate the QBleed scores. *BMJ* 2014;**349**:1–21. doi:10.1136/bmj.g4606
27 Hippisley-Cox J, Coupland C, Brindle P. Derivation and validation of QStroke score for predicting risk of ischaemic stroke in primary care and comparison with other risk scores: A prospective open cohort study. *BMJ* 2013;**346**:1–15. doi:10.1136/bmj.f2573
28 Hippisley-Cox J, Coupland C. Derivation and validation of updated QFracture algorithm to predict risk of osteoporotic fracture in primary care in the United Kingdom: Prospective open cohort study. *BMJ* 2012;**345**:1–16. doi:10.1136/bmj.e3427
29 Hippisley-Cox J, Coupland C. Development and validation of QMortality risk prediction algorithm to estimate short term risk of death and assess frailty: cohort study. *BMJ* 2017;**358**:j4208. doi:10.1136/bmj.j4208
30 J.M. Q, C. EE, A. U, *et al.* Predictive score for mortality in patients with COPD exacerbations attending hospital emergency departments. *BMC Med* 2014;**12**:66. doi:10.1186/1741-7015-12-66
31 James MT, Pannu N, Hemmelgarn BR, *et al.* Derivation and external validation of prediction models for advanced chronic kidney disease following acute kidney injury. *JAMA - J Am Med Assoc* 2017;**318**:1787–97. doi:10.1001/jama.2017.16326
32 Kavousi M, Desai CS, Ayers C, *et al.* Prevalence and prognostic implications of coronary artery calcification in low-risk women: A meta-analysis. *JAMA - J Am Med Assoc* 2016;**316**:2126–34.

For peer review only

doi:10.1001/jama.2016.17020
33 Kent M, Penson DF, Albertsen PC, *et al.* Successful external validation of a model to predict other cause mortality in localized prostate cancer. *BMC Med* 2016;**14**:1–7. doi:10.1186/s12916-016-0572-z
34 Perel P. Predicting early death in patients with traumatic bleeding: development and validation of prognostic model. *J Geophys Res* 1989;**94**:3579. doi:10.1136/bmj.e5166
35 Kugathasan S, Denson LA, Walters TD, *et al.* Prediction of complicated disease course for children newly diagnosed with Crohn's disease: a multicentre inception cohort study. *Lancet* 2017;**389**:1710–8. doi:10.1016/S0140-6736(17)30317-3
36 Lamain-de Ruiter M, Kwee A, Naaktgeboren CA, *et al.* External validation of prognostic models to predict risk of gestational diabetes mellitus in one Dutch cohort: prospective multicentre cohort study. *BMJ* 2016;**354**:i4338. doi:10.1136/bmj.i4338
37 Little P, Stuart B, Richard Hobbs FD, *et al.* Predictors of suppurative complications for acute sore throat in primary care: Prospective clinical cohort study. *BMJ* 2013;**347**:1–14. doi:10.1136/bmj.f6867
38 Wang s w. Anti-allergic activity of some selected plants in the genus Boesenbergia and Kaempferia. *Songklanakarin J Sci Technol* 2011;**33**:301–4. doi:10.1136/bmj.i2607
39 Dewland TA. Atrial Ectopy as a Predictor of Incident Atrial Fibrillation. 2011;**4**:721–8. doi:10.1126/scisignal.2001449.Engineering
40 Marcantonio ER, Ngo LH, O'Connor M, *et al.* 3D-CAM: Derivation and validation of a 3-minute diagnostic interview for CAM-defined delirium: A cross-sectional diagnostic test study. *Ann Intern Med* 2014;**161**:554–61. doi:10.7326/M14-0865
41 Matsushita K, Woodward M, Jafar TH, *et al.* Comparison of Risk Prediction Using the CKD-EPI Equation and the MDRD Study Equation for Estimated Glomerular Filtration Rate. 2012;**307**:1941–51.
42 McLernon DJ, Steyerberg EW, Te Velde ER, *et al.* Predicting the chances of a live birth after one or more complete cycles of in vitro fertilisation: Population based study of linked cycle data from 113 873 women. *BMJ* 2016;**355**. doi:10.1136/bmj.i5735
43 Miro O, Rossello X, Gil V, *et al.* Predicting 30-day mortality for patients with acute heart failure in the emergency department. *Ann Intern Med* 2017;**167**:698–705. doi:10.7326/M16-2726
44 Moore CL, Bomann S, Daniels B, *et al.* Derivation and validation of a clinical prediction rule for uncomplicated ureteral stone-the STONE score: Retrospective and prospective observational cohort studies. *BMJ* 2014;**348**:1–12. doi:10.1136/bmj.g2191
45 Mwangi MN, Maskey S, Andang'o PEA, *et al.* Diagnostic utility of zinc protoporphyrin to detect iron deficiency in Kenyan pregnant women. *BMC Med* 2014;**12**:1–13. doi:10.1186/s12916-014-0229-8
46 Nijman RG, Vergouwe Y, Thompson M, *et al.* Clinical prediction model to aid emergency doctors managing febrile children at risk of serious bacterial infections: Diagnostic study. *BMJ* 2013;**346**:1–16. doi:10.1136/bmj.f1706
47 Koller MT. Original Research Older U . S . and European Persons in the Cardiovascular Health Study. *Ann Intern Med* 2012.
48 Patel VB, Theron G, Lenders L, *et al.* Diagnostic Accuracy of Quantitative PCR (Xpert MTB/RIF) for Tuberculous Meningitis in a High Burden Setting: A Prospective Study. *PLoS Med* 2013;**10**. doi:10.1371/journal.pmed.1001536
49 Pavese C, Schneider MP, Schubert M, *et al.* Prediction of Bladder Outcomes after Traumatic Spinal Cord Injury: A Longitudinal Cohort Study. *PLoS Med* 2016;**13**:1–16. doi:10.1371/journal.pmed.1002041
50 Payne BA, Hutcheon JA, Ansermino JM, *et al.* A Risk Prediction Model for the Assessment and Triage of Women with Hypertensive Disorders of Pregnancy in Low-Resourced Settings: The miniPIERS (Pre-eclampsia Integrated Estimate of RiSk) Multi-country Prospective Cohort Study. *PLoS Med* 2014;**11**. doi:10.1371/journal.pmed.1001589
51 Pfeiffer RM, Park Y, Kreimer AR, *et al.* Risk Prediction for Breast, Endometrial, and Ovarian Cancer in White Women Aged 50 y or Older: Derivation and Validation from Population-Based Cohort Studies. *PLoS Med* 2013;**10**. doi:10.1371/journal.pmed.1001492

For peer review only

52 Q. L, M. IDK, T.K. T, *et al.* A clinical prediction rule for diagnosing human infections with avian influenza A(H7N9) in a hospital emergency department setting. *BMC Med* 2014;**12**:1–9. doi:10.1186/s12916-014-0127-0 LK - http://sfx.hul.harvard.edu/sfx_local?sid=EMBASE&issn=17417015&id=doi:10.1186%2Fs12916-014-0127-0&atitle=A+clinical+prediction+rule+for+diagnosing+human+infections+with+avian+influenza+A%28H7N9%29+in+a+hospital+emergency+department+setting&stitle=BMC+Med.&title=BMC+Medicine&volume=12&issue=1&spage=&epage=&aulast=Liao&aufirst=Qiaohong&auinit=Q.&aufull=Liao+Q.&coden=&isbn=&pages=-&date=2014&auinit1=Q&auinitm=

53 Raith EP, Udy AA, Bailey M, *et al.* Prognostic accuracy of the SOFA score, SIRS criteria, and qSOFA score for in-hospital mortality among adults with suspected infection admitted to the intensive care unit. *JAMA - J Am Med Assoc* 2017;**317**:290–300. doi:10.1001/jama.2016.20328

54 Pandie S. Diagnostic accuracy of quantitative PCR (Xpert MTB/RIF) for tuberculous pericarditis compared to adenosine deaminase and unstimulated interferon-γ in a high burden setting: A prospective study. *BMC Med* 2014;**12**:2–11. doi:10.1186/1741-7015-12-101

55 Raji O y. Annals of Internal Medicine Predictive Accuracy of the Liverpool Lung Project Risk Model for Stratifying Patients for Computed Tomography Screening for Lung Cancer. 2012.

56 Sultan AA, West J, Grainge MJ, *et al.* Development and validation of risk prediction model for venous thromboembolism in postpartum women: Multinational cohort study. *BMJ* 2016;**355**. doi:10.1136/bmj.i6253

57 Tangri N, Grams ME, Levey AS, *et al.* Multinational assessment of accuracy of equations for predicting risk of kidney failure ameta-analysis. *JAMA - J Am Med Assoc* 2016;**315**:164–74. doi:10.1001/jama.2015.18202

58 ten Haaf K, Jeon J, Tammemägi MC, *et al.* Risk prediction models for selection of lung cancer screening candidates: A retrospective validation study. *PLoS Med* 2017;**14**:1–24. doi:10.1371/journal.pmed.1002277

59 Thangaratinam S, Allotey J, Marlin N, *et al.* Prediction of complications in early-onset pre-eclampsia (PREP): Development and external multinational validation of prognostic models. *BMC Med* 2017;**15**:1–11. doi:10.1097/QAD.0b013e3282efacb1

60 Traeger AC, Henschke N, Hübscher M, *et al.* Estimating the Risk of Chronic Pain: Development and Validation of a Prognostic Model (PICKUP) for Patients with Acute Low Back Pain. *PLoS Med* 2016;**13**:1–21. doi:10.1371/journal.pmed.1002019

61 Van Calster B, Van Hoorde K, Valentin L, *et al.* Evaluating the risk of ovarian cancer before surgery using the ADNEX model to differentiate between benign, borderline, early and advanced stage invasive, and secondary metastatic tumours: Prospective multicentre diagnostic study. *BMJ* 2014;**349**:1–14. doi:10.1136/bmj.g5920

62 Van Den Boogaard M, Pickkers P, Slooter AJC, *et al.* Development and validation of PRE-DELIRIC (PREdiction of DELIRium in ICu patients) delirium prediction model for intensive care patients: Observational multicentre study. *BMJ* 2012;**344**:17. doi:10.1136/bmj.e420

63 Van den Bruel A, Thompson M, Stevens R, *et al.* How well do clinical prediction rules perform in identifying serious infections in acutely ill children across an international network of ambulatory care datasets?. *BMC Med* 2013;**11**:10.http://ovidsp.ovid.com/ovidweb.cgi?T=JS&PAGE=reference&D=medc&NEWS=N&AN=23320738

64 Van Vugt SF, Broekhuizen BDL, Lammens C, *et al.* Use of serum C reactive protein and procalcitonin concentrations in addition to symptoms and signs to predict pneumonia in patients presenting to primary care with acute cough: Diagnostic study. *BMJ* 2013;**346**:1–12. doi:10.1136/bmj.f2450

65 Viera AJ, Garrett JM. Understanding interobserver agreement: The kappa statistic. *Fam Med* 2005;**37**:360–3.

66 W.A. L, R. D, L.A. K, *et al.* Serum protein profiles predict coronary artery disease in symptomatic patients referred for coronary angiography. *BMC Med* 2012;**10**:157. doi:10.1186/1741-7015-10-157

67 Walters K, Hardoon S, Petersen I, *et al.* Predicting dementia risk in primary care: Development and

For peer review only

validation of the Dementia Risk Score using routinely collected data. *BMC Med* 2016;**14**:1–12. doi:10.1186/s12916-016-0549-y
68 Yeh RW, Secemsky EA, Kereiakes DJ, *et al.* Development and validation of a prediction rule for benefit and harm of Dual antiplatelet therapy beyond 1 year after percutaneous coronary intervention. *JAMA - J Am Med Assoc* 2016;**315**:1735–49. doi:10.1001/jama.2016.3775
69 Zemek R, Barrowman N, Freedman SB, *et al.* Clinical risk score for persistent postconcussion symptomsamong children with acute concussion in the ED. *JAMA - J Am Med Assoc* 2016;**315**:1014–25. doi:10.1001/jama.2016.1203
70 Farinati F, Vitale A, Spolverato G, *et al.* Development and Validation of a New Prognostic System for Patients with Hepatocellular Carcinoma. *PLoS Med* 2016;**13**:1–18. doi:10.1371/journal.pmed.1002006

For peer review only

Assessment of study characteristics, used methods and specific reporting items

3 Background and Objectives	
3a: Type of predictive study	1. Prognostic<br>2. Diagnostic
3a: Topic Study	1. (Cardio)vascular<br>2. Oncological<br>3. Surgical<br>4. Other
3b: Model development and validation	1. Development<br>2. Validation<br>3. Development and Validation<br>4. Updating

4 Source of Data	
4a: Study design	1. RCT<br>2. Prospective cohort<br>3. Retrospective cohort<br>4. Nested case-control<br>5. Non-nested case-control

5 Participants	
5a: Study Setting	1. General population<br>2. Primary care<br>3. Secondary care

6 Outcome	
6a: Definition outcome	1. What is the outcome?<br>2. What is the time frame?
6a: Type of outcome	1. Dichotomous,<br>2. Continuous<br>3. Ordinal<br>4. Nominal<br>5. Time to event

9 Missing Data	
9 Type	1. MAR<br>2. MCAR<br>3. MNAR
9 Reason	Reason missing data
9 Handling	1. Complete-case analysis<br>2. Single imputation<br>3. Multiple imputation

For peer review only

	4. Missing indicator method<br>5. Last observation carried forward<br>6. Mean imputation<br>7. Other

10 Statistical Analysis and Methods	
10a: Analysis of predictors	1. Categorical<br>2. Linear: kept linear<br>3. Linear: dichotomized<br>4. Linear: categorized<br>5. Linear: polynomial transformation<br>6. Linear: spline transformation,<br>7. Linear: interaction<br>8. Linear: log-transformation<br>9. Other<br>(multiple options possible)
10b: Type of Model	1. Linear<br>2. Logistic<br>3. Ordinal<br>4. Nominal<br>5. Survival<br>6. Poisson<br>7. Points<br>8. Other
10b: Selection Predictors	1. A priori knowledge<br>2. Based on the literature<br>3. Statistically<br>(multiple options possible)
10b: Model building procedure	1. Entering all predictors<br>2. Forward selection<br>3. Backward selection<br>4. Best subset
10b: Statistical thresholds for model building	1. p-value: threshold<br>2. AIC/BIC<br>3. R^2<br>4. C-statistic/AUC<br>5. HR<br>6. Manually
10b: Internal Validation methods	1. Apparent<br>2. Random split<br>3. Cross-validation<br>4. Bootstrapping<br>(multiple options possible)

For peer review only

10d: External Validation methods	1. Temporal validation<br>2. Geographical validation<br>3. Fully independent<br>4. Other<br>(Multiple options possible)
10d: Performance Calibration	1. Test<br>2. Slope<br>3. Intercept and slope<br>4. Calibration in-the-large<br>5. Plot<br>(multiple options possible)
10d: Performance Discrimination	1. C-statistic / AUC after Development<br>2. C-statistic / AUC after Internal validation<br>3. C-statistic / AUC after external validation<br>4. D-statistic
10d: Performance Classification	1. Sensitivity / Specificity<br>2. Positive Predictive Value / Negative Predictive Value<br>3. Likelihood ratio<br>(multiple options possible)
10d: Performance Other	1. IDI<br>2. NRI<br>3. Decision curve analysis<br>(multiple options possible)
10d: Performance Overall	1. Brier<br>2. 2. R^2
10e: Model Updating	1. Intercept<br>2. all coefficients changed with same factor<br>3. Re-estimation all coefficients independently<br>4. 2+ selection of additional predictors<br>5. 3+ Reestimation all coefficients

13 Participants	
13a: Number of Participants	1. Number of participants with and without outcome
13b: Missing Values	Reported for<br>1. Any value<br>2. Per predictor<br>3. For predictors in general<br>4. For outcome<br>(multiple options possible)

Model Specification	
15a Intercept or baseline hazard	

For peer review only

15a: Coefficients	Coefficients reported:<br>1. All univariate<br>2. Univariate of included predictors in final model<br>3. All multivariate<br>4. Multivariate of predictors included in final model<br>(multiple option possible)
15a: Number of predictors	Number of predictors in final model
15a: Use of model	Use of model reported as:<br>1. Only regression coefficients<br>2. Intercept with regression coefficients<br>3. Simplified score (incl. nomogram or app)<br>(multiple options possible)

For peer review only

BMJ Open

TRIPOD statement: a preliminary pre-post analysis of reporting and methods of prediction models

Journal:	*BMJ Open*
Manuscript ID	bmjopen-2020-041537.R1
Article Type:	Original research
Date Submitted by the Author:	21-Jul-2020
Complete List of Authors:	Zamanipoor Najafabadi, Amir H.; Leiden University Medical Center<br>Ramspek, Chava; Leids Universitair Medisch Centrum, Clinical Epidemiology<br>Dekker, Friedo; Leiden University Medical Center, Department of Clinical Epidemiology<br>Heus, Pauline; University Medical Center Utrecht, Utrecht University, Cochrane Netherlands, Julius Center for Health Sciences and Primary Care<br>Hooft, Lotty; University Medical Centre (UMC) Utrecht, University Utrecht (UU), Dutch Cochrane Centre (DCC), Julius Center for Health Sciences and Primary Care<br>Moons, Karel; Julius Center for Health Sciences and Primary Care, Epidemiology<br>Peul, Wilco; Leiden University Medical Center, Neurosurgery; Medical Center The Hague, Neurosurgery<br>Collins, Gary; Oxford University, NDORMS<br>Steyerberg, Ewout; Leiden University Medical Center, Department of Medical Statistics<br>van Diepen, Merel; Leiden University Medical Center, Clinical Epidemiology
**Primary Subject Heading**:	Epidemiology
Secondary Subject Heading:	Research methods
Keywords:	STATISTICS & RESEARCH METHODS, EPIDEMIOLOGY, GENERAL MEDICINE (see Internal Medicine)

SCHOLARONE™
Manuscripts

I, the Submitting Author has the right to grant and does grant on behalf of all authors of the Work (as defined in the below author licence), an exclusive licence and/or a non-exclusive licence for contributions from authors who are: i) UK Crown employees; ii) where BMJ has agreed a CC-BY licence shall apply, and/or iii) in accordance with the terms applicable for US Federal Government officers or employees acting as part of their official duties; on a worldwide, perpetual, irrevocable, royalty-free basis to BMJ Publishing Group Ltd ("BMJ") its licensees and where the relevant Journal is co-owned by BMJ to the co-owners of the Journal, to publish the Work in this journal and any other BMJ products and to exploit all rights, as set out in our licence.

The Submitting Author accepts and understands that any supply made under these terms is made by BMJ to the Submitting Author unless you are acting as an employee on behalf of your employer or a postgraduate student of an affiliated institution which is paying any applicable article publishing charge ("APC") for Open Access articles. Where the Submitting Author wishes to make the Work available on an Open Access basis (and intends to pay the relevant APC), the terms of reuse of such Open Access shall be governed by a Creative Commons licence – details of these licences and which Creative Commons licence will apply to this Work are set out in our licence referred to above.

Other than as permitted in any relevant BMJ Author's Self Archiving Policies, I confirm this Work has not been accepted for publication elsewhere, is not being considered for publication elsewhere and does not duplicate material already published. I confirm all authors consent to publication of this Work and authorise the granting of this licence.

TRIPOD statement: a preliminary pre-post analysis of reporting and methods of prediction models

Amir H Zamanipoor Najafabadi BSc[1,2,3], Chava L Ramspek MD[3], Friedo W Dekker[3], Pauline Heus MSc[4,5], Lotty Hooft PhD[4,5], Karel G M Moons MD PhD[4,5], Wilco C Peul MD PhD[1], Gary S Collins PhD[6,7], Ewout W Steyerberg PhD[8], Merel van Diepen PhD[3].

[1]*Department of Neurosurgery, University Neurosurgical Center Holland, Leiden University Medical Center & Haaglanden Medical Center & Haga Teaching Hospitals, Leiden & the Hageue, the Netherlands*
[2]*Department of Neurology, Leiden University Medical Center, Leiden, the Netherlands*
[3]*Department of Clinical Epidemiology, Leiden University Medical Center, Leiden, the Netherlands*
[4]*Cochrane Netherlands, University Medical Center Utrecht, Utrecht University*
[5]*Julius Center for Health Sciences and Primary Care, University Medical Center Utrecht, Utrecht University*
[6]*Centre for Statistics in Medicine, Nuffield Department of Orthopaedics, Rheumatology and Musculoskeletal Sciences, University of Oxford, Oxford, UK.*
[7]*National Institute for Health Research Oxford Biomedical Research Centre, John Radcliffe Hospital, Oxford, UK*
[8]*Department of Biomedical Data Sciences, Leiden University Medical Center, Leiden, The Netherlands.*

Corresponding author: Amir H. Zamanipoor Najafabadi, Department of Neurosurgery, Neurology and Clinical Epidemiology, Leiden University Medical Center, Leiden, the Netherlands. *Tel:* +31-(0)71-5262109. Fax: +31-(0)71-5266987. *Email:* a.h.zamanipoor_najafabadi@lumc.nl

**Type of article:**	Research
**Figures / Tables:**	3 figures and 2 Tables
**Word count main text:**	3550
**Word count abstract:**	320

For peer review only - http://bmjopen.bmj.com/site/about/guidelines.xhtml

ABSTRACT

Objectives

To assess the difference in completeness of reporting and methodological conduct of published prediction models before and after publication of the Transparent Reporting of a multivariable prediction model for Individual Prognosis Or Diagnosis (TRIPOD) Statement.

Methods

In the seven general medicine journals with the highest impact factor, we compared the completeness of the reporting and the quality of the methodology of prediction model studies published between 2012 and 2014 (pre-TRIPOD) with studies published between 2016 and 2017 (post-TRIPOD). For articles published in the post-TRIPOD period, we examined whether there was improved reporting for articles (1) citing the TRIPOD Statement, and (2) published in journals that published the TRIPOD Statement.

Results

A total of 70 articles were included (pre-TRIPOD: 32, post-TRIPOD: 38). No improvement was seen for the overall percentage of reported items after the publication of the TRIPOD Statement (pre-TRIPOD 74%, post-TRIPOD 76%, 95% CI of absolute difference: -4% to 7%). For the individual TRIPOD items, an improvement was seen for 16 (44%) items, while 3 (8%) items showed no improvement and 17 (47%) items showed a deterioration. Post-TRIPOD, there was no improved reporting for articles citing the TRIPOD statement, nor for articles published in journals that published the TRIPOD statement. The methodological quality improved in the post-TRIPOD period. More models were externally validated in the same article (absolute difference 8%, post-TRIPOD: 39%), used measures of calibration (21%, post-TRIPOD: 87%) and discrimination (9%, post-TRIPOD: 100%), and used multiple imputation for handling missing data (13%, post-TRIPOD: 50%).

Conclusions

Since the publication of the TRIPOD Statement, some reporting and methodological aspects have improved. Prediction models are still often poorly developed and validated and many aspects remain poorly reported, hindering optimal clinical application-of these models. Long-term effects of the TRIPOD statement publication should be evaluated in future studies.

KEY WORDS: Diagnostic, Prediction, Prognostic, Reporting, TRIPOD, Methodology.

For peer review only - http://bmjopen.bmj.com/site/about/guidelines.xhtml

Strengths and limitations of this study

- This is the first study to assess the completeness of reporting and methodological conduct of prediction models published before and after publication of the TRIPOD statement.
- A limitation of this study is the short time period evaluated and therefore future studies are needed to assess the long-term effects on completeness of reporting and methodological conduct.
- Causality between publication of the TRIPOD statement and the found results cannot be established due to confounding.

For peer review only

For peer review only - http://bmjopen.bmj.com/site/about/guidelines.xhtml

INTRODUCTION

Prediction models cover both prognostic models, which aim to predict the risk of future outcomes, and diagnostic models, which aim to assess the presence or absence of a condition.[1] They provide information for differential diagnosis, additional testing and for patient selection on treatment. Interest in prediction models has sharply increased over the last two decades, translating to new methodological developments, especially regarding performance assessment of these models[2–4]. In addition, clinical guidelines are increasingly recommending the use of prediction models,[5,6] and consequently implementation of these models in clinical practice for individualised diagnostic and therapeutic decisions has surged.[7–10]

Previous systematic reviews on the quality of published prediction models have identified poor reporting and many methodological shortcomings in the development and validation of these models.[11–13] In response to these reviews, the Transparent Reporting of a multivariable prediction model for Individual Prognosis Or Diagnosis (TRIPOD) Statement was developed.[14] The TRIPOD Statement provides reporting recommendations for articles that describe the development and external validation of prediction models, aiming to enhance reporting transparency and hence interpretability, reproducibility, and clinical usability of these models.[14] Although the TRIPOD Statement primarily focuses on reporting and not on methods, current accepted methods for the development and validation of prediction models are discussed in the accompanied Explanation and Elaboration document.[15]

The primary aim of this study was to assess the difference in completeness of reporting and methodological conduct of published prediction models before and after publication in high impact general medicine journals.

For peer review only - http://bmjopen.bmj.com/site/about/guidelines.xhtml

METHODS

Systematic literature search

We selected the seven general medicine journals with the highest Web of Knowledge impact factor in 2017: *New England Journal of Medicine (NEMJ), Journal of the American Medical Association (JAMA), The Lancet, the British Medical Journal (The BMJ), Annals of Internal Medicine, PLOS Medicine, and BMC Medicine*. Articles on prediction models published in these journals before publication of the TRIPOD Statement (pre-TRIPOD: 01 January 2012 – 31 December 2014) and after publication of TRIPOD statement (post-TRIPOD: 01 January 2016 – 31 December 2017) were identified by a PubMed search string (Supplementary text 1). Articles published in 2015 were excluded from the search, as the TRIPOD Statement was published in 2015 and we regard this as a transition period. Titles and abstracts were screened by one reviewer (AHZN). Full-text articles were screened by two independent reviewers (AHZN and CLR) and disagreement was resolved by discussion and consensus with a senior author (MvD).

Article and model selection

Original articles with the primary aim of developing and/or validating multivariable models, both prognostic and diagnostic, were included. We excluded etiological studies, genetic marker studies, and model impact studies, as these are not covered by the TRIPOD Statement. Included articles were classified as 1) development, 2) development and external validation, 3) external validation, and 4) extension/updating of models. For articles addressing multiple models but not explicitly recommending a single model, the model with the most predictors was evaluated. For instance, Hippisley-Cox (2013) described model A, B and C for the prediction of future risk of cardiovascular disease, with model B being the same as model A with the addition of several predictors and interactions and model C being the same as model B with the addition of one variable. In this case model C was evaluated.

Assessment of adherence to TRIPOD criteria

In 2018, authors of the TRIPOD Statement published a TRIPOD adherence assessment form and adherence scoring rules, which were also used in our study. [16–18] The TRIPOD Adherence form is a measurement tool developed for authors who want to evaluate the adherence of prediction model studies to TRIPOD, e.g. over time or in a certain medical domain. In general, when multiple aspects were described within a TRIPOD item, all aspects needed to be reported to score a point for that item. For instance, the item title contains four sub-items (e.g. i. identifying the study as development and/or validation of a ii. prediction model with iii. description of target population and iv. outcome) and all four aspects need to be reported to score a point for this specific item. For all items and aspects of the checklist it was assessed whether it was reported in the main article or supplementary materials. The main analyses were based on items reported in either the main text or supplements. Each article was only assessed for items applicable to the study (i.e. development and/or external validation, or incremental value study). Scores for reporting level were calculated by assigning a single point for each reported item applicable to the study and total reporting level scores were converted to percentages based on the maximum possible score, and followed published scoring rules for the TRIPOD Adherence form.[16,17]

For peer review only - http://bmjopen.bmj.com/site/about/guidelines.xhtml

Assessment of study characteristics and used methods

In addition to the completeness of reporting following the TRIPOD statement, we assessed specific study characteristics and methods used in the included articles. To this end, we developed a comprehensive data extraction form based on previous studies, current methodological consensus, and the TRIPOD Exploration and Elaboration document (Supplementary text 1).[11,13,15,19–22] In summary the following topics were assessed: general study characteristics (i.e. diagnostic-prognostic and study topic), handling of missing data, model development methods, type of external validation and updating, and performance measures. To facilitate interpretation of the results section, main recommendations of the TRIPOD Exploration and Elaboration document are presented in Table 1. Assessment of these items was performed by two independent reviewers (AHZN and CLR) and a senior author (MvD) where necessary. In addition, for all articles published in the post-TRIPOD period, we extracted whether authors cited or referred to the TRIPOD Statement, provided the completed checklist, if the article was published in a journal that published the Statement (*The BMJ*, *Annals of Internal Medicine*, *BMC Medicine*), or was published in a journal that clearly stated in the author guidelines that they required TRIPOD adherence for submitted work at the time of writing this manuscript (*The BMJ, JAMA, and PLOS Medicine*). While all included journals (except for the *NEJM*) encouraged authors to follow the Equator Network guidelines, which includes the TRIPOD checklist, in their author instructions, only *The BMJ, JAMA and PLOS Medicine* required adherence to the Equator network guidelines and also required to include a filled-out checklist at the time of submission.

Analysis and reporting of results

Reporting levels are presented as percentages, stratified by journal, and for comparison the absolute difference in percentages with 95% confidence intervals are reported. Analyses were performed with IBM SPSS statistics (version 23.0, Armonk, NY, USA). Main results of the completeness of both reporting and methods are reported in text and detailed results are reported in the (Supplementary) Tables. Comparisons were made between articles I) Pre- and Post-TRIPOD, II) post-TRIPOD between articles published in journals that published and did not publish the TRIPOD, III) between articles published in journals that require TRIPOD adherence or not, IV) citing vs. not citing the TRIPOD, and V) providing vs. not providing a completed TRIPOD checklist. Furthermore, to estimate changes over time regardless of the TRIPOD statement, a comparison was made between pre-TRIPOD articles and post-TRIPOD articles not citing the TRIPOD.

For peer review only - http://bmjopen.bmj.com/site/about/guidelines.xhtml

RESULTS

Characteristics of included studies

The PubMed search string retrieved 481 articles, of which the full-text of 119 were read and 70 met our inclusion criteria (pre-TRIPOD: 32 articles, post-TRIPOD: 38 articles, Figure 1, Supplementary Text 1). Most of the included articles were published in *The BMJ* (n=38), and least in *The Lancet* (n=3) and NEJM (n=1). In both the pre- and post-TRIPOD period the majority of articles described prognostic models (as opposed to diagnostic models) and this increased in the post-TRIPOD period (pre-TRIPOD: 59%, post-TRIPOD: 89%) (Table 2). In the post-TRIPOD period the percentage of articles describing both the development and validation of a model (pre-TRIPOD: 31%, post-TRIPOD: 39%) or solely the external validation (pre-TRIPOD: 13%, post-TRIPOD: 26%) increased too. Thirty-two percent of articles only described the development of a prediction model without external validation in the post-TRIPOD period, compared to 44% in the pre-TRIPOD period. The majority of models were developed and/or validated using data from observational cohorts (pre-TRIPOD: 81%, post-TRIPOD: 82%) compared to other study designs such as randomised trials. More than half of the articles published in the post-TRIPOD period referred to the TRIPOD Statement (n=20, 53%) and were published in journals that published the TRIPOD Statement (n=21, 55%). The TRIPOD Statement was cited in 48% of articles published in journals that published the TRIPOD, and in 59% of articles in journals that did not publish TRIPOD.

Assessment of adherence to TRIPOD statement

Using the 2018 TRIPOD Adherence assessment form, a minimal non-significant increase in the overall percentage of reported items was found comparing the pre-TRIPOD period (74%) with the post-TRIPOD period (76%, absolute difference 2%, 95% CI: -4% to 7%, Figure 2, Supplementary Table 1), with no clear trend over the years (Supplementary Figure 1). Results were similar for the comparison between pre-TRIPOD articles and post-TRIPOD articles not citing the statement (76%, absolute difference 2%, 95%CI: -5% to 9%). An improvement for 16 of the individual TRIPOD items (44% of items, Supplementary Table 2) was seen, while 3 (8%) of items showed no improvement and 17 (47%) items showed a decrease in the percentage of articles appropriately reporting the item. Post-TRIPOD, for articles referring vs. not referring to the statement, published in journals that published vs. did not publish the statement, and published in journals that required adherence to the statement vs. did not require adherence to the statement, no difference in the completeness of reporting was observed (Supplementary Tables 3-5). Five articles presented the completed TRIPOD checklist in the supplementary material and the overall percentage of reporting for these articles was 80%. The percentage of articles reporting TRIPOD items in their supplement is presented in Supplementary Table 6.

Assessment of specific TRIPOD items

Abstract

In both the pre-TRIPOD (16%) and post-TRIPOD period (8%), most abstracts did not report all the proposed sub-items (TRIPOD item 2).

Reporting of missing data

In general, the reporting of missing data (TRIPOD item 13b) improved from 59% in the pre-TRIPOD period to

For peer review only - http://bmjopen.bmj.com/site/about/guidelines.xhtml

71% in the post-TRIPOD period, though fewer studies reported missingness per predictor in the post-TRIPOD period (pre-TRIPOD: 53%, post-TRIPOD: 37%, Figure 3 and Supplementary Table 7). Most studies did not report the reason for missing data (pre-TRIPOD: 84%, post-TRIPOD: 95%).

Model development and presentation

In the post-TRIPOD period, proper description of the characteristics of study participants (TRIPOD item 13b) was less often reported (37%) than in the pre-TRIPOD period (50%). In the post-TRIPOD period, method of predictor selection (TRIPOD-item 10b) was more often reported (70%) than in the pre-TRIPOD period (62%), as was internal validation (TRIPOD-item 10b) of the developed model (pre-TRIPOD 62%, post-TRIPOD 74%). If performed, unadjusted analyses were less often reported (TRIPOD item 14b) in the post-TRIPOD period (64%) than in the pre-TRIPOD period (86%). In the post-TRIPOD period, the full model (i.e. intercept or baseline hazard and all regression coefficients: TRIPOD-item 15a) was presented more frequently (41%), compared with the pre-TRIPOD period (29%). However, in both eras some studies still reported no information at all on the final model (pre-TRIPOD 8%; post-TRIPOD 4%, Figure 3 and Supplementary Table 8). To improve clinical usability (TRIPOD-item 15b), more than one third of studies reported to have developed a web application (pre-TRIPOD: 38%, post-TRIPOD: 37%) and some studies provided a simplified clinical risk score or nomogram (pre-TRIPOD: 29%; post-TRIPOD: 26%).

Performance measures

The percentage of studies reporting calibration (TRIPOD-item 16) of the model increased from 66% in the pre-TRIPOD period to 87% in the post-TRIPOD period. Discrimination (TRIPOD-item 16), was reported by all studies in the post-TRIPOD period and by 91% of studies in the pre-TRIPOD period. Measures of classification were reported less frequently in the post-TRIPOD period (pre-TRIPOD: 69%, post-TRIPOD: 58%). Measures of clinical usefulness like decision curve analysis were only reported by 2 (6%) studies in the pre-TRIPOD period and 7 (21%) studies in the post-TRIPOD period. Measures of overall performance like the Brier score or R^2 were infrequently reported in both periods (pre-TRIPOD: 19%, post-TRIPOD: 21%). Detailed results are depicted in Supplementary Table 7.

Assessment of methods

Handling of missing data

Multiple imputation was the most frequently performed approach for handling missing data (pre-TRIPOD: 38%, post-TRIPOD: 50%). The number of studies that used a complete case analysis remained constant and was 16% in both the pre- and post-TRIPOD period.

Model development

Post-TRIPOD, the number of studies that included predictors based on significance levels in univariable analysis decreased (pre-TRIPOD: 67%, post-TRIPOD: 44%, Figure 2 and Supplementary Table 8) as well as the number of studies using stepwise methods to retain predictors (pre-TRIPOD: 63%, post-TRIPOD: 48%). In general, a larger number of candidate predictors was used in the post-TRIPOD period (median: 25), compared with pre-TRIPOD period (median: 20). Internal validation was more frequently performed in the post-TRIPOD period (74%) compared with the pre-TRIPOD period (62%). When internal validation was performed,

For peer review only

For peer review only - http://bmjopen.bmj.com/site/about/guidelines.xhtml

bootstrapping was the most frequently used method in both time periods with an increase from 29% in the pre-TRIPOD period to 41% in the post-TRIPOD period.

Performance measures

The majority of studies presented measures of calibration (pre-TRIPOD: 66%, post-TRIPOD: 87%) and discrimination (pre-TRIPOD: 91%, post-TRIPOD: 100%, Figure 3 and Supplementary Table 7).A calibration plot and this increased in the post-TRIPOD period (pre-TRIPOD: 50%, post-TRIPOD: 82%)). Discrimination was primarily assessed with the C-statistic and Area Under the Curve (AUC) methods (pre-TRIPOD: 91%, post-TRIPOD: 100%). Measures of classification were reported in more than half of the studies (pre-TRIPOD: 69%, post-TRIPOD: 58%),mostly assessed with diagnostic test summary statistics (i.e. sensitivity, specificity and positive and negative predictive values) (pre-TRIPOD: 63%, post-TRIPOD: 50%) and to a lesser extent the integrated discrimination improvement (IDI; pre-TRIPOD: 16%, post-TIRPOD: 11%) or the net reclassification improvement (NRI; pre-TRIPOD 25%, post-TRIPOD: 18%).

External validation and model updating

Most external validation studies performed the validation in individuals fully unrelated to the development cohort (pre-TRIPOD 78%, post-TRIPOD: 88%, Figure 3 and Supplementary Table 9). Models were updated with an additional predictor in 4 (13%) studies before the TRIPOD statement and in 1 (3%) study after the TRIPOD statement.

For peer review only - http://bmjopen.bmj.com/site/about/guidelines.xhtml

DISCUSSION

No significant improvement in the overall reporting quality of prediction models published in the seven general medicine journals with the highest impact factor was found in the post-TRIPOD period, according to the TRIPOD Adherence form. However, an improvement in general methodological conduct was found. Notably, more studies described external validation of a model, reported information on missing data, used multiple imputation methods instead of complete case analysis for handling of missing data, selected and maintained variables in multivariable models based on clinical relevance instead of statistical cut-offs, and assessed both discrimination and calibration measures. While improvement was found for almost half of the TRIPOD items, no improvement or a deterioration was found for the other half of the items.

Recommendations on reporting and methods

Though improvements over time in specific aspects of reporting and methods were apparent, there is room for further progress. While an increase in studies reporting the percentage of missing data in the post-TRIPOD period was observed, the amount of missingness was often not reported per predictor, yet this is important for the assessment of clinical usability of the model.[15] Multiple imputation was the most frequently performed method for handling missing data, which generally is the preferred approach.[23] Reporting of all coefficients of the final multivariable model and intercept, which is necessary for external validation and clinical use of models, increased over time.[22] Although widely discouraged, a number of studies in both the pre-TRIPOD and post-TRIPOD period included predictors in multivariable prediction models based on data-driven selection methods such as univariable significance and/or stepwise methods. Such methods increase the risk of overfitted and poorly calibrated models.[11,22–26] Instead, it is advised to select predictors based on clinical knowledge and previous literature.[27] While the percentage of studies that both developed and externally validated a model increased over time, still more than 30% of articles only described the development of a model. External validation in a fully independent cohort is strongly recommended, as model performance might significantly decrease in cohorts other than the development cohort.[28] Assessment of both calibration and discrimination also increased, which is necessary in order to judge a model's predictive accuracy. Calibration refers to the agreement between absolute predicted and observed outcomes and the majority of studies used the preferred calibration plot.[29] Discrimination, a relative measure on the ability to distinguish between patients with and without the outcome, was reported by almost all studies.[29]

Comparison with other reporting guidelines

A large number of reporting guidelines have been published for various study types.[19,30–33] Mixed results on the effect of these guidelines on the completeness of reporting have been found.[34–38] While an overall modest improvement in reporting was described for randomised controlled trials after publication of the CONSORT statement and by the STARD statement for diagnostic studies, no clear improvement was described for observational studies by the STROBE statement and prognostic marker studies by the REMARK guideline as described by the authors of these studies.[34–38] These findings pose the question how the introduction and publication of these guidelines can optimally impact the research field. For both the CONSORT and STARD statement, journals endorsing the statement showed a higher level of reporting compared with journals not endorsing these statements. Nevertheless, this was not found for the REMARK guideline, nor in our study for

For peer review only - http://bmjopen.bmj.com/site/about/guidelines.xhtml

the TRIPOD statement.[34,37,38] Evidence of a relation between citing the statement and reporting level is also limited, as no association between this was found for the STARD nor in our study.[38] Requiring authors to provide and publish the completed checklist might help to improve reporting levels, as we found that the small numbers of studies providing the checklist reported more items on average. Therefore, we do not only recommend journals to ask authors to submit the completed checklist upon submission, but also require authors to publish it as a supplement, and reviewers and editors to control the provided checklist. However, as endorsing, citing and providing the checklist seems to have only a small effect on the reporting quality, we believe it is even more important to train methodologists and clinicians to interpret and use the checklist. This is supported by the results that even studies that provided the completed checklist, still did not report all items of the TRIPOD statement in analysis of reporting. Documents such as the TRIPOD Exploration and Elaboration document facilitate proper interpretation, but we believe that the threshold to use this detailed document might be too high for the unexperienced researcher. Other possibilities to familiarize authors with the checklist should be explored, such as collaborative efforts of educational institutions and the TRIPOD committee to train researchers and clinicians. Online training courses might be of added value to reach a large target group.

Comparison with other reviews on the completeness of reporting and methodological conduct of prediction models

Previous studies, published between 2012 and 2014, concluded poor reporting and use of methods for prediction models.[11,13,20,21] Comparing our results with a study assessing reporting and methods of prediction studies published in 6 high impact general medicine journals in 2008, improvement since then is clear for both methods and reporting. Considering methods more studies are externally validated, compose calibration plots to assess calibration and use multiple imputation for handling missing data. Improvement in reporting is also apparent as more studies report calibration and discrimination measures. Furthermore, a recently published article assessed the reporting quality of prediction models published in 37 clinical domains in 2014 using the 2018 TRIPOD Adherence assessment form, which found similar results to our Pre-TRIPOD results.[17] As we only included articles published in high impact general medicine journals it is difficult to generalize these results to the entire medical academic research field. We could argue that the improvement we observed might be an overestimation if general medical journals adopted the TRIPOD guidelines and new methodological insights with more speed and rigour. However, the opposite might also be true as these high impact general medicine journals already had high methodological standards before the TRIPOD statement publication.[11,13,16,21,35,36,39,40]

Strengths and Limitations

A limitation of the current study is that the evaluation of studies was limited to the first two years after the TRIPOD statement publication. It may take some years before a reporting guideline is widely disseminated and accepted and the full impact is measurable. However, to somewhat overcome this problem we did not include any articles published in 2015, as the TRIPOD statement was published in January 2015 and we therefore saw this as a transition period. In addition, a previously published study on the effect of STARD found significant improvement within two years after publication.[38] Furthermore it is not possible to causally attribute the reported changes to the TRIPOD statement, as the results might be confounded by other developments in the last decade, such as publication of multiple series on the conduct of prediction models, publication of other

For peer review only - http://bmjopen.bmj.com/site/about/guidelines.xhtml

guidelines such as the REMARK guideline for tumor marker prognostic studies, and a general increase in the numbers of published prediction models.[41–43] One may also expect that authors who work in the field of prediction models are aware of the publication of the TRIPOD statement, especially those who publish in high impact general medicine journals. A strength of the study is that the actual used methods for the development, description, validation and updating of prediction models were also assessed. While reporting and used methods are inherently related, the focus is different. A poorly developed model may be described fully and transparently in a manuscript and score high on reporting quality and vice versa a well-developed model may have poor reporting.[16] Furthermore, we have facilitated comparison to future TRIPOD reviews by using the 2018 TRIPOD Adherence assessment form. Although both reporting and methods were comprehensively assessed, we might have missed interesting items for evaluation, especially as the field of prediction models is continuously developing. We also did not assess the risk of bias of the included studies with the PROBAST risk of bias assessment tool, as it would be not feasible to score the included articles according to the PROBAST, since to do so subject-specific knowledge is required and the included studies span a wide range of clinical subjects. Furthermore, as the PROBAST only gives suggestions for signalling questions and no scoring rules, it does not completely fit with the aim to assess the actual used methods of the included studies. Furthermore, it would have been of interest to compare articles published in journals that between January 2016 and December 2017 obligated authors to complete the TRIPOD checklist, however this information was not available.

Unanswered questions and future research

Future studies should focus on the long-term effects of the TRIPOD statement publication on reporting quality and methods, using the 2018 TRIPOD Adherence form to allow for comparisons over time using the same adherence assessment tool. In addition, effects of the statement should be assessed in different medical fields for which a pre-TRIPOD baseline measurement is already performed.[16] Earlier studies on the effect of other reporting guidelines showed that the effect of these guidelines may be smaller or larger in specific medical fields.[34,40]

A new emerging field is the development of prediction models using artificial intelligence, machine learning and deep learning methods. In addition, more often omics data is used as predictors for these models.[44] While these models have many similarities with traditional regression methods, they differ in some aspects and may require specific guidelines on reporting.[44,45] Accordingly, the TRIPOD-AI tool has recently been announced and is underway.[46] Similarly, reporting guidelines for prediction model impact studies are missing.

With the increasing number of reporting guidelines and lack of clear evidence that all guidelines improve reporting quality, research should be conducted to find methods to optimise the form, use and impact of these guidelines. With this in mind, there should also be focus on the overlap between different reporting guidelines. Prediction models can be reported following the TRIPOD statement, the STARD statement for diagnostic test accuracy studies, and REMARK for prognostic tumor marker studies. As an increasing amount of studies contain multiple goals, analyses and data sources, it may be difficult to adhere to all applicable and relevant guidelines within the maximum word count. This holds especially for the abstract section of articles.

Conclusion

For peer review only

No improvement was found comparing the post-TRIPOD period with the pre-TRIPOD period in the overall reporting quality of prediction models published in the seven general medicine journals with the highest impact factor. Comparison of articles published before the TRIPOD statement with non-TRIPOD citing articles published after the TRIPOD statement, yielded similar results as the main pre-post comparison, further suggesting a lack of direct impact of the TRIPOD statement on overall reporting levels. However improvement was found in various specific aspects methodological conduct. More studies described external model validations, reported information on missing data, used multiple imputation methods for handling of missing data, reported the full prediction model and reported information on performance measures. However, there is still room for improvement in both the reporting and used methods of these models, as prediction models are still erroneously developed and validated and many aspects remain poorly reported, hindering optimal use of these models in clinical decision making. Long-term effects of the TRIPOD statement publication should be evaluated in future studies, ideally using the same 2018 TRIPOD Adherence assessment form to allow for comparisons over time.

For peer review only - http://bmjopen.bmj.com/site/about/guidelines.xhtml

Contributorship: AHZN conceived the study. AHZN, MvD, CLR, and EWS developed the study design with input from FWD, PH, LH, KGMM, WCP, GSC. AHZN and CLR screened the literature and performed the data-extraction. AHZN performed the statistical analysis and wrote the first and successive drafts of the manuscript. AHZN, CLR, FWD, PH, LH, KGMM, WCP, GSC, EWS, MvD interpreted the data, critically revised the manuscript for important intellectual content, and approved the final version of the manuscript.

Funding: AHZN and CLR were supported by personal Leiden University Medical Center MD/PhD Scholarships. MvD was supported by a grant from the Dutch Kidney Foundation (16OKG12). GSC is supported by the NIHR Biomedical Research Centre, Oxford and Cancer Research UK programme grant (C49297/A29084).

Competing interests: GSC, KGMM, and EWS are members of the TRIPOD group. All authors have completed the ICMJE uniform disclosure form at http://www.icmje.org/coi_disclosure.pdf (available on request from the corresponding author) and declare no other support from any organisation for the submitted work than the grants reported in the funding section; no financial relationships with any organisations that might have an interest in the submitted work in the previous three years, no other relationships or activities that could appear to have influenced the submitted work.

Ethical approval: Not required

Data sharing: All datasets that were used are retrievable following the instruction of the original papers.

Transparency: The lead author (AHZ) affirms that this manuscript is an honest, accurate, and transparent account of the study being reported; that no important aspects of the study have been omitted; and that any discrepancies from the study as planned have been explained.

Patient and Public Involvement: It was not appropriate or possible to involve patients or the public in the design, or conduct, or reporting, or dissemination plans of our research.

Dissemination declaration: not applicable.

For peer review only

For peer review only - http://bmjopen.bmj.com/site/about/guidelines.xhtml

REFERENCES

1 Moons KGM, Royston P, Vergouwe Y, *et al.* Prognosis and prognostic research: what, why, and how? *Bmj* 2009;**338**:b375–b375. doi:10.1136/bmj.b375

2 Steyerberg EW, Vickers AJ, Cook NR, *et al.* Assessing the performance of prediction models : A framework for some traditional and novel measures. *Epidemiology* 2010;**21**:128–38. doi:10.1097/EDE.0b013e3181c30fb2.Assessing

3 Pencina MJ, Demler O V. Novel metrics for evaluating improvement in discrimination: net reclassification and integrated discrimination improvement for normal variables and nested models. *Stat Methods Med Res* 2012;**31**:101–13. doi:10.1002/sim.4348.Novel

4 Pencina MJ, Fine JP, D'Agostino RB. Discrimination slope and integrated discrimination improvement - properties, relationships and impact of calibration. *Stat Med* Published Online First: 2016. doi:10.1002/sim.7139

5 Camm AJ, Kirchhof P, Lip GYH, *et al.* Guidelines for the management of atrial fibrillation. *Eur Heart J* 2010;**31**:2369–429. doi:10.1093/eurheartj/ehq278

6 Ravdin PM, Siminoff L a, Davis GJ, *et al.* Computer program to assist in making decisions about adjuvant therapy for women with early breast cancer. *J Clin Oncol* 2001;**19**:980–91. doi:10.1200/jco.2001.19.4.980

7 Stiell IG, Greenberg GH, McKnight RD, *et al.* A study to develop clinical decision rules for the use of radiography in acute ankle injuries. *Ann Emerg Med* 1992;**21**:384–90. doi:10.1016/S0196-0644(05)82656-3

8 Tesdale G, Jennet B. Assessment of coma and impaired consciousness. *Lancet* 1974;**13**:81–4.

9 Apgar V. Proposal for new method of evaluation of newborn infant. *Anesth Analg* 1953;**32**:260–7.

10 Wilson PWF, D'Agostino RB, Levy D, *et al.* Prediction of Coronary Heart Disease Using Risk Factor Categories. *Circulation* 1998;**97**:1837–47. doi:10.1161/01.CIR.97.18.1837

11 Bouwmeester W, Zuithoff NPA, Mallett S, *et al.* Reporting and methods in clinical prediction research: A systematic review. *PLoS Med* 2012;**9**:e1001221. doi:10.1371/journal.pmed.1001221

12 Mallett S, Royston P, Waters R, *et al.* Reporting performance of prognostic models in cancer: a review. *BMC Med* 2010;**8**:21. doi:1741-7015-8-21 [pii] 10.1186/1741-7015-8-21

13 Collins GS, Groot JA De, Dutton S, *et al.* External validation of multivariable prediction models : a systematic review of methodological conduct and reporting. *BMC Med Res Methodol* 2014;**14**:1–11.

14 Collins GS, Reitsma JB, Altman DG, *et al.* Transparent reporting of a multivariable prediction model for individual prognosis or diagnosis (TRIPOD): The TRIPOD Statement. *BMJ* 2014;**67**:1142–51. doi:10.1016/j.eururo.2014.11.025

15 Moons KGM, Altman DG, Reitsma JB, *et al.* Transparent Reporting of a multivariable prediction model for Individual Prognosis Or Diagnosis (TRIPOD): Explanation and Elaboration. *Ann Intern Med* 2015;**162**:W1–73. doi:10.7326/M14-0698

16 Heus P, Damen JAAG, Pajouheshnia R, *et al.* Poor reporting of multivariable prediction model studies: towards a targeted implementation strategy of the TRIPOD statement. *BMC Med* 2018;**16**:120. doi:10.1186/s12916-018-1099-2

For peer review only

For peer review only - http://bmjopen.bmj.com/site/about/guidelines.xhtml

17 Heus P, Damen JAAG, Pajouheshnia R, *et al.* Uniformity in measuring adherence to reporting guidelines: The example of TRIPOD for assessing completeness of reporting of prediction model studies. *BMJ Open* 2019;**9**:1–6. doi:10.1136/bmjopen-2018-025611

18 TRIPOD Downloads. https://www.tripod-statement.org/Downloads#Checklist (accessed 11 Feb2020).

19 Collins GS, Reitsma JB, Altman DG, *et al.* Transparent reporting of a multivariable prediction model for individual prognosis or diagnosis (TRIPOD): The TRIPOD Statement. *BMC Med* 2015;**13**:1–10. doi:10.1016/j.eururo.2014.11.025

20 Collins GS, Omar O, Shanyinde M, *et al.* A systematic review finds prediction models for chronic kidney disease were poorly reported and often developed using inappropriate methods. *J Clin Epidemiol* 2013;**66**:268–77. doi:10.1016/j.jclinepi.2012.06.020

21 Collins GS, Mallett S, Omar O, *et al.* Developing risk prediction models for type 2 diabetes : a systematic review of methodology and reporting. *BMC Med* 2011;**9**:1–14.

22 Steyerberg EW. *Clinical prediction models: a practical approach to development, validation, and updating*. New York, NY, US: : Springer 2009.

23 Jr FEH, Lee KL, Mark DB. Tutorial in Biostatistics Multivariable Prognostic Models : Issues in Developing Models , Evaluating Assumptions and Adequacy , and Measuring and Reducing Errors. *Stat Med* 1996;**15**:361–87. doi:10.1002/(SICI)1097-0258(19960229)15:4<361::AID-SIM168>3.0.CO;2-4

24 Sun GW, Shook TL, Kay GL. Inappropriate use of bivariable analysis to screen risk factors for use in multivariable analysis. *J Clin Epidemiol* 1996;**49**:907–16. doi:10.1016/0895-4356(96)00025-X

25 Steyerberg EW, Harrell FE, Borsboom GJJ., *et al.* Internal validation of predictive models. *J Clin Epidemiol* 2001;**54**:774–81. doi:10.1016/S0895-4356(01)00341-9

26 Moons KGM, Kengne AP, Woodward M, *et al.* Risk prediction models: I. Development, internal validation, and assessing the incremental value of a new (bio)marker. *Heart* 2012;**98**:683–90. doi:10.1136/heartjnl-2011-301246

27 Steyerberg EW, Vergouwe Y. Towards better clinical prediction models: Seven steps for development and an ABCD for validation. *Eur Heart J* 2014;**35**:1925–31. doi:10.1093/eurheartj/ehu207

28 Moons KGM, Kengne AP, Grobbee DE, *et al.* Risk prediction models: II. External validation, model updating, and impact assessment. *Heart* 2012;**98**:691–8. doi:10.1136/heartjnl-2011-301247

29 Altman DG, Vergouwe Y, Royston P, *et al.* Prognosis and prognostic research: validating a prognostic model. *Bmj* 2009;**338**:b605–b605. doi:10.1136/bmj.b605

30 Von Elm E, Altman DG, Egger M, *et al.* The Strengthening the Reporting of Observational Studies in Epidemiology (STROBE) statement: Guidelines for reporting observational studies. *PLoS Med* 2007;**4**:1623–7. doi:10.1371/journal.pmed.0040296

31 Schulz KF, Altman DG, Moher D, *et al.* CONSORT 2010 Statement: updated guidelines for reporting parallel group randomised trials. *BMC Med* 2010;**8**:18. doi:10.1186/1741-7015-8-18

32 Bossuyt PM, Reitsma JB, Bruns DE, *et al.* The STARD statement for reporting studies of diagnostic accuracy: Explanation and elaboration. *Clin Chem* 2003;**49**:7–18. doi:10.1373/49.1.7

33 McShane LM, Altman DG, Sauerbrei W, *et al.* REporting recommendations for tumor MARKer prognostic studies (REMARK). *Breast Cancer Res Treat* 2006;**100**:229–35. doi:10.1007/s10549-006-9242-8

For peer review only

For peer review only - http://bmjopen.bmj.com/site/about/guidelines.xhtml

34 Moher D, Jones a, Lepage L. Use of the CONSORT statement and quality of reports of randomized trials: a comparative before-and-after evaluation. *JAMA* 2001;**285**:1992–5. doi:10.1001/jama.285.15.1992

35 Bastuji-Garin S, Sbidian E, Gaudy-Marqueste C, *et al.* Impact of STROBE Statement Publication on Quality of Observational Study Reporting: Interrupted Time Series versus Before-After Analysis. *PLoS One* 2013;**8**:2–9. doi:10.1371/journal.pone.0064733

36 Poorolajal J, Cheraghi Z, Irani AD, *et al.* Quality of Cohort Studies Reporting Post the Strengthening the Reporting of Observational Studies in Epidemiology (STROBE) Statement. *Epidemiol Health* 2011;**33**:e2011005. doi:10.4178/epih/e2011005

37 Sekula P, Mallett S, Altman DG, *et al.* Did the reporting of prognostic studies of tumour markers improve since the introduction of REMARK guideline ? A comparison of reporting in published articles. *PLoS One* 2017;:1–15.

38 Smidt N, Rutjes AWSWS, van der Windt DAWMWM, *et al.* The quality of diagnostic accuracy studies since the STARD statement. *Neurology* 2006;**67**. doi:10.1212/01.wnl.0000238386.41398.30

39 Moher D, Jones A, Lepage L, *et al.* Use of the CONSORT Statement and Quality of Reports of Randomized Trials. *Jama* 2001;**285**:1992. doi:10.1001/jama.285.15.1992

40 Kiehna EN, Starke RM, Pouratian N, *et al.* Standards for reporting randomized controlled trials in neurosurgery. *J Neurosurg* 2011;**114**:280–5. doi:10.3171/2010.8.JNS091770

41 Hemingway H, Croft P, Perel P, *et al.* Prognosis research strategy (PROGRESS) 1: A framework for researching clinical outcomes. *BMJ* 2013;**346**:1–11. doi:10.1136/bmj.e5595

42 Moons KGM, Altman DG, Vergouwe Y, *et al.* Prognosis and prognostic research: application and impact of prognostic models in clinical practice. *BMJ* 2009;**338**:b606.

43 Altman DG, McShane LM, Sauerbrei W, *et al.* Reporting recommendations for tumor marker prognostic studies (REMARK): Explanation and elaboration. *PLoS Med* 2012;**9**. doi:10.1371/journal.pmed.1001216

44 Shameer K, Johnson KW, Glicksberg BS, *et al.* Machine learning in cardiovascular medicine: are we there yet? *Heart* 2018;:heartjnl-2017-311198. doi:10.1136/heartjnl-2017-311198

45 Shameer K, Johnson KW, Glicksberg BS, *et al.* The whole is greater than the sum of its parts: combining classical statistical and machine intelligence methods in medicine. *Heart* 2018;**104**:1228–1228. doi:10.1136/heartjnl-2018-313377

46 Collins GS, Moons KGM. Reporting of artificial intelligence prediction models. *Lancet* 2019;**393**:1577–9. doi:10.1016/S0140-6736(19)30037-6

47 Janssen KJM, Donders ART, Harrell FE, *et al.* Missing covariate data in medical research: To impute is better than to ignore. *J Clin Epidemiol* 2010;**63**:721–7. doi:10.1016/j.jclinepi.2009.12.008

48 Sterne JAC, White IR, Carlin JB, *et al.* Multiple imputation for missing data in epidemiological and clinical research: potential and pitfalls. *BMJ* 2009;**338**. doi:10.1136/bmj.b2393

49 Donders ART, van der Heijden GJMG, Stijnen T, *et al.* Review: A gentle introduction to imputation of missing values. *J Clin Epidemiol* 2006;**59**:1087–91. doi:10.1016/j.jclinepi.2006.01.014

50 Steyerberg EW, Vickers AJ, Cook NR, *et al.* Assessing the Performance of Prediction Models. *Epidemiology* 2010;**21**:128–38. doi:10.1097/EDE.0b013e3181c30fb2

For peer review only

For peer review only - http://bmjopen.bmj.com/site/about/guidelines.xhtml

51 Debray TPA, Vergouwe Y, Koffijberg H, *et al.* A new framework to enhance the interpretation of external validation studies of clinical prediction models. *J Clin Epidemiol* 2015;**68**:279–89. doi:10.1016/j.jclinepi.2014.06.018

For peer review only

For peer review only - http://bmjopen.bmj.com/site/about/guidelines.xhtml

FIGURE CAPTIONS

Figure 1: Flow chart of search results and selection procedure

Figure 2: TRIPOD reporting scores

Figure 3: Comparison of used methods in the pre-TRIPOD and post-TRIPOD period

For peer review only

For peer review only - http://bmjopen.bmj.com/site/about/guidelines.xhtml

Table 1: Recommended methods and analyses for the development and validation of prediction models including supportive references

**Methodology**		
**Handling of missing data**	It is generally advised to use multiple imputation for handling of missing data. Complete case analysis, single or mean imputation are inefficient methods to estimate coefficients	[47–49]
**Selection and retaining of predictors in multivariable models**	Predictor selection and retaining is preferably based on clinical knowledge and previous literature, instead of significance levels in univariable or stepwise analysis.	[22,26,27]
**Internal validation**	It is advised to internally validate the model to assess optimism in performance and reduce over-fitting. An efficient method is bootstrapping; split-sample validation should be avoided.	[25,26]
**Calibration**	It is advised to assess the calibration of a model at external validation. The preferred method is a calibration plot, with intercept and slope, and not statistical tests (e.g. Hosmer-Lemeshow), as a plot retains the most information on possible miscalibration.	[22,26,27,50]
**External validation**	External validation of models is needed for rigorous assessment of performance. The preferred external validation population is fully independent.	[28,51]

For peer review only

Table 2: Characteristics of included studies

	Before 2015 (n=32) number, (%)	After 2015 (n=38) number, (%)
**Diagnostic/Prognostic**		
Diagnostic	13 (41%)	4 (11%)
Prognostic	19 (59%)	34 (89%)
**Type**		
Development	14 (44%)	12 (32%)
Validation	4 (13%)	10 (26%)
Development and Validation	10 (31%)	15 (39%)
Update	4 (13%)	1 (3%)
**Setting**		
General population and Primary care	18 (56%)	18 (47%)
Secondary care	14 (44%)	20 (53%)
**Design**		
Cohort	26 (81%)	31 (82%)
RCT	1 (30%)	4 (11%)
Cohort and RCT	2 (6%)	3 (8%)
Case-Control	3 (9%)	0 (0%)
**Topic**		
(Cardio)vascular	12 (38%)	16 (42%)
Oncological	3 (9%)	8 (21%)
Other	17 (53%)	14 (37%)

For peer review only

For peer review only - http://bmjopen.bmj.com/site/about/guidelines.xhtml

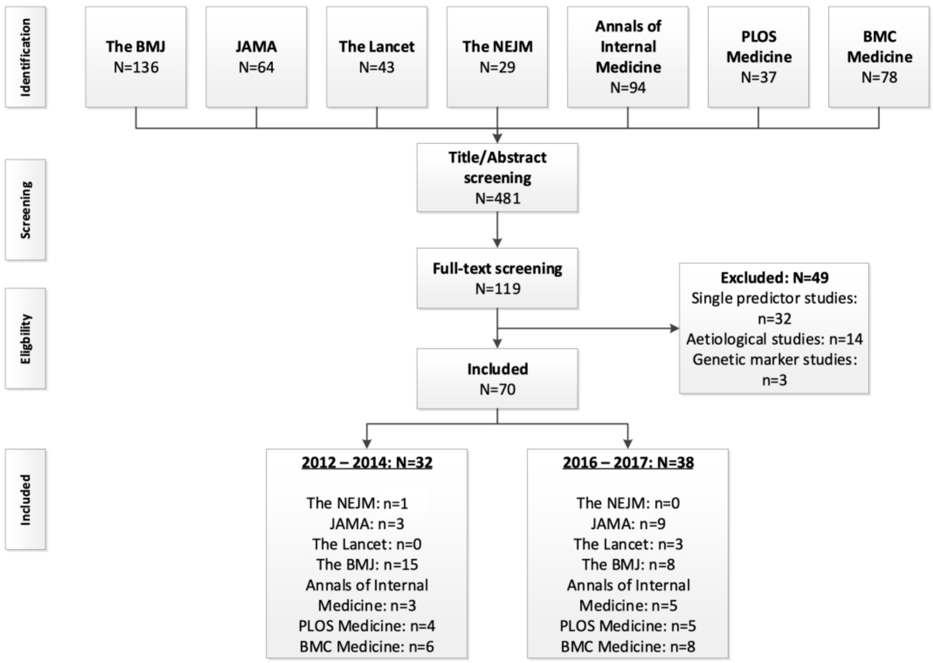

Figure 1: Flow chart of search results and selection procedure

253x190mm (300 x 300 DPI)

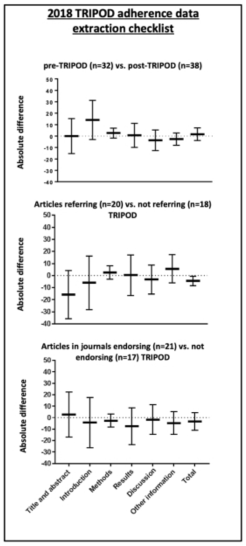

Figure 2: TRIPOD reporting scores

253x190mm (300 x 300 DPI)

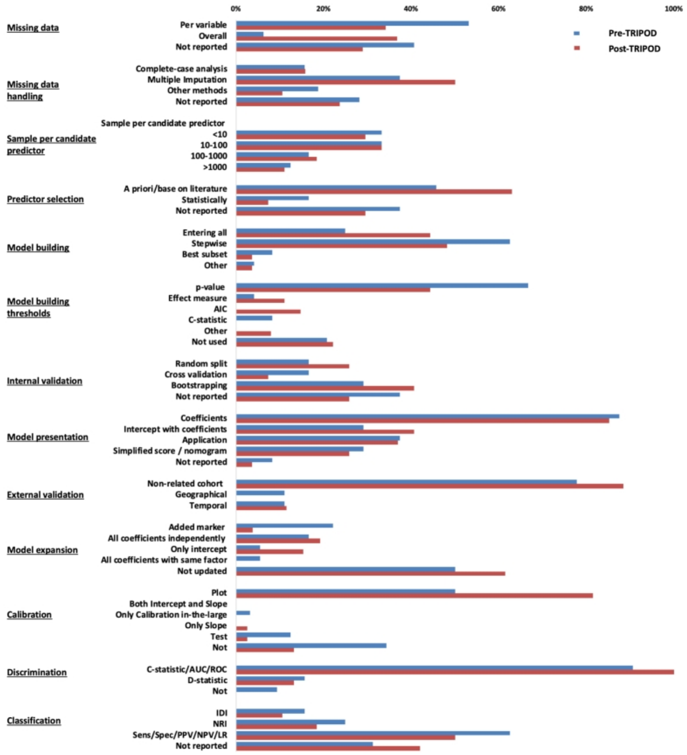

Figure 3: Comparison of used methods in the pre-TRIPOD and post-TRIPOD period

253x190mm (300 x 300 DPI)

Supplementary documents

The impact of the TRIPOD statement: a pre-post analysis of reporting and methods.

Amir H Zamanipoor Najafabadi BSc[1,2,3], Chava L Ramspek MD[3], Friedo W. Dekker[3], Pauline Heus MSc[4,5], Lotty Hooft PhD[4,5], Karel G M Moons MD PhD[4,5], Wilco C Peul MD PhD[1], Gary S Collins PhD[6,7], Ewout W Steyerberg PhD[8], Merel van Diepen PhD[3].

[1]*Department of Neurosurgery, University Neurosugrical Center Holland, Leiden University Medical Center & Haaglanden Medical Center & Haga Teaching Hospitals, Leiden & the Hageue, the Netherlands*
[2]*Department of Neurology, Leiden University Medical Center, Leiden, the Netherlands*
[3]*Department of Clinical Epidemiology, Leiden University Medical Center, Leiden, the Netherlands*
[4]*Cochrane Netherlands, University Medical Center Utrecht, Utrecht University*
[5]*Julius Center for Health Sciences and Primary Care, University Medical Center Utrecht, Utrecht University*
[6]*Centre for Statistics in Medicine, Nuffield Department of Orthopaedics, Rheumatology and Musculoskeletal Sciences, University of Oxford, Oxford, UK.*
[7]*National Institute for Health Research Oxford Biomedical Research Centre, John Radcliffe Hospital, Oxford, UK*
[8]*Department of Biomedical Data Sciences, Leiden University Medical Center, Leiden, The Netherlands.*

Corresponding author: Amir H. Zamanipoor Najafabadi, Department of Neurosurgery, Neurology and Clinical Epidemiology, Leiden University Medical Center, Leiden, the Netherlands. *Tel:* +31-(0)71-5262109. Fax: +31-(0)71-5266987. *Email:* a.h.zamanipoor_najafabadi@lumc.nl

For peer review only

Supplementary Table 1: TRIPOD reporting scores for all included articles

	Before % n=32	After % n=38	Absolute difference of percentages (95% CI)
**Title and abstract**	25	25	0 (-15 to 15)
**Introduction**	69	83	14 (-3 to 31)
**Methods**	80	83	3 (-2 to 7)
**Results**	66	67	1 (-10 to 11)
**Discussion**	89	85	-4 (-13 to 5)
**Other information**	100	97	-3 (-8 to 3)
**Total**	74	76	2 (-4 to 7)

Supplementary Table 2: TRIPOD reporting scores for individual TRIPOD items

TRIPOD item	pre-TRIPOD %	post-TRIPOD %
**1**	34	42
**2**	16	8
**3a**	78	84
**3b**	59	82
**4a**	97	100
**4b**	91	95
**5a**	97	95
**5b**	97	97
**5c**	100	60
**6a**	97	97
**6b**	97	95
**7a**	88	87
**7b**	94	100
**8**	100	100
**9**	28	24
**10a**	63	84
**10b**	19	39
**10c**	67	100
**10d**	69	71
**10e**	56	50
**11**	96	95
**12**	75	71
**13a**	94	95
**13b**	50	37
**13c**	53	65
**14a**	100	97
**14b**	86	64
**15a**	27	42
**15b**	65	61
**16**	66	68
**17**	40	73
**18**	94	97
**19a**	82	75
**19b**	100	95
**20**	75	68
**22**	100	97

For peer review only

Supplementary Table 3: TRIPOD reporting scores for articles published after TRIPOD statement referring vs not referring to the statement

	TRIPOD not referred % n=18	TRIPOD referred % n=20	Absolute difference of percentages (95% CI)
**Title and abstract**	33	18	-16 (-36 to 4)
**Introduction**	86	80	-6 (-28 to 16)
**Methods**	82	84	2 (-3 to 8)
**Results**	67	67	0 (-17 to 17)
**Discussion**	87	83	-3 (-15 to 9)
**Other information**	94	100	6 (-6 to 17)
**Total**	76	76	-1 (-8 to 7)

Supplementary Table 4: TRIPOD reporting scores for articles published after TRIPOD statement in journals that published and did not publish the TRIPOD statement

	TRIPOD not endorsed % n=17	TRIPOD endorsed % n=21	Absolute difference of percentages (95% CI)
**Title and abstract**	24	26	3 (-17 to 22)
**Introduction**	85	81	-4 (-26 to 18)
**Methods**	84	82	-3 (-8 to 3)
**Results**	71	63	-7 (-24 to 9)
**Discussion**	86	84	-2 (-15 to 11)
**Other information**	100	95	-5 (-15 to 5)
**Total**	78	75	-3 (-11 to 4)

Supplementary Table 5: TRIPOD reporting scores for articles published after TRIPOD statement in journals that require adherence to the TRIPOD statement and journals that do not require to the adherence statement

	TRIPOD not required % n=16	TRIPOD required % n=22	Absolute difference of percentages (95% CI)
**Title and abstract**	25	25	0 (-21 to 21)
**Introduction**	81	84	3 (-20 to 26)
**Methods**	83	83	0 (-6 to 6)
**Results**	66	67	1 (-16 to 19)
**Discussion**	90	81	-8 (-20 to 3)
**Other information**	100	95	-5 (-15 to 5)
**Total**	76	76	0 (-8 to 9)

For peer review only

Supplementary Table 6: Percentage articles reporting TRIPOD items in supplementary material

TRIPOD item	Supplement %
**1**	0
**2**	0
**3a**	0
**3b**	0
**4a**	7
**4b**	7
**5a**	10
**5b**	6
**5c**	33
**6a**	13
**6b**	0
**7a**	36
**7b**	0
**8**	3
**9**	9
**10a**	12
**10b**	13
**10c**	14
**10d**	1
**10e**	0
**11**	4
**12**	24
**13a**	21
**13b**	30
**3c**	17
**14a**	2
**14b**	40
**15a**	40
**15b**	35
**16**	0
**17**	17
**18**	0
**19a**	0
**19b**	0
**20**	0
**22**	1

Supplementary figure 1: Average overall TRIPOD reporting levels in percentage per year

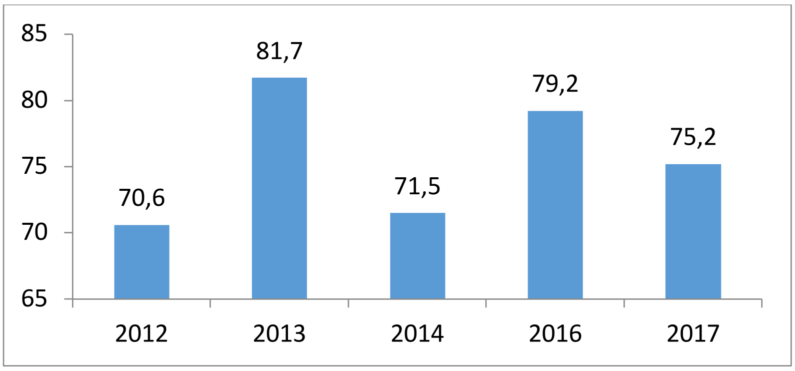

For peer review only

Supplementary Table 7: Performance measures and missing data in all studies

	Before 2015 (n=32) Number (%)	After 2016 (n=38) Number (%)
**Calibration**		
Plot	16 (50%)	31 (82%)
Intercept and Slope	0 (0%)	0 (0%)
Calibration in-the-large	1 (3%)	0 (0%)
Slope	0 (0%)	1 (3%)
Test	4 (13%)	1 (3%)
Not	11 (34%)	5 (13%)
**Discrimination**		
C-statistic / AUC	29 (91%)	38 (100%)
D-statistic	5 (16%)	5 (13%)
Not	3 (9%)	0 (0%)
**Classification**		
IDI	5 (16%)	4 (11%)
NRI	8 (25%)	7 (18%)
Sens. Spec. PPV. NPV. LR. ROC	20 (63%)	19 (50%)
Not reported	10 (31%)	16 (42%)
**Clinical usefulness**		
Decision curve analysis	2 (6%)	8 (21%)
Not reported	30 (94%)	30 (79%)
**Overall performance**		
Brier	2 (6%)	3 (8%)
R2	4 (13%)	5 (13%)
Adequacy statistic	1 (3%)	2 (5%)
Not reported	26 (81%)	28 (74%)
**Missing data reporting**		
Per variable	17 (53%)	13 (34%)
Overall	2 (6%)	14 (37%)
Not reported	13 (41%)	11 (29%)
**Type and reason of missing data**		
Type reported	3 (9%)	2 (5%)
Reason reported	5 (16%)	2 (5%)
**Missing data handling**		
Complete-case analysis	5 (16%)	6 (16%)
Multiple Imputation	12 (38%)	19 (50%)
Other methods	6 (19%)	4 (11%)
Not reported	9 (28%)	9 (24%)

For peer review only

Supplementary Table 8: Model development and presentation

	Before 2015 (n=24) Number (%)	After 2016 (n=27) Number (%)
**Sample per candidate predictor**		
<10	8 (33%)	8 (30%)
10-100	8 (33%)	9 (33%)
100-1000	8 (17%)	5 (19%)
>1000	3 (13%)	3 (11%)
Unknown number of predictors	0 (0%)	1 (4%)
Unknown number of outcomes	1 (4%)	1 (4%)
**Model Type**		
Linear	1 (4%)	0 (0%)
Logistic	16 (67%)	15 (56%)
Cox	6 (25%)	10 (37%)
Points	0 (0%)	2 (7%)
Other	1 (4%)	0 (0%)
**Predictor Selection**		
A priori knowledge / based on literature	11 (46%)	17 (63%)
Statistically	4 (17%)	2 (7%)
Not reported	9 (38%)	8 (30%)
**Model building**		
Entering all	6 (25%)	12 (44%)
Stepwise	15 (63%)	13 (48%)
Best subset	2 (8%)	1 (4%)
Other	1 (4%)	1 (4%)
**Model building thresholds**		
p-value	16 (67%)	12 (44%)
Effect measure	1 (4%)	3 (11%)
R	0 (0%)	1 (4%)
Manually	0 (0%)	1 (4%)
AIC	0 (0%)	4 (15%)
C-statistic	2 (8%)	0 (0%)
Other	0 (0%)	0 (0%)
Not used	5 (21%)	6 (22%)
**Internal validation**		
Random split	4 (17%)	7 (26%)
Cross validation	4 (17%)	2 (7%)
Bootstrapping	7 (29%)	11 (41%)
Not reported	9 (38%)	7 (26%)
**Model presentation**		
Coefficients	21 (88%)	23 (85%)
Intercept with coefficients	7 (29%)	11 (41%)
Application	9 (38%)	10 (37%)
Simplified score / nomogram	7 (29%)	7 (26%)
Not reported	2 (8%)	1 (4%)

For peer review only

1 **Supplementary Table 9: External validation and updating**

	Before 2015 (n=18) Number (%)	After 2016 (n=26) Number (%)
**External validation**		
Fully independent	14 (78%)	23 (88%)
Geographical	2 (11%)	0 (0%)
Temporal	2 (11%)	3 (12%)
**Model updating**		
Added marker	4 (22%)	1 (4%)
All coefficients independently	3 (17%)	5 (19%)
Only intercept	1 (6%)	4 (15%)
All coefficients with same factor	1 (6%)	0 (0%)
Not updated	9 (50%)	16 (62%)

2
3

For peer review only

Supplementary text 1: Protocol

Article selection

Search strategy:

("The New England journal of medicine"[Journal] OR "Lancet (London, England)"[Journal] OR "BMJ (Clinical research ed.)"[Journal] OR "*JAMA*"[Journal] OR "PLOS Medicine"[Journal] OR "*Annals of Internal Medicine*" [Journal] OR "*BMC Medicine*"[Journal]) AND (predict*[ti] OR prognost*[ti] OR diagnostic*[ti] OR "risk score"[ti]) NOT ("Animals"[mesh] NOT "Humans"[mesh]) NOT (("case reports"[ptyp] OR "case report"[ti]) NOT ("Review"[ptyp] OR "clinical study"[ptyp] OR "case series"[tw]))

Articles included

1 Abbasi A, Peelen LM, Corpeleijn E, *et al.* Prediction models for risk of developing type 2 diabetes: Systematic literature search and independent external validation study. *BMJ* 2012;**345**:1–16. doi:10.1136/bmj.e5900

2 Bejnordi BE, Veta M, Van Diest PJ, *et al.* Diagnostic assessment of deep learning algorithms for detection of lymph node metastases in women with breast cancer. *JAMA - J Am Med Assoc* 2017;**318**:2199–210. doi:10.1001/jama.2017.14585

3 Collins GS, Altman DG. Predicting the 10 year risk of cardiovascular disease in the United Kingdom: Independent and external validation of an updated version of QRISK2. *BMJ* 2012;**345**:1–12. doi:10.1136/bmj.e4181

4 Costa F, van Klaveren D, James S, *et al.* Derivation and validation of the predicting bleeding complications in patients undergoing stent implantation and subsequent dual antiplatelet therapy (PRECISE-DAPT) score: a pooled analysis of individual-patient datasets from clinical trials. *Lancet* 2017;**389**:1025–34. doi:10.1016/S0140-6736(17)30397-5

5 Cubiella J, Vega P, Salve M, *et al.* Development and external validation of a faecal immunochemical test-based prediction model for colorectal cancer detection in symptomatic patients. *BMC Med* 2016;**14**:1–13. doi:10.1186/s12916-016-0668-5

6 D.S. L, A. S, P.C. A, *et al.* Prediction of heart failure mortality in emergent care: A cohort study. *Ann Intern Med* 2012;**156**:767–75. doi:10.7326/0003-4819-156-11-201206050-00003

7 Dagan N, Cohen-Stavi C, Leventer-Roberts M, *et al.* External validation and comparison of three prediction tools for risk of osteoporotic fractures using data from population based electronic health records: Retrospective cohort study. *BMJ* 2017;**356**. doi:10.1136/bmj.i6755

8 Dalton JE, Perzynski AT, Zidar DA, *et al.* Accuracy of cardiovascular risk prediction varies by neighborhood socioeconomic position a retrospective cohort study. *Ann Intern Med* 2017;**167**:456–64. doi:10.7326/M16-2543

9 Dalziel SR, Thompson JMD, Macias CG, *et al.* Predictors of severe H1N1 infection in children presenting within Pediatric Emergency Research Networks (PERN): Retrospective case-control study. *BMJ* 2013;**347**:1–13. doi:10.1136/bmj.f4836

10 Terfc. Glycated hemoglobin measurement and prediction of cardiovascular disease. *JAMA - J Am Med Assoc* 2014;**311**:1225–33. doi:10.1001/jama.2014.1873

11 Elias SG, Kok L, de Wit NJ, *et al.* Is there an added value of faecal calprotectin and haemoglobin in the diagnostic work-up for primary care patients suspected of significant colorectal disease? A cross-sectional diagnostic study. *BMC Med* 2016;**14**:1–11. doi:10.1186/s12916-016-0684-5

12 Kaptoge. C-Reactive Protein, Fibrinogen, and Cardiovascular Disease Prediction. *N Engl J Med* 2012;**367**:1310–20. doi:10.1056/NEJMoa1107477

13 Esplin MS, Elovitz MA, Iams JD, *et al.* Predictive accuracy of serial transvaginal cervical lengths and quantitative vaginal fetal fibronectin levels for spontaneous preterm birth among nulliparous women. *JAMA - J Am Med Assoc* 2017;**317**:1047–56. doi:10.1001/jama.2017.1373

For peer review only

14 Fischer K, Kettunen J, Würtz P, *et al.* Biomarker Profiling by Nuclear Magnetic Resonance Spectroscopy for the Prediction of All-Cause Mortality: An Observational Study of 17,345 Persons. *PLoS Med* 2014;**11**. doi:10.1371/journal.pmed.1001606

15 Fraccaro P, van der Veer S, Brown B, *et al.* An external validation of models to predict the onset of chronic kidney disease using population-based electronic health records from Salford, UK. *BMC Med* 2016;**14**:1–15. doi:10.1186/s12916-016-0650-2

16 Freund Y, Lemachatti N, Krastinova E, *et al.* Prognostic accuracy of sepsis-3 criteria for in-hospital mortality among patients with suspected infection presenting to the emergency department. *JAMA - J Am Med Assoc* 2017;**317**:301–8. doi:10.1001/jama.2016.20329

17 Ganz P, Heidecker B, Hveem K, *et al.* Development and validation of a protein-based risk score for cardiovascular outcomes among patients with stable coronary heart disease. *JAMA - J Am Med Assoc* 2016;**315**:2532–41. doi:10.1001/jama.2016.5951

18 Genders TSS, Steyerberg EW, Hunink MGM, *et al.* Prediction model to estimate presence of coronary artery disease: Retrospective pooled analysis of existing cohorts. *BMJ* 2012;**344**:1–13. doi:10.1136/bmj.e3485

19 Gnanapragasam VJ, Lophatananon A, Wright KA, *et al.* Improving Clinical Risk Stratification at Diagnosis in Primary Prostate Cancer: A Prognostic Modelling Study. *PLoS Med* 2016;**13**:1–18. doi:10.1371/journal.pmed.1002063

20 Goldstick JE, Carter PM, Walton MA, *et al.* Development of the SaFETy score: A clinical screening tool for predicting future firearm violence risk. *Ann Intern Med* 2017;**166**:707–14. doi:10.7326/M16-1927

21 Goodacre S, Wilson R, Shephard N, *et al.* Derivation and validation of a risk adjustment model for predicting seven day mortality in emergency medical admissions: Mixed prospective and retrospective cohort study. *BMJ* 2012;**344**:1–11. doi:10.1136/bmj.e2904

22 den ruitjer H, Rembold CM. Common Carotid Intima-Media Thickness Measurements in Cardiovascular Risk Prediction. *J Am Med Assoc* 2015;**308**:796–803.http://jama.jamanetwork.com/

23 Hijazi Z, Oldgren J, Lindbäck J, *et al.* The novel biomarker-based ABC (age, biomarkers, clinical history)-bleeding risk score for patients with atrial fibrillation: a derivation and validation study. *Lancet* 2016;**387**:2302–11. doi:10.1016/S0140-6736(16)00741-8

24 Hippisley-Cox J, Coupland C. Development and validation of risk prediction equations to estimate survival in patients with colorectal cancer: Cohort study. *BMJ* 2017;**357**. doi:10.1136/bmj.j2497

25 Hippisley-Cox J, Coupland C, Brindle P. Development and validation of QRISK3 risk prediction algorithms to estimate future risk of cardiovascular disease: Prospective cohort study. *BMJ* 2017;**357**. doi:10.1136/bmj.j2099

26 Hippisley-Cox J. Predicting risk of upper gastrointestinal bleed and intracranial bleed with anticoagulants: Cohort study to derive and validate the QBleed scores. *BMJ* 2014;**349**:1–21. doi:10.1136/bmj.g4606

27 Hippisley-Cox J, Coupland C, Brindle P. Derivation and validation of QStroke score for predicting risk of ischaemic stroke in primary care and comparison with other risk scores: A prospective open cohort study. *BMJ* 2013;**346**:1–15. doi:10.1136/bmj.f2573

28 Hippisley-Cox J, Coupland C. Derivation and validation of updated QFracture algorithm to predict risk of osteoporotic fracture in primary care in the United Kingdom: Prospective open cohort study. *BMJ* 2012;**345**:1–16. doi:10.1136/bmj.e3427

29 Hippisley-Cox J, Coupland C. Development and validation of QMortality risk prediction algorithm to estimate short term risk of death and assess frailty: cohort study. *BMJ* 2017;**358**:j4208. doi:10.1136/bmj.j4208

30 J.M. Q, C. EE, A. U, *et al.* Predictive score for mortality in patients with COPD exacerbations attending hospital emergency departments. *BMC Med* 2014;**12**:66. doi:10.1186/1741-7015-12-66

31 James MT, Pannu N, Hemmelgarn BR, *et al.* Derivation and external validation of prediction models for advanced chronic kidney disease following acute kidney injury. *JAMA - J Am Med Assoc* 2017;**318**:1787–97. doi:10.1001/jama.2017.16326

32 Kavousi M, Desai CS, Ayers C, *et al.* Prevalence and prognostic implications of coronary artery calcification in low-risk women: A meta-analysis. *JAMA - J Am Med Assoc* 2016;**316**:2126–34.

For peer review only

doi:10.1001/jama.2016.17020
33 Kent M, Penson DF, Albertsen PC, *et al.* Successful external validation of a model to predict other cause mortality in localized prostate cancer. *BMC Med* 2016;**14**:1–7. doi:10.1186/s12916-016-0572-z
34 Perel P. Predicting early death in patients with traumatic bleeding: development and validation of prognostic model. *J Geophys Res* 1989;**94**:3579. doi:10.1136/bmj.e5166
35 Kugathasan S, Denson LA, Walters TD, *et al.* Prediction of complicated disease course for children newly diagnosed with Crohn's disease: a multicentre inception cohort study. *Lancet* 2017;**389**:1710–8. doi:10.1016/S0140-6736(17)30317-3
36 Lamain-de Ruiter M, Kwee A, Naaktgeboren CA, *et al.* External validation of prognostic models to predict risk of gestational diabetes mellitus in one Dutch cohort: prospective multicentre cohort study. *BMJ* 2016;**354**:i4338. doi:10.1136/bmj.i4338
37 Little P, Stuart B, Richard Hobbs FD, *et al.* Predictors of suppurative complications for acute sore throat in primary care: Prospective clinical cohort study. *BMJ* 2013;**347**:1–14. doi:10.1136/bmj.f6867
38 Wang s w. Anti-allergic activity of some selected plants in the genus Boesenbergia and Kaempferia. *Songklanakarin J Sci Technol* 2011;**33**:301–4. doi:10.1136/bmj.i2607
39 Dewland TA. Atrial Ectopy as a Predictor of Incident Atrial Fibrillation. 2011;**4**:721–8. doi:10.1126/scisignal.2001449.Engineering
40 Marcantonio ER, Ngo LH, O'Connor M, *et al.* 3D-CAM: Derivation and validation of a 3-minute diagnostic interview for CAM-defined delirium: A cross-sectional diagnostic test study. *Ann Intern Med* 2014;**161**:554–61. doi:10.7326/M14-0865
41 Matsushita K, Woodward M, Jafar TH, *et al.* Comparison of Risk Prediction Using the CKD-EPI Equation and the MDRD Study Equation for Estimated Glomerular Filtration Rate. 2012;**307**:1941–51.
42 McLernon DJ, Steyerberg EW, Te Velde ER, *et al.* Predicting the chances of a live birth after one or more complete cycles of in vitro fertilisation: Population based study of linked cycle data from 113 873 women. *BMJ* 2016;**355**. doi:10.1136/bmj.i5735
43 Miro O, Rossello X, Gil V, *et al.* Predicting 30-day mortality for patients with acute heart failure in the emergency department. *Ann Intern Med* 2017;**167**:698–705. doi:10.7326/M16-2726
44 Moore CL, Bomann S, Daniels B, *et al.* Derivation and validation of a clinical prediction rule for uncomplicated ureteral stone-the STONE score: Retrospective and prospective observational cohort studies. *BMJ* 2014;**348**:1–12. doi:10.1136/bmj.g2191
45 Mwangi MN, Maskey S, Andang'o PEA, *et al.* Diagnostic utility of zinc protoporphyrin to detect iron deficiency in Kenyan pregnant women. *BMC Med* 2014;**12**:1–13. doi:10.1186/s12916-014-0229-8
46 Nijman RG, Vergouwe Y, Thompson M, *et al.* Clinical prediction model to aid emergency doctors managing febrile children at risk of serious bacterial infections: Diagnostic study. *BMJ* 2013;**346**:1–16. doi:10.1136/bmj.f1706
47 Koller MT. Original Research Older U . S . and European Persons in the Cardiovascular Health Study. *Ann Intern Med* 2012.
48 Patel VB, Theron G, Lenders L, *et al.* Diagnostic Accuracy of Quantitative PCR (Xpert MTB/RIF) for Tuberculous Meningitis in a High Burden Setting: A Prospective Study. *PLoS Med* 2013;**10**. doi:10.1371/journal.pmed.1001536
49 Pavese C, Schneider MP, Schubert M, *et al.* Prediction of Bladder Outcomes after Traumatic Spinal Cord Injury: A Longitudinal Cohort Study. *PLoS Med* 2016;**13**:1–16. doi:10.1371/journal.pmed.1002041
50 Payne BA, Hutcheon JA, Ansermino JM, *et al.* A Risk Prediction Model for the Assessment and Triage of Women with Hypertensive Disorders of Pregnancy in Low-Resourced Settings: The miniPIERS (Pre-eclampsia Integrated Estimate of RiSk) Multi-country Prospective Cohort Study. *PLoS Med* 2014;**11**. doi:10.1371/journal.pmed.1001589
51 Pfeiffer RM, Park Y, Kreimer AR, *et al.* Risk Prediction for Breast, Endometrial, and Ovarian Cancer in White Women Aged 50 y or Older: Derivation and Validation from Population-Based Cohort Studies. *PLoS Med* 2013;**10**. doi:10.1371/journal.pmed.1001492

For peer review only

52 Q. L, M. IDK, T.K. T, *et al.* A clinical prediction rule for diagnosing human infections with avian influenza A(H7N9) in a hospital emergency department setting. *BMC Med* 2014;**12**:1–9. doi:10.1186/s12916-014-0127-0 LK - http://sfx.hul.harvard.edu/sfx_local?sid=EMBASE&issn=17417015&id=doi:10.1186%2Fs12916-014-0127-0&atitle=A+clinical+prediction+rule+for+diagnosing+human+infections+with+avian+influenza+A%28H7N9%29+in+a+hospital+emergency+department+setting&stitle=BMC+Med.&title=BMC+Medicine&volume=12&issue=1&spage=&epage=&aulast=Liao&aufirst=Qiaohong&auinit=Q.&aufull=Liao+Q.&coden=&isbn=&pages=-&date=2014&auinit1=Q&auinitm=

53 Raith EP, Udy AA, Bailey M, *et al.* Prognostic accuracy of the SOFA score, SIRS criteria, and qSOFA score for in-hospital mortality among adults with suspected infection admitted to the intensive care unit. *JAMA - J Am Med Assoc* 2017;**317**:290–300. doi:10.1001/jama.2016.20328

54 Pandie S. Diagnostic accuracy of quantitative PCR (Xpert MTB/RIF) for tuberculous pericarditis compared to adenosine deaminase and unstimulated interferon-γ in a high burden setting: A prospective study. *BMC Med* 2014;**12**:2–11. doi:10.1186/1741-7015-12-101

55 Raji O y. Annals of Internal Medicine Predictive Accuracy of the Liverpool Lung Project Risk Model for Stratifying Patients for Computed Tomography Screening for Lung Cancer. 2012.

56 Sultan AA, West J, Grainge MJ, *et al.* Development and validation of risk prediction model for venous thromboembolism in postpartum women: Multinational cohort study. *BMJ* 2016;**355**. doi:10.1136/bmj.i6253

57 Tangri N, Grams ME, Levey AS, *et al.* Multinational assessment of accuracy of equations for predicting risk of kidney failure ameta-analysis. *JAMA - J Am Med Assoc* 2016;**315**:164–74. doi:10.1001/jama.2015.18202

58 ten Haaf K, Jeon J, Tammemägi MC, *et al.* Risk prediction models for selection of lung cancer screening candidates: A retrospective validation study. *PLoS Med* 2017;**14**:1–24. doi:10.1371/journal.pmed.1002277

59 Thangaratinam S, Allotey J, Marlin N, *et al.* Prediction of complications in early-onset pre-eclampsia (PREP): Development and external multinational validation of prognostic models. *BMC Med* 2017;**15**:1–11. doi:10.1097/QAD.0b013e3282efacb1

60 Traeger AC, Henschke N, Hübscher M, *et al.* Estimating the Risk of Chronic Pain: Development and Validation of a Prognostic Model (PICKUP) for Patients with Acute Low Back Pain. *PLoS Med* 2016;**13**:1–21. doi:10.1371/journal.pmed.1002019

61 Van Calster B, Van Hoorde K, Valentin L, *et al.* Evaluating the risk of ovarian cancer before surgery using the ADNEX model to differentiate between benign, borderline, early and advanced stage invasive, and secondary metastatic tumours: Prospective multicentre diagnostic study. *BMJ* 2014;**349**:1–14. doi:10.1136/bmj.g5920

62 Van Den Boogaard M, Pickkers P, Slooter AJC, *et al.* Development and validation of PRE-DELIRIC (PREdiction of DELIRium in ICu patients) delirium prediction model for intensive care patients: Observational multicentre study. *BMJ* 2012;**344**:17. doi:10.1136/bmj.e420

63 Van den Bruel A, Thompson M, Stevens R, *et al.* How well do clinical prediction rules perform in identifying serious infections in acutely ill children across an international network of ambulatory care datasets?. *BMC Med* 2013;**11**:10.http://ovidsp.ovid.com/ovidweb.cgi?T=JS&PAGE=reference&D=medc&NEWS=N&AN=23320738

64 Van Vugt SF, Broekhuizen BDL, Lammens C, *et al.* Use of serum C reactive protein and procalcitonin concentrations in addition to symptoms and signs to predict pneumonia in patients presenting to primary care with acute cough: Diagnostic study. *BMJ* 2013;**346**:1–12. doi:10.1136/bmj.f2450

65 Viera AJ, Garrett JM. Understanding interobserver agreement: The kappa statistic. *Fam Med* 2005;**37**:360–3.

66 W.A. L, R. D, L.A. K, *et al.* Serum protein profiles predict coronary artery disease in symptomatic patients referred for coronary angiography. *BMC Med* 2012;**10**:157. doi:10.1186/1741-7015-10-157

67 Walters K, Hardoon S, Petersen I, *et al.* Predicting dementia risk in primary care: Development and

For peer review only

validation of the Dementia Risk Score using routinely collected data. *BMC Med* 2016;**14**:1–12. doi:10.1186/s12916-016-0549-y

68 Yeh RW, Secemsky EA, Kereiakes DJ, *et al.* Development and validation of a prediction rule for benefit and harm of Dual antiplatelet therapy beyond 1 year after percutaneous coronary intervention. *JAMA - J Am Med Assoc* 2016;**315**:1735–49. doi:10.1001/jama.2016.3775

69 Zemek R, Barrowman N, Freedman SB, *et al.* Clinical risk score for persistent postconcussion symptomsamong children with acute concussion in the ED. *JAMA - J Am Med Assoc* 2016;**315**:1014–25. doi:10.1001/jama.2016.1203

70 Farinati F, Vitale A, Spolverato G, *et al.* Development and Validation of a New Prognostic System for Patients with Hepatocellular Carcinoma. *PLoS Med* 2016;**13**:1–18. doi:10.1371/journal.pmed.1002006

For peer review only

Assessment of study characteristics, used methods and specific reporting items

3 Background and Objectives	
3a: Type of predictive study	1. Prognostic<br>2. Diagnostic
3a: Topic Study	1. (Cardio)vascular<br>2. Oncological<br>3. Surgical<br>4. Other
3b: Model development and validation	1. Development<br>2. Validation<br>3. Development and Validation<br>4. Updating

4 Source of Data	
4a: Study design	1. RCT<br>2. Prospective cohort<br>3. Retrospective cohort<br>4. Nested case-control<br>5. Non-nested case-control

5 Participants	
5a: Study Setting	1. General population<br>2. Primary care<br>3. Secondary care

6 Outcome	
6a: Definition outcome	1. What is the outcome?<br>2. What is the time frame?
6a: Type of outcome	1. Dichotomous,<br>2. Continuous<br>3. Ordinal<br>4. Nominal<br>5. Time to event

9 Missing Data	
9 Type	1. MAR<br>2. MCAR<br>3. MNAR
9 Reason	Reason missing data
9 Handling	1. Complete-case analysis<br>2. Single imputation<br>3. Multiple imputation

For peer review only

	4. Missing indicator method<br>5. Last observation carried forward<br>6. Mean imputation<br>7. Other

10 Statistical Analysis and Methods	
10a: Analysis of predictors	1. Categorical<br>2. Linear: kept linear<br>3. Linear: dichotomized<br>4. Linear: categorized<br>5. Linear: polynomial transformation<br>6. Linear: spline transformation,<br>7. Linear: interaction<br>8. Linear: log-transformation<br>9. Other<br>(multiple options possible)
10b: Type of Model	1. Linear<br>2. Logistic<br>3. Ordinal<br>4. Nominal<br>5. Survival<br>6. Poisson<br>7. Points<br>8. Other
10b: Selection Predictors	1. A priori knowledge<br>2. Based on the literature<br>3. Statistically<br>(multiple options possible)
10b: Model building procedure	1. Entering all predictors<br>2. Forward selection<br>3. Backward selection<br>4. Best subset
10b: Statistical thresholds for model building	1. p-value: threshold<br>2. AIC/BIC<br>3. R^2<br>4. C-statistic/AUC<br>5. HR<br>6. Manually
10b: Internal Validation methods	1. Apparent<br>2. Random split<br>3. Cross-validation<br>4. Bootstrapping<br>(multiple options possible)

For peer review only

10d: External Validation methods	1. Temporal validation<br>2. Geographical validation<br>3. Fully independent<br>4. Other<br>(Multiple options possible)
10d: Performance Calibration	1. Test<br>2. Slope<br>3. Intercept and slope<br>4. Calibration in-the-large<br>5. Plot<br>(multiple options possible)
10d: Performance Discrimination	1. C-statistic / AUC after Development<br>2. C-statistic / AUC after Internal validation<br>3. C-statistic / AUC after external validation<br>4. D-statistic
10d: Performance Classification	1. Sensitivity / Specificity<br>2. Positive Predictive Value / Negative Predictive Value<br>3. Likelihood ratio<br>(multiple options possible)
10d: Performance Other	1. IDI<br>2. NRI<br>3. Decision curve analysis<br>(multiple options possible)
10d: Performance Overall	1. Brier<br>2. 2. R^2
10e: Model Updating	1. Intercept<br>2. all coefficients changed with same factor<br>3. Re-estimation all coefficients independently<br>4. 2+ selection of additional predictors<br>5. 3+ Reestimation all coefficients

13 Participants	
13a: Number of Participants	1. Number of participants with and without outcome
13b: Missing Values	Reported for<br>1. Any value<br>2. Per predictor<br>3. For predictors in general<br>4. For outcome<br>(multiple options possible)

Model Specification	
15a Intercept or baseline hazard	

For peer review only

15a: Coefficients	Coefficients reported:<br>1. All univariate<br>2. Univariate of included predictors in final model<br>3. All multivariate<br>4. Multivariate of predictors included in final model<br>(multiple option possible)
15a: Number of predictors	Number of predictors in final model
15a: Use of model	Use of model reported as:<br>1. Only regression coefficients<br>2. Intercept with regression coefficients<br>3. Simplified score (incl. nomogram or app)<br>(multiple options possible)

For peer review only
